# Identification of mildew resistance in wild and cultivated Central Asian grape germplasm

**DOI:** 10.1186/1471-2229-13-149

**Published:** 2013-10-04

**Authors:** Summaira Riaz, Jean-Michel Boursiquot, Gerald S Dangl, Thierry Lacombe, Valerie Laucou, Alan C Tenscher, M Andrew Walker

**Affiliations:** 1 Department of Viticulture and Enology, University of California, Davis, CA 95616, USA; 2 UMR AGAP, Equipe Diversité et Adaptation de la Vigne et des Espèces Méditerranéennes, Montpellier SupAgro, 2 Place Viala, Montpellier 34060, France; 3 Foundation Plant Services, University of California, Davis, CA 95616, USA; 4 UMR AGAP, Equipe Diversité et Adaptation de la Vigne et des Espèces Méditerranéennes, INRA, 2 Place Viala, Montpellier 34060, France

**Keywords:** Powdery mildew resistance, *Vitis vinifera* subsp. *sativa*, *Vitis vinifera* subsp. *sylvestris*, Gene flow

## Abstract

**Background:**

Cultivated grapevines, *Vitis vinifera* subsp. *sativa*, evolved from their wild relative, *V. vinifera* subsp. *sylvestris*. They were domesticated in Central Asia in the absence of the powdery mildew fungus, *Erysiphe necator*, which is thought to have originated in North America. However, powdery mildew resistance has previously been discovered in two Central Asian cultivars and in Chinese *Vitis* species.

**Results:**

A set of 380 unique genotypes were evaluated with data generated from 34 simple sequence repeat (SSR) markers. The set included 306 *V. vinifera* cultivars, 40 accessions of *V. vinifera* subsp. *sylvestris*, and 34 accessions of *Vitis* species from northern Pakistan, Afghanistan and China. Based on the presence of four SSR alleles previously identified as linked to the powdery mildew resistance locus, *Ren1*, 10 new mildew resistant genotypes were identified in the test set: eight were *V. vinifera* cultivars and two were *V. vinifera* subsp. *sylvestris* based on flower and seed morphology. Sequence comparison of a 620 bp region that includes the *Ren1*-linked allele (143 bp) of the co-segregating SSR marker SC8-0071-014, revealed that the ten newly identified genotypes have sequences that are essentially identical to the previously identified mildew resistant *V. vinifera* cultivars: ‘Kishmish vatkana’ and ‘Karadzhandal’. Kinship analysis determined that three of the newly identified powdery mildew resistant accessions had a relationship with ‘Kishmish vatkana’ and ‘Karadzhandal’, and that six were not related to any other accession in this study set. Clustering procedures assigned accessions into three groups: 1) Chinese species; 2) a mixed group of cultivated and wild *V. vinifera*; and 3) table grape cultivars, including nine of the powdery mildew resistant accessions. Gene flow was detected among the groups.

**Conclusions:**

This study provides evidence that powdery mildew resistance is present in *V. vinifera* subsp. *sylvestris*, the dioecious wild progenitor of the cultivated grape. Four first-degree parent progeny relationships were discovered among the hermaphroditic powdery mildew resistant cultivars, supporting the existence of intentional grape breeding efforts. Although several Chinese grape species are resistant to powdery mildew, no direct genetic link to the resistance found in *V. vinifera* could be established.

## Background

The detection of resistance to *Erysiphe necator,* the causal agent of grape powdery mildew, in two cultivars of *Vitis vinifera* from Central Asia [1,2] was intriguing given that this fungus was thought to have co-evolved with North American grape species, and that all *V. vinifera* cultivars were considered to be susceptible to this fungus. This discovery suggests that powdery mildew resistance is more complex than once thought and that other grape species may have played a role in the resistance found in these Central Asian cultivars. Several grape species native to Central Asia and China are known to express powdery mildew resistance [3,4], leading one to question the historical presence of powdery mildew in Asia and the role Asiatic species might have played in the evolution of resistance in present day cultivated grapes. Addressing these questions would provide insight into the evolution of powdery mildew resistance and the forces driving grape diversity.

It is widely accepted that the cultivated form of *V. vinifera* subsp. *sativa* derived from its wild form *V. vinifera* subsp. *sylvestris*[5,6], which was once spread widely across Western Europe, the Mediterranean, the Caucasus, Himalaya and Hindu Kush mountain ranges, and Central Asia [7-10]. The mountainous region between the Caucasus and China is considered the center of diversity for many temperate fruit crops [11-13]. Transitional types of grapes that included wild forms of the subsp. *sylvestris*, feral and cultivated land races and ancient local varieties were once common in this region [14-16].

One of the key features separating domesticated grapes from their wild relatives is their reproductive system. Wild relatives (*V. vinifera* subsp. *sylvestris*) are dioecious with anemophilous pollination while the domesticated grapevine is hermaphroditic in nature [5]. However, the origin and evolution of hermaphrodism in grape remains an open question. It is not known whether hermaphrodism evolved through sexual recombination, as a mutation of the wild form that was then introgressed into cultivated varieties, or as a mutation that originated in cultivated forms. Cultivated grapevines have a very wide range of variation in fruit, leaf and growth characteristics, and there are thousands of varieties found worldwide [5,17]. The high amount of diversity is due to the long history of grapevine cultivation that dates back to 4000 – 6000 BC [8,18,19]. Initially grape cultivation relied on both seed and vegetative propagation and was influenced by religion, regional traditions and human migration [18,20]. Seeds may have been the more common means of propagation early in the cultivation of grapes as they were easier to transport over large distances and intentional and unintentional crosses generated great diversity within the cultivated types [21].

Historical records of grape growing in the Orient and Central Asia are very limited [18], however there is no indication of powdery mildew in available records from this region of the world. Powdery mildew, caused by *Erysiphe necator,* was first described on grapes in North America in 1834. It was discovered in Europe in 1845 [22] and by 1852, it was reported throughout Europe and the Mediterranean region [23]. Considering the long history of viticulture, the great attention paid to wine grapes, and the lack of any mention of this disease in historical records, it is unlikely that *E. necator* existed in Europe prior to the early 1800s. Frequent trade activity, including the exchange of plant material, facilitated the rapid spread of *E. necator* over long distances. Many North American *Vitis* species are resistant to mildew diseases and other pests [15,24]. Their resistance to powdery mildew is attributed to coevolution with this fungal disease. On the other hand, the Central Asian forms of *V. vinifera* subsp. *sativa* were domesticated in the absence of powdery mildew pressure in the mountains of Caucasus and surrounding areas, and these grapes lack resistance to powdery mildew. In the early to mid-1900s, extensive grape breeding programs were maintained in multiple states of the former Soviet Union, which used germplasm acquired from Central Asia, China, the trans-Caucasus region, Africa, and Europe. Powdery mildew resistance was an important goal for these breeding programs [25,26] and resistance from the Chinese species, especially *V. amurensis* was introgressed into cultivated varieties [3,27]. There are no historical records that indicate any other powdery mildew resistant Chinese species were part of grape breeding in early 1900s [4].

China was linked to Central Asia by both northern and southern silk routes and grape culture was flourishing by the second century AD [19,28]. Although there are many diverse grape species in China, their impact on grape domestication is unknown [29]. This is in part due to the inaccessibility of germplasm, and historical and scientific records to the non-Chinese speaking world. The presence of powdery mildew resistance in Chinese grape species is unexplained. We do not know whether these species acquired resistance to fungal diseases after introduction of the disease from the New World (over the past 150 to 400 years) or whether powdery mildew and other fungal diseases were present in Asia for a longer time period, but was not recorded in accessible historical records. The records available regarding grape breeding in Central Asia are limited to the early 1900s when the renowned Russian geneticist Nikolai I. Vavilov initiated germplasm acquisition trips in Central Asia and neighboring regions [11].

There were three major objectives to this investigation into the origins of powdery mildew resistance in cultivated *V. vinifera* subsp. *sativa*. The first was to evaluate a large collection of cultivated *V. vinifera* germplasm from Central Asia to identify additional powdery mildew resistant accessions using simple sequence repeats (SSR) markers linked to the powdery mildew resistance locus *Ren1* on chromosome 13 [1,2]. We speculated that since there was breeding for powdery mildew resistance in Central Asia before the mid-1900s, there might be undocumented resistant selections, resistant parental material or new sources of resistant germplasm from this region. We analyzed accessions maintained in two of the world’s largest grape germplasm repositories [17,30], and the Department of Viticulture and Enology, and Foundation Plant Services at the University of California, Davis. The second objective was to evaluate the powdery mildew resistance in a range of Chinese *Vitis* species and in accessions of *V. vinifera* subsp. *sylvestris* collected from the regions of grapevine domestication, to determine if they were resistant to the disease and to identify potential contributors of powdery mildew resistance in Central Asian grape cultivars. An analysis of population structure and diversity was conducted to obtain a global perspective on the mechanisms of domestication, and gene flow from accessions of wild Chinese species and *V. vinifera* subsp. *sylvestris* in an effort to determine the source of powdery mildew resistance detected in Central Asian *V. vinifera* cultivars. North American species and complex hybrids of these species resistant to powdery mildew were also included to determine their possible role in the resistance detected in Central Asian *V. vinifera* accessions. The third objective was to unravel potential parent-progeny relationships using kinship analysis to broaden the family of powdery mildew resistant cultivars for use by grape breeders.

## Results

### Identifying the unique germplasm set and SSR allele data

**Figure 1:**
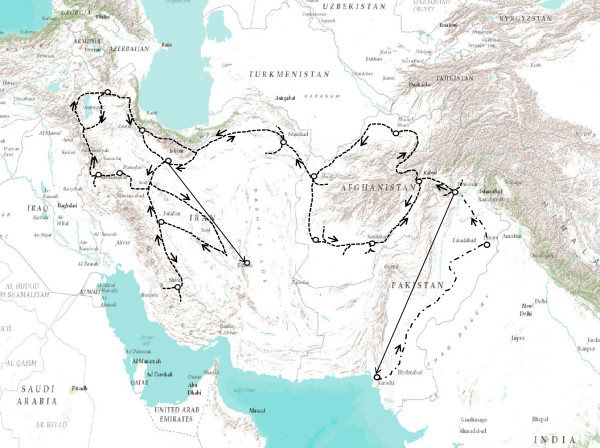
**A map of the areas traversed by Dr. Harold P. Olmo in 1948 during a Central Asian germplasm acquisition trip.** Straight lines represent travel by air, dashed lines travel by automobile, horse and burro, and dashed lines broken with dots are travel by train. He spent one year travelling over 12,000 miles while collecting 775 extremely valuable accessions of fruit and nut varieties in Iran, Afghanistan and Pakistan. He collected both seeds and cuttings during this trip, which were sent back to the USA at regular intervals.

This study utilized grape accessions maintained in two of the world’s largest germplasm repositories: the INRA Domaine de Vassal (Fance) collection; and combined collections housed at Davis, California (University of California, Davis and the National Clonal Germplasm Repository). Most of the University of California, Davis accessions were collected by Harold P. Olmo (Department of Viticulture and Enology, University of California, Davis) during germplasm acquisition travels in 1948 (Table 1, Additional file 1: Table S1, Figure 1). Many of the 559 accessions tested shared identical marker profiles, thus suggesting possible cases of synonymies were observed within and among the samples from the two collections (Additional file 2: Table S2). Further analysis was based on 403 unique accessions: 296 from the Davis collections and 107 from the INRA Domaine de Vassal germplasm collection. Additional file 3: Table S3 presents the fingerprint profiles of the 403 unique accessions based on 19 SSR markers – one marker from each grape chromosome.

**Table 1:** List of evaluated germplasm with geographical region and source country

**Group**	**Geographical region**	**Source countries**	**Number of samples**
***V. vinifera *subsp. *sativa***	Balkans, Russia and USSR, Ukraine	Yugoslavia, Greece, Russia, USSR	110
	Eastern Mediterranean and Caucasus	Armenia, Azerbaijan, Egypt, Israel, Lebanon, Turkey,	29
	Middle and Far East	Afghanistan, China, India, Iran, Iraq, Japan, Kazakhstan, Pakistan, Tajikistan, Turkmenistan, Uzbekistan, Yemen	266
	New World	USA	3
	Western Europe	France	2
	Unknown		51
***V. vinifera *subsp. *sylvestris***	Eastern Mediterranean and Caucasus	Armenia, Georgia,	16
	Middle and Far East	Turkmenistan, Afghanistan, Iran	27
**Other Species and hybrids**			
*V. amurensis*		China, Korea, USSR	8
*V. betulifolia*		China	1
*V. coignetiae*		USSR, unknown	4
*V. ficifolia*		China, South Korea	4
*V. flexuosa*		Unknown	1
*V. jacquemontii*		Pakistan	5
*V. lanata*		Afghanistan	1
*V. piasezkii*		China, unknown	3
*V. romanetii*		China	4
*V. yenshanensis*		China	3
*Vitis* species (unknown)		China	4
*M. rotundifolia*		USA	2
*Ampelopsis delavayana*		Unknown	1
*V. riparia*		USA	1
Interspecific hybrids		France, Russia, USA	13
		**Total**	**559**

Twenty-three accessions (interspecific hybrids, *Vitis riparia, Muscadinia rotundifolia*, three reference European winegrape varieties, and eight other accessions with missing data at 7 or more loci) were removed from the study set. The number of alleles per marker and percent of missing data were calculated for the remaining 380 genotypes with 34 markers (Table 2, Additional file 4: Table S4). Based on the collection records, the study set of 380 unique accessions consisted of 306 genotypes of *V. vinifera* subsp. *sativa*, 40 accessions of *V. vinifera* subsp. *sylvestris*, and 34 accessions of *Vitis* species from northern Pakistan, Afghanistan and China. A minimum of 9 and maximum of 44 alleles were observed with SSR markers VVIq52 and VVIv67, respectively. The average number of alleles for all markers was 22. There were 7 markers with 5% or more accessions that had missing data (Table 2).

**Table 2:** **Number of alleles, percentage of missing data (MD), observed (Ho) and expected (He) level of heterozygosity, and F**_**IS **_**in three germplasm groups**

**Marker name**	**Total**		**Group A (Species)**			**Group B (O34-16)**			**Group C (TSL)**			**F**_**ST**_
	**MD (%)**	**Alleles**	**Ho**^**a**^	**He**^**b**^	**F**_**IS**_	**Ho**	**He**	**F**_**IS**_	**Ho**	**He**	**F**_**IS**_	
VVIp60	**12.53**^**c**^	25	0.55	0.93	0.41	0.65	0.78	0.17	0.69	0.74	0.07	0.042
VVIb01	8.35	19	0.81	0.88	0.08	0.66	0.65	**−0.01**	0.67	0.61	−0.10	0.120
VVMD28	2.46	31	0.78	0.92	0.16	0.82	0.89	0.07	0.80	0.83	0.04	0.046
VVMD32	3.69	24	0.62	0.89	0.31	0.80	0.87	0.08	0.83	0.79	−0.04	0.057
VMC4c6	2.95	15	0.30	0.82	0.64	0.58	0.69	0.15	0.70	0.78	0.10	0.036
VrZAG79	1.97	20	0.62	0.94	0.34	0.76	0.82	0.07	0.86	0.78	−0.10	0.056
VVMD27	2.70	21	0.93	0.90	**−0.03**	0.72	0.85	0.15	0.82	0.75	−0.09	0.052
VMC2g2	3.69	15	0.64	0.87	0.26	0.75	0.77	0.03	0.75	0.67	−0.12	0.048
VVMD21	9.34	21	0.62	0.75	0.18	0.66	0.79	0.16	0.68	0.73	0.06	**0.014**
VrZAG62	2.95	20	0.62	0.83	0.26	0.80	0.87	0.08	0.79	0.79	−0.01	0.044
VVMD31	6.39	20	0.59	0.90	0.34	0.66	0.76	0.13	0.70	0.72	0.04	0.025
VVMD7	**0.98**	21	0.61	0.82	0.26	0.70	0.83	0.16	0.87	0.86	−0.01	0.053
VMC1b11	2.95	19	0.61	0.81	0.25	0.78	0.83	0.07	0.74	0.73	−0.01	0.054
VVIq52	4.18	**9**	0.26	0.82	**0.68**	0.57	0.70	0.18	0.69	0.68	−0.02	0.036
VVIv37	3.93	22	0.64	0.93	0.31	0.74	0.89	0.16	0.70	0.86	**0.18**	0.016
VVMD25	2.46	21	0.89	0.93	0.04	0.77	0.79	0.02	0.88	0.80	−0.10	0.030
VVS02	1.47	18	0.76	0.90	0.15	0.81	0.88	0.08	0.90	0.87	−0.03	0.038
VMC4f3.1	1.47	34	0.86	0.94	0.08	0.85	0.92	0.07	0.83	0.84	0.02	0.040
VMC8g9	3.69	24	0.66	0.89	0.26	0.71	0.81	0.12	0.77	0.77	−0.01	0.031
VVIh54	3.93	26	0.86	0.92	0.06	0.68	0.80	0.14	0.71	0.74	0.04	0.033
VMCNg4e10.1	2.95	23	0.79	0.89	0.11	0.84	0.87	0.03	0.92	0.86	−0.06	0.038
sc47-18	3.44	30	0.85	0.94	0.10	0.79	0.91	0.12	0.90	0.90	0.00	0.027
SC08-0071-014	4.42	26	0.77	0.93	0.17	0.71	0.86	0.17	0.79	0.79	0.00	0.035
UDV124	10.57	35	0.79	0.90	0.12	0.85	0.91	0.07	0.85	0.81	−0.05	0.052
VMC3d12	5.90	31	0.86	0.94	0.08	0.82	0.90	0.09	0.89	0.88	−0.01	0.027
VVMD24	2.95	17	0.64	0.77	0.17	0.69	0.74	0.05	0.75	0.76	0.02	0.060
VVIv67	7.62	**44**	0.38	0.91	0.59	0.66	0.84	0.20	0.60	0.72	0.16	0.056
VVMD5	1.72	20	0.72	0.89	0.18	0.82	0.87	0.05	0.83	0.80	−0.04	0.040
VVIn73	2.95	10	0.59	0.74	0.19	0.49	0.52	0.06	0.49	0.56	0.12	0.080
UDV108	4.67	33	0.86	0.94	0.08	0.67	0.87	**0.23**	0.70	0.77	0.09	0.036
VMC7f2	5.41	13	0.69	0.69	0.00	0.63	0.69	0.09	0.71	0.74	0.04	0.023
VVIn16	4.42	13	0.50	0.66	0.24	0.51	0.62	0.18	0.69	0.67	−0.03	0.117
VMC2g6	3.19	10	0.41	0.49	0.15	0.35	0.39	0.10	0.41	0.37	−0.11	**0.201**
VVIp31	2.70	21	0.70	0.90	0.22	0.87	0.91	0.05	0.89	0.88	**0.00**	0.025
Average	4.26	22			0.223			0.109			0.002	0.050

^a^No. of hterozygotes at a locus/no. of individuals typed.

^b^Expected heterozygosity was computed using Nei (1987).

^c^Bold font within the table indicates maximum and minimum values.

Estimation of F_IS_ and F_ST_ was computed as detailed by Weir & Cockerham (1984).

### Search of germplasm resistant to powdery mildew and disease evaluation

Prior to phenotypic evaluation for powdery mildew resistance, the entire set of 403 accessions was genotyped for linkage with the powdery mildew resistance locus *Ren1* at four SSR marker, which span 8.1 cM genetic block on chromosome 13 (VMCNg4e10.1, sc47-18, *Ren1* locus*,* SC08-0071-01 and UDV124) [1,2]. The resistance-linked allele of 260 (bp) defined by marker VMCNg4e10.1 was observed in 47 accessions that included *V. amurensis*, *V. romanetii*, *Muscadinia rotundifolia* and wild *V. vinifera* subp. *sylvestris* accessions. The resistance-linked allele of 216 (bp) defined by UVD124 marker was present in 45 accessions. The majority of the accessions with allele 260 for marker VMCNg4e10.1 did not have allele 216 at the marker UDV124. However, a missing allele at either marker could have been due to a recombination event. Eleven accessions, including ‘Karadzhandal’ and ‘Kishmish vatkana’ had alleles that are in linkage with the *Ren1* locus at the two distal markers, VMCNg4e10.1 and UDV124 that are in linkage with the *Ren1* locus (Additional file 5: Table S5). The germplasm set was then evaluated for alleles at sc47-18 and SC08-0071-014 that flank each side and co-segregates with the *Ren1* locus [2]. ‘Karadzhandal’ and ‘Kishmish vatkana’ fingerprint profiles were used to determine whether the allele of 249 (bp) defined by the marker sc47-18 and the allele of 143 (bp) defined by the marker SC08-0071-014 were linked to resistance. These are the two alleles that co-segregate with the *Ren1*. Allele 249 for marker sc47-18 was common: 80 of the 403 accessions shared it. Nearly all of the 47 accessions that carried the allele 260 for marker VMCNg4e10.1 also had allele 249 for marker sc47-18, confirming the tight linkage between these two markers (Additional file 5: Table S5). The allele 143 for marker SC08-0071-014 was rare; only 17 accessions in the entire data set carried it (Additional file 5: Table S5). Six accessions had the allele 143 for marker SC8-0071-014, but did not carry the resistance-associated alleles at all tested markers (Additional file 5: Table S5). ‘Khalchili’ and ‘Khwangi’ had 143 flanked by 249 at sc47-18. Two of the *V. vinifera* subsp. *sylvestris* accessions had alleles 143 and 260 on the opposing flanks for marker VMCNg4e10.1; ‘Matrassa’ and a third *sylvestris* accession had the 143 allele and no resistance-associated allele on the opposite flank. These six accessions are potential recombinants. Two Chinese species accessions, *V. romanetii* (C166-043) and *V. yenshanensis* (588421.a) had the 143 allele, but neither of them carried a resistance-associated allele at the other three markers. In the case of these two examples, we speculate that the presence of the 143 allele is either due to size homoplasy, or the alleles at other markers are lost due to recombinations.

In 2009 and 2010, resistance to powdery mildew was evaluated on a 0 to 5 scale (no symptoms to severe symptoms) on all accessions from Davis that carried alleles linked to resistance at one or more markers (Table 3, Additional file 6: Table S6). Four other accessions were evaluated in 2012 (data not shown). The year effect was significant; disease pressure was more severe in 2010 (Table 3). However, the susceptible controls were highly susceptible (> 4 on the 5 point scale) and resistant controls had no or minor symptoms in three test years (Additional file 6: Table S6). The location in the field test plot was not significant, indicating that the close-spaced field evaluation site with no spray was an efficient and cost effective way to screen for resistance (Table 3). ‘Karadzhandal’, a known powdery mildew resistance accession was evaluated both years and had a powdery mildew resistance score of < 1. ‘Kishmish vatkana’ the other previously known powdery mildew resistant accession was under quarantine as part of the importation process and could not be evaluated. Accessions with *Ren1*-linked alleles at only one of the flanking markers exhibited no resistance to powdery mildew in the field test (Additional file 5: Table S5, Additional file 6: Table S6). The *V. vinifera* subsp. *sativa* cultivars: ‘Husseine’, ‘Khalchili’, ‘Late Vavilov’ and ‘Sochal’ were resistant. These four accessions had mean scores for leaf and cane PM symptoms ranging from 1.08 - 2.42 in 2009 and 0.83 – 2.42 in 2010 (Additional file 6: Table S6). In 2012, in a much smaller evaluation, two accessions from wild *V. vinifera* subsp. *sylvestris* were identified as resistant from field evaluations: O34-16, collected from Shiravan, Iran, had all four resistance-linked alleles; and DVIT3351.27, collected from Armenia, had the resistance allele at one of the flanking markers on each side. Mean leaf symptoms scores were 0.4 and 0.7 for DVIT3351.21 and O34-16, respectively (Additional file 6: Table S6).

**Table 3:** Results of two seasons of field-based powdery mildew evaluations with selected accessions

		**A**			**B**			**C**			
		**Genotype**	**Date**	**Bed**	**Genotype**	**Date**	**Bed**	**Genotype**	**Date**	**Bed**	**Year**
**Leaf**	Numb.parm.	64	1	2	43	1	4	8	1	4	1
	DF	64	1	2	43	1	4	8	1	4	1
	L-R *χ*^2^	809.51	11.10	2.70	528.44	21.78	5.16	249.31	0.01	7.71	12.19
	P-value	<0.0001*	0.0009*	0.2594	<0.0001*	<0.0001*	0.2715	<0.0001*	0.9094	0.1029	0.0005*
**Cane**	Numb.parm.	64	1	2	43	1	4	8	1	4	1
	DF	64	1	2	43	1	4	8	1	4	1
	L-R *χ*^2^	744.91	16.05	4.30	290.99	412.48	1.97	137.04	22.09	1.80	64.62
	P-value	<0.0001*	<0.0001*	0.1163	<0.0001*	<0.0001*	0.7416	<0.0001*	<0.0001*	0.7731	<0.0001*

Control plants of known resistant and susceptible accessions were used for both years. (A) Results for all accessions tested in 2009; (B) results for accessions tested in 2010; (C) Comparison of accessions that were common to both years. The number of field nursery beds and genotypes are noted.

*Indicates statistically significant P values.

The powdery mildew resistant accession ‘Karadzhandal’ at Davis had similar marker profile to accession ‘Kara djandjal’ and the newly identified resistant accession ‘Husseine’ had identical marker profile to ‘Kandari noir’ in the Vassal collection. Both of these accessions were found to be resistant in greenhouse screens carried out at Vassal. ‘Chirai oback’ and ‘Vassarga tchernaia’, which had all four *Ren1*-linked SSR marker alleles, were resistant based on greenhouse screening in France. The other two powdery mildew resistant accessions were ‘Soïaki’, which had resistance-linked alleles with four markers, and ‘Matrassa’, which had resistance-linked alleles with two markers on one side, have not been evaluated for disease symptoms. In total, this study identified and verified eight new accessions that are powdery mildew resistant. ‘Soïaki’ and ‘Matrassa’ were identified as potentially resistant based on marker analysis. Their disease resistance needs to be verified in a field or greenhouse screen.

### Probability of identity and parent relationships

Probability of identity analysis found that nine markers were sufficient to identify unique accessions in the study set (Additional file 7: Table S7). The paternity exclusion probability for a single locus ranged from 10.6% (VMC4c6) to 72.6% (VVIv67) (Additional file 7: Table S7). A cumulated probability of exclusion of 100% was reached using only 7 markers for paternity and 3 markers for a parent pair. The simulation for parentage analysis identified a LOD score threshold of 5.0 to assess a potential single parent and 4.0 to assess a parent pair with 34 SSR markers. Six newly identified resistant accessions ‘Husseine’, ‘Chirai obak’, DVIT3351.27, O34-16, ‘Soïaki’ and ‘Matrassa’ were not related to any other accession in the set; two of these are *V. vinifera* subsp*. sylvestris*. The presence of powdery mildew resistance in unrelated genetic backgrounds is a very important result of this study that suggests that powdery mildew resistance in Central Asia is complex and potentially represent orthologous (diverged after a speciation event) and paralogous (diverged after a duplication event) homology for the *Ren1* locus, first identified in ‘Karadzhandal’ and ‘Kishmish vatkana’ [1,2]. A second important inference from these results is that there may be many more powdery mildew resistant accessions within *V. vinifera* subsp. *sativa* and Central Asian *Vitis* species and further exploration is needed. This resistance could be the result of intentional breeding efforts involving material collected and curated in the early 1900s at multiple institutes set up by the Russian geneticist Vavilov, or the result of unintentional breeding and selection of resistant material in an earlier period of domestication and selection over thousands of years. More importantly, identification of powdery mildew resistance in accessions of *V. vinifera* subsp. *sylvestris* indicates that the resistance is present in wild germplasm.

**Figure 2:**
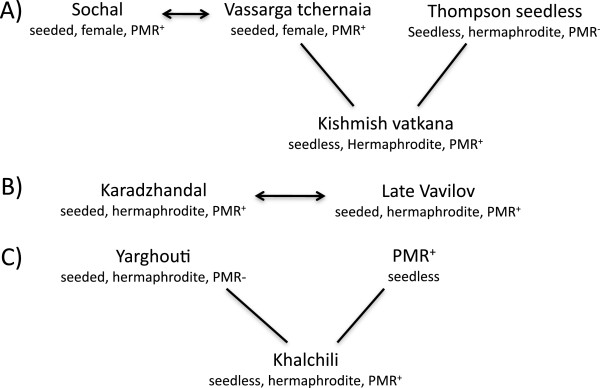
**Four new first-degree relationships between powdery mildew resistant accessions discovered in the study. (A)** We identified two first-degree relationships in relation to ‘Kishmish vatkana’. ‘Vassarga tchernaia’ was identified as the female parent of 'Kishmish vatkana' and ‘Sochal’ as parent to ‘Vassarga tchernaia’. We also verified ‘Thompson seedless’ as the male parent based on the analysis with 34 SSR markers. **(B)** The third first-degree relationship was detected between ‘Karadzhandal’ and ‘Late Vavilov’; both are hermaphrodite with seeded fruit. **(C)** A fourth parent progeny relationship was detected between ‘Yarghouti’ and ‘Khalchili’.

There were four parent-progeny relationships identified in this study; two involved the previously published powdery mildew resistant accessions (Figure 2). ‘Vassarga tchernaia’ was conclusively identified as the female parent of ‘Kishmish vatkana’; it also shared a parent-progeny relationship with ‘Sochal’ although the direction of the cross is unknown. ‘Karadzhandal’ and ‘Late Vavilov’ share one or both alleles with all 42 SSR markers (Additional file 8: Table S8). The powdery mildew resistant accession ‘Khalchili’ was shown to have a first-degree relationship to ‘Yarghouti’ (Figure 2, Additional file 8: Table S8).

### Sequencing of the resistance-linked allele of an SSR marker that co-segregates with *Ren1*

**Figure 3:**
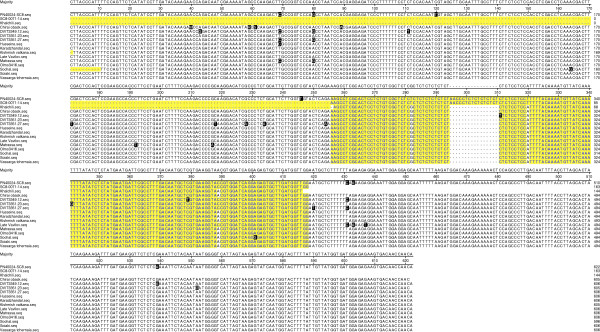
**Sequence comparison of a 620 (bp) region associated with the 143 allele of SSR marker ‘SC-0071-014’ that cosegregates with the *Ren1 *locus.** The yellow highlighted area represents the sequence of the 143 (bp) allele. All of the newly identified powdery mildew resistant accessions have the 143 allele with marker ‘SC-0071-014’ (See Table S5).

A 620 bp region that includes the resistance associated 143 bp allele from marker SC8-0071-014 was sequenced for the 12 powdery mildew resistant accessions and two susceptible *V. vinifera* subsp. *sylvestris* (Figure 3). Two accessions of Chinese species that had a 143 bp fragment at SC8-0071-014 were also sequenced. The sequences were nearly identical for all fourteen *V. vinifera* accessions except for occasional single nucleotide polymorphisms (SNPs) between both unrelated and genetically related accessions. The sequences of the two Chinese species, *V. romanetii* (C166-043) and *V. yenshanensis* (588421.a) were very different from one another and from the *V. vinifera* sequence, obvious examples of size homoplasy, where two alleles are identical in size but result from independent events (data not shown).

### Expanded genetic analysis

**Figure 4:**
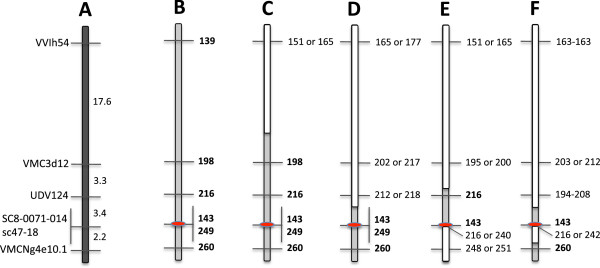
**Reconstruction of the resistant haplotype for a 26 cM region of chromosome 13. (A)** The reference genetic map for this region shows the order and distance between six SSR markers that map around the *Ren1* locus. **(B)** At these markers the allele lengths (bp, bold) of the resistant haplotype, inferred from the two previously identified resistant accessions ‘Kishmish vatkana’ and ‘Karadzhandal’ are also identical to those of six of the resistant accessions identified in this study (*Ren1* location in red). Three of these accessions, ‘Sochal’, ‘Vassarga tchernaia’ and ‘Late Vavilov’ are related to the two previously identified accessions, which strongly implies the powdery mildew resistance is derived from the similar ancestral lineage. **(C)** The allelic composition of the powdery mildew resistant accession ‘Chirai obak’ shows a recombination event between marker VMC3d12 and VVIh54, and **(D)** A recombination occurred between marker SC8-0071-014 and UDV124 for ‘Khalchili’. **(E)** ‘Matrassa’ shows patterns with double recombinations. **(F)** ‘DVIT3351.27’ has complex allelic pattern and may have different genetic background for powdery mildew resistance.

For the 12 powdery mildew resistant accessions, including the two previous known powdery mildew resistant accessions ‘Kishmish vatkana’ and ‘Karadzhandal’, genetic analysis was expanded to a 26 cM genomic block with six SSR markers including the *Ren1* region (Figure 4). Six of the newly identified powdery mildew resistant accessions, including O34-16, a *V. vinifera* subsp. *sylvestris*, had similar alleles similar to ‘Kishmish vatkana’, and ‘Karadzhandal’ with six SSR markers (Figure 4). SSR marker allelic comparison of two other resistant accessions indicated that a recombination event had occurred between markers at different junctions. The wild subsp. *sylvestris* accession, DVIT3351.27 had complex allelic combination of markers surrounding the *Ren1* region suggesting a different genetic origin of powdery mildew resistance.

### Genetic diversity

**Figure 5:**
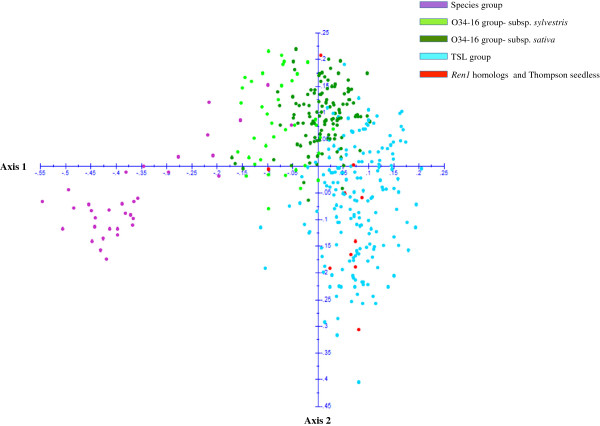
**Principle Coordinate Analysis constructed with genotypic data from 34 SSR markers on 380 accessions using DARWIN software.** Axis 1 and 2 represent 4.36 and 3.27 percent of the variation, respectively.

**Figure 6:**
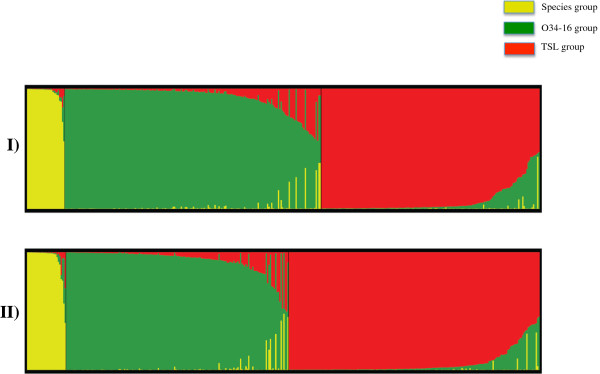
**Graphical presentation of the results obtained from STRUCTURE using K = 3.** Each individual is shown as a vertical line partitioned into segments representing the estimated coefficients of membership proportions in the three ancestral genetic clusters inferred with STRUCTURE. Individuals within each cluster are arranged according to estimated cluster membership proportions (Q-value). Detail of accessions in each cluster is provided in Table S9.

The genetic diversity of the core set of 380 Central Asian accessions was evaluated with hierarchical clustering (Ward method), principle coordinate analysis (PCoA), and a model-based clustering method implemented in the program STRUCTURE. All three analysis methods generated three groups with data from 19 (one from each chromosome) or 34 markers. The delta K value calculated from the output of STRUCTURE was 45.0 at K = 3 compared to less than 5.0 at all other values of K. The three groups determined by PCoA were similar to those produced by STRUCTURE (Figure 5, Figure 6). The Q-values (proportion of a given individual’s genome that originated from a given population) assigned by STRUCTURE for 380 accessions in three groups are displayed in Additional file 9: Table S9.

Group A (Species) contained 29 *Vitis* species accessions, nearly all of which originated in China. The Q-value for membership in this group was 0.90 or above for 25 accessions. *Vitis yenshanensis* (588421.a) and B-166-016, an accession labeled as *Vitis* spp., both collected from China had Q-values split between the group A and B. A second *V. yenshanensis* (588422.a) accession, also collected from China, had Q values split between group A and C. ‘Khir Ghuluman’, collected by H.P. Olmo in Afghanistan, had Q-values split among all three groups indicating it was a possible hybrid of a local species and cultivated varieties. ‘Khir Ghuluman’ was labeled as *V. vinifera*, presumably because it is a cultivated variety in Afghanistan. None of the previous and newly identified powdery mildew resistant accessions were in this group.

Group B (O34-16) contained 165 samples, a mix of both *V. vinifera* cultivars and wild *V. vinifera* subsp. *sylvestris* accessions. Three new powdery mildew resistant accessions were placed in this group with Q-values of 0.80 and higher. O34-16 and DVIT3351.27 were collected as subsp. *sylvestris*, and ‘Matrassa’ (2642Mtp2) was collected as subsp. *sativa*. For clarity, this group will be referred to as O34-16 from this point onward in the manuscript. Thirty-nine accessions collected as subsp. *sylvestris* were in this group, 36 of which had Q-values of 0.80 or higher. Two Chinese *Vitis* species accessions, 588650.a (*V. yenshanensis*) and B-166-019 (labeled *V. sp.*) were in this group, and are most likely hybridized forms. All four accessions of *V. jacquemontii* collected from Pakistan were also in this group. The Q values of these accessions, one as high as 0.97, suggest that these accessions are not pure species and may be hybrid or mislabeled forms (Additional file 9: Table S9).

Group C (TSL) was named after ‘Thompson seedless’ to indicate that the group consists primarily of table grape cultivars. Two previously identified and seven of the new powdery mildew resistant accessions were in this group (Additional file 9: Table S9). The group consisted of 185 accessions labeled as *V. vinifera* subsp. *sativa* and one accession of subsp. *sylvestris* – O35-64 collected from Iran by H.P. Olmo. The Q values for this particular accession placed it in groups A and C. ‘Kala Khostan’ with a group C Q-value of 0.66 is the only other accession in this group with association to the species group. All but 10 of the remaining 184 accessions had group TSL Q-values of 0.70 or higher (Additional file 9: Table S9).

The results of the PCoA analysis with 34 markers also produced three groups. The species group was clearly separated from the other two groups. The distinction between the O34-16 group and the TSL group was less clear (Figure 5). The O34-16 group contained nearly all of the subsp. *sylvestris* accessions, however within that group, there was no clear distinction between the cultivated and wild forms (Figure 5).

**Figure 7:**
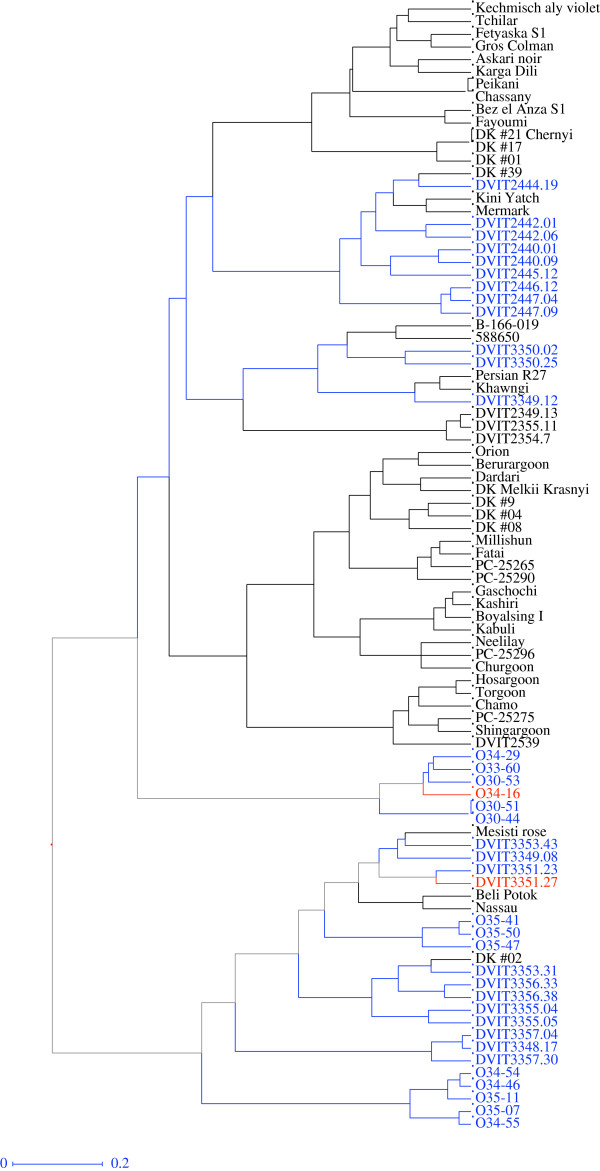
**Dendrogram of 89 accessions in the O34-16 group based on hierarchal cluster analysis (Ward method) using the simple dissimilarity matrix derived from 34 SSR markers.** Accession names or ID in blue font are *V. vinifera* subsp. *sylvestris* based on collection records. Two accessions in red font were resistant to powdery mildew in the field trials; both are *V. vinifera* subsp. *sylvestris* based on flower phenotype and seed morphology.

To differentiate the wild *sylvestris* accessions from cultivated *sativa* forms, further analyses focused on only the O34-16 group. The Ward and UPGMA hierarchical clustering methods divided the 165 accessions of the O34-16 group into two clades. Seventy-six cultivated *V. vinifera* subsp. *sativa* accessions, including the powdery mildew resistant ‘Matrassa’ were in one group, and the second group consisted of 89 *sativa* and *sylvestris* accessions. When the Ward clustering method was applied to this second group of mixed accessions, there were again two clades. Twenty of the *sylvestris* accessions grouped with four cultivated accessions (‘Beli Potok’, ‘Nassau’, ‘DK#2’, and ‘Mesisti rose’) (Figure 7). These four *V. vinifera* accessions are ancient cultivars and are likely transitional forms with the wild ancestor *sylvestris*. The powdery mildew resistant accession DVIT3351.27 was in this clade. The second clade had three less well-defined sub groups: the first contained six *sylvestris* accessions including the powdery mildew resistant accession O34-16; the second group contained accessions of *V. vinifera* subsp. *sativa* collected from Pakistan and Turkmenistan and one accession labeled *V. jacquemontii*; and the third group was a mix of wild *sylvestris* or feral types that were collected from Iran, Iraq, Turkmenistan, Pakistan and Russia, and five incorrectly identified accessions – three of which were labeled as *V. jacquemontii,* and the other two were collected from China.

Two distinct clades were revealed when clustering analysis (Ward method) was applied to the 40 wild *sylvestris* accessions (Additional file 10: Figure S1). The first clade contained ten *sylvestris* accessions obtained from Turkmenistan, four accessions from Iran and two from Afghanistan. The Turkmenistan accessions were collected from the Kopet Dag mountain range, which defines the border between Turkmenistan and Iran on the east of the Caspian Sea. The powdery mildew resistant accession O34-16, collected near the town of Shirvan, Iran, was in this clade. Shirvan is near Mashhad, an important trade hub on the ancient silk route located on the other side of the Kopet Dag mountain range (Figure 1). Therefore, it was not surprising to see these accessions positioned in one clade. The second clade consisted of accessions collected from Georgia, Armenia and Iran. The powdery mildew resistant *sylvestris* accession DVIT3351.27, collected from Alaverdi, Armenia, was in this group. These results in conjunction with analyses that included the entire O34-16 group suggest that the two wild *sylvestris* accessions could have acquired powdery mildew resistance from different genetic backgrounds.

Gene diversity indices (Ho, He, F_IS_) for each group are shown in Table 2. The average F_IS_ value for the Species group (0.22) was higher than the other two groups. This group consisted of only 29 accessions as compared to 165 accessions in the O34-16 group and 186 accessions in the TSL group. The average F_IS_ for the O34-16 group was 0.10; expected heterozygosity was higher than observed for all but one of the 34 markers. The average near zero F_IS_ value for the TSL group (Table 2) suggests that they are a panmictic population in Hardy-Weinberg equilibrium. Values for the differentiation index (F_ST_) among the three groups were very low (0.05) (Table 2). These results indicate that there is no clear differentiation among these groups due to the presence of transitional forms that bridge the groups and indicate active gene flow among them, implying that domestication and selection is underway.

### Distinguishing wild and cultivated types by flower phenotype

Because two of the newly discovered powdery mildew resistant accessions were collected as *sylvestris*, it was important to confirm their true type based on morphological traits. Flower sex phenotype and seed morphology are two key criteria used to differentiate subsp. *sylvestris* (dioecious vines, seeds with short beaks) from cultivated *sativa* forms (predominantly hermaphroditic flowers, seeds with larger beaks). The flower phenotype of the subsp. *sylvestris* accessions collected from Armenia, Georgia and Turkmenistan could not be determined because they were young potted plants. Flower phenotype data for 15 wild *V. vinifera* accessions was obtained from GRIN, the National Germplasm Resource Information Network [31]. A combination of two DNA markers was used to differentiate male, hermaphrodite and female flower phenotype for the set of 380 accessions. Field phenotypic observations for the 95 accessions from the Vassal collection matched the flower phenotype predicted by DNA analysis with only one exception – ‘Yhsouh ali’ (2077Mtp1), which was recorded as a female, but DNA analysis indicated it was a hermaphrodite. These test results indicate that the combination of both markers is a reliable system to determine flower phenotype.

DNA marker-based flower phenotyping of the 40 wild forms of *V. vinifera* subsp. *sylvestris* and all ten newly discovered powdery mildew resistant accessions are presented in Table 4; the results for all other accessions are presented in Additional file 11: Table S10. The flower phenotype was undetermined for 11 accessions due to amplification failure with one or both markers. Phenotypic observations differed from genotypic results for only three accessions. Two accessions in the species group, C-166-025 and DVIT1159.3, are recorded as male, but are hermaphrodite based on the DNA analysis. The third anomaly was the cultivar ‘Neeli’ (DVIT2514), which was scored as a hermaphrodite, but is listed as a female plant in the GRIN database.

**Table 4:** **Determination of flower phenotype with a combination of two markers (APT3 and VVIb23) for the *V. vinifera *subsp. *sylvestris *used in this study**

**Accession ID**	**Accession name**	**Sub**	**Country/Source**	**Flower**	**Flower**	**APTa**	**APTb**	**APTc**	**APTd**	**VVIb23**
		**species**	**collection**	**phenotype**	**genotype**					
DVIT3348.17	*sylvestris*	*sylvestris*	Georgia	H	HF	266	336	397	466	282-286
DVIT3349.08	*sylvestris*	"	"	M	MF	266	336	397	466	280-286
DVIT3349.12	*sylvestris*	"	"	H	HF	266	397	466		282-286
DVIT3350.02	*sylvestris*	"	"	H	HF	266	336	397	466	282-286
DVIT3350.25	*sylvestris*	"	"	M	MF	266	336	397	466	278-286
DVIT3357.04	*sylvestris*	"	"	F	FF	266	397			286-286
DVIT3357.30	*sylvestris*	"	"	H	HF	266	397	466		282-286
DVIT3351.23	*sylvestris*	"	Armenia	M	MF or MH	266	336	466		294-302
**DVIT3351.27**	***sylvestris***	**"**	**"**	**M**	**MF or MH**	**336**	**466**			**294-302**
DVIT3353.31	*sylvestris*	"	"	F	FF	266	397			288-299
DVIT3353.43	*sylvestris*	"	"	M	MF	266	336	397	466	290-294
DVIT3355.04	*sylvestris*	"	"	M	MF	266	336	397	466	294-299
DVIT3355.05	*sylvestris*	"	"	H	HF	266	336	397	466	282-294
DVIT3356.33	*sylvestris*	"	"	F	FF	266	336	397		288-299
DVIT3356.38	*sylvestris*	"	"	M	FF	266	336	397	466	294-299
DVIT1799	O30-51	"	Afghanistan	M	MF or MH	266	397	466		303-322
DVIT1800	O30-53	"	"	H	HF	266	397	466		282-322
DVIT1798	O30-44	"	Iran	M	MF or MH	466	466			303-322
DVIT1802	O33-60	"	"	H	HF	266	397			282-288
**DVIT1803**	**O34-16**	**"**	**"**	**F**	**FF**	**266**	**397**			**288-303**
DVIT1805	O34-26	"	"	M	MF or MH	266	336	397	466	288-299
DVIT1804	O34-29	"	"	H	HF	266	397			282-282
DVIT1806	O34-54	"	"	M	MF or MH	266	397	466		299-302
DVIT1807	O34-55	"	"	M	MF or MH	266	336	397	466	288-288
DVIT1808	O35-07	"	"	F	FF	266	336	397		288-299
DVIT1809	O35-11	"	"	M	MF or MH	266	397	466		299-302
DVIT1811	O35-41	"	"	F	FF	266	397			286-299
DVIT1812	O35-47	"	"	M	MF or MH	266	397	466		286-288
DVIT1813	O35-50	"	"	H	HF	266	397	466		282-286
DVIT1816	O35-64	"	"	H	HF	266	466			282-284
DVIT2442.06	Arybata	"	Turkmenistan	M	MF	266	466			288-307
DVIT2442.01	Arybata	"	"	F	FF	266	397			288-288
DVIT2440.01	Ayedere	"	"	M	MF or MH	266	397	466		299-307
DVIT2440.09	Ayedere	"	"	M	MF or MH	266	397	466		288-307
DVIT2447.09	Uzuntakoy	"	"	H	HF	266	336			282-288
DVIT2447.04	Uzuntakoy	"	"	H	HF	266	336	397	466	282-288
DVIT2445.12	Kara kaytak	"	"	F	FF	266	397			288-288
DVIT2446.12	Yuvankala	"	"	M	MF or MH	266	397	466		288-302
DVIT2446.09	Yuvankala	"	"	H	HF	266	397	466		282-282
DVIT2444.19	Kochtemyr	"	"	H	HF	466	466			282-282
**1186Mtp1**	**Chirai obak**	*sativa*	Tajikistan	**H**	**HF**	**266**	**266**			**282-299**
**DVIT0576**	**Husseine**	"	Afghanistan	**F**	**FF**	**266**	**397**			**288-288**
**20008-14 B**	**Kismish vatkana**	"	Uzbekistan	**H**	**HF**	**266**	**397**	**466**		**282-299**
**DVIT2323**	**Karadzhandal**	"	Uzbekistan	**H**	**HF**	**266**	**397**	**466**		**282-299**
**DVIT0431**	**Khalchili**	"	Afghanistan	**H**	**HF**	**266**	**397**	**466**		**282-288**
**ARM Q01-16**	**Late vavilov**	"	Turkmenistan	**H**	**HF**	**266**	**397**	**466**		**282-288**
**2642Mtp2**	**Matrassa**	"	Azerbaijan	**H**	**HF**	**266**	**397**	**466**		**282-288**
**2657Mtp1**	**Soïaki**	"	Uzbekistan	**F**	**FF**	**266**	**266**			**288-307**
**DVIT1126**	**Sochal**	"	USSR	**F**	**FF**	**266**	**397**			**288-299**
**2510Mtp1**	**Vassarga tchernaia**	"	Uzbekistan	**F**	**FF**	**266**	**266**			**288-299**

The APT3 marker is capable of distinguishing female-flowered vines from males or hermaphrodites. The second marker, VVIb23 has a unique allele linked to the hermaphrodism. With the combination of these markers, flower phenotypes could be reliably determined (M - male, H – hermaphorditic, F - female). Flower phenotypes of the 10 powdery mildew resistant subsp. *sativa* are also detailed with these two markers. Powdery mildew resistant genotypes are in bold font.

DNA marker analysis of flower sex in the *V. vinifera* subsp. *sativa* group of cultivars found that 223 were hermaphrodite, 57 were female, and five were identified as male (Additional file 11: Table S10). One of the five males, ‘Kala Kostan’ (DVIT2534) is recorded as a female in GRIN; the flower phenotype could not be verified for the other four genotypically male cultivars.

Eighteen of the 40 *V. vinifera* subsp. *sylvestris* accessions were male, including newly identified powdery mildew resistant accession, DVIT3351.27. Eight accessions were female, including resistant accession O34-16 (Table 4). Fourteen others were hermaphrodite.

Seeds were extracted from ten of the wild *sylvestris* that H.P. Olmo collected from Iran and Afghanistan (Additional file 12: Figure S2). The combined results from seed morphology (when available) and flower sex phenotyping, revealed that 14 accessions designated as *sylvestris* are likely not pure *sylvestris*, but instead hybridized forms of native wild species and cultivated varieties. Interestingly, three accessions from H.P. Olmo’s O series, which are male, based on genotypic analysis, bear fruit (Additional file 12: Figure S2, Table 4). The flower phenotype of the accession O34-26 (DVIT1805) was scored differently in each of three years on GRIN [31]. Similarly, observations of the flower phenotype for O34-55 on GRIN varied from year-to-year between hermaphrodite and female. O35-47, the third genotypically male accession has been recorded as a hermaphrodite [31].

The flower sex of the 12 powdery mildew resistant accessions was also determined: the *sativa* accessions ‘Husseine’ ‘Soïaki’, ‘Sochal’, and ‘Vassarga tchernaia’ are female vines; the other six including ‘Kishmish vatkana’ and ‘Karadzhandal’ are hermaphrodites (Table 4). Two of the new powdery mildew resistant accessions are clearly *V. vinifera* subsp. *sylvestris*. O34-16 is a female vine with obvious wild type seed morphology (Additional file 12: Figure S2). The accession DVIT3351.27 is a male flowered *V. vinifera* subsp. *sylvestris*.

## Discussion

In this study, we exploited available genetic information on the powdery mildew resistance locus *Ren1* to identify additional germplasm that shared a *Ren1-like* local haplotype, and then attempted to clarify the evolution of powdery mildew resistance and its domestication in cultivated *V. vinifera* subsp. *sativa*. Ten new powdery mildew resistant accessions were discovered that possess a *Ren1-like* local haplotype, which was earlier identified in ‘Kishmish vatkana’ and ‘Dzhandzhal kara’ (syn. ‘Karadzhandal’) from Central Asia [1,2]. We discovered that powdery mildew resistance is present in two *V. vinifera* subsp. *sylvestris* accessions, a taxon considered to be the progenitor of the cultivated form *sativa*. Four of the resistant accessions ‘Vassarga tchernaia’, ‘Chirai (obak)’, ‘Late Vavilov’ and ‘Khalchili’ are obscure varieties with few records in the *Vitis* International Variety Catalog [32] or the European *Vitis* database [33]. The first three accessions were obtained from germplasm collections in Uzbekistan, Tajikistan, and Turkmenistan, respectively; Harold P. Olmo collected ‘Khalchili’ from Afghanistan in 1948. The other four resistant *sativa* accessions are better known. ‘Husseine’ was also collected from Afghanistan and it is available worldwide with records in 20 germplasm collections with 61 synonyms. ‘Matrassa’ was acquired from the Azerbaijan collection, and is available in 15 collections with 26 synonyms. ‘Soïaki’ (Uzbekistan) is found in 10 collections with 3 synonyms. ‘Matrassa’ and ‘Soïaki’ are listed by Russian grape breeders as cultivars for high quality table, sparkling and dessert wines [34]. The eighth resistant *sativa* accession ‘Sochal’ is only held at two collection sites in the USA. Plant inventory records indicate that cuttings of ‘Sochal’ were obtained in 1971 from the N. I. Vavilov institute of Plant industry, Leningrad. Eight of the newly identified accessions carrying *Ren1-like* local haplotypes were acquired from five neighboring countries of Central Asia and the Caucasus, all major junctions for trade on the ancient silk route for thousands of years. It is not hard to believe that selected grape germplasm, favored for desirable fruit characteristics, was moved back and forth in the form of seeds and cuttings from one region to another, where they were likely crossed with local varieties in remote isolated valleys and villages in different regions.

In addition to the identification of eight new powdery mildew resistant accessions, this study also gathered information on the genealogical relationships. A likelihood-based method that determines potential parent progeny relationships without any prior knowledge revealed four first-degree relationships. We identified ‘Vassarga tchernaia’ as the female parent of ‘Kishmish vatkana’ and verified ‘Thompson seedless’ as the male parent [2]. ‘Vassarga tchernaia’ and ‘Sochal’ shared a first-degree relationship, sharing at least one allele at 42 markers. Both are female vines with reflexed stamens and seeded fruit. It is difficult to determine the direction of the relationship between ‘Sochal’ and ‘Vassarga tchernaia’. Nevertheless, both of them are female vines, resistant to powdery mildew, and produce seeded fruit. ‘Sochal’, ‘Vassarga tchernaia’ and ‘Kismish vatkana’ are not found in historical collection/breeding records and may have been disregarded due to undesirable fruit attributes, e.g. loose clusters, and small seeded berries, which did not satisfy selection criteria for that particular region. The other two first-degree relationships identified in this study were between ‘Late Vavilov’ and ‘Karadzhandal’, and between ‘Khalchili’ and the powdery mildew susceptible ‘Yarghouti’. All four are hermaphrodites and only ‘Karadzhandal’ is well known with a recorded history [2]. One of the important findings of this study is that four of the new powdery mildew resistant cultivars ‘Chirai obak’, ‘Husseine’, ‘Matrassa’ and ‘Soiaki’ are not directly related to any other accession in this study or in the complete Vassal collection [35]. This implies that the story of powdery mildew resistance in cultivated varieties is complex and what we have revealed in this study may not be the complete picture due to extinction or missing cultivars in our collections. It is likely that a thorough search of germplasm collections in Central Asia would unearth more resistant germplasm.

With the exception of ‘Matrassa’, STRUCTURE placed all seven new subsp. *sativa* powdery mildew resistant cultivars in the TSL group, even though they were collected from different regions of Central Asia. These results suggest that selection and active flow of desirable plant material was common in this region of grape domestication and that multiple breeding efforts were underway to satisfy the local tastes for quality grapes. The TSL group also indicates that breeding efforts were also directed at seedlessness as ‘Thompson seedless’, one of the ancient varieties, was a popular parent for a large number of table grape cultivars [35].

Two of the resistant accessions in this study belong to the subsp. *sylvestris*, which prompts many questions. Are these two accessions truly wild *sylvestris* that have never been cultivated or are they hybrids between wild and cultivated forms? What is the direction of the gene flow for powdery mildew resistance – did these two *sylvestris* accessions acquire resistance from cultivated forms or did the resistance come from wild types to cultivated forms? O34-16 is a female vine with seeded fruit, and seed shape typical of the *sylvestris* type grapes – small round seeds and short beaks. The accession DVIT3351.27 is a male vine. Dioecy is one of the key traits distinguishing the wild *sylvestris* from the cultivated *sativa*. Additionally, the male flower phenotype is only associated with wild *Vitis* species [36]. According to the model of Antcliff [37], flower phenotype is controlled by a single major locus with three alleles: male (M) dominant to hermaphrodite (H), which is dominant to the female (F). In the wild, one should find only male and female vines in the absence of gene flow from hermaphroditic cultivated varieties. In the case of gene flow from cultivated forms to wild types, one does not expect to observe male flower phenotypes in the progeny; we expect to see a 1:1 ratio of hermaphrodite to female vines but no male vines when wild female flower cluster is fertilized with pollen from a cultivated hermaphrodite. In the case of a gene flow from wild types to cultivated forms, where a heterozygous wild male pollinating a cultivated heterozygous hermaphrodite or a cultivated female vine, one would expect to see a 2:1:1 ratio of males, hermaphrodites and females, or 1:1 ratio of male to female, respectively. The *sylvestris* accessions in this study were collected as seeds. Eighteen of them were reported to be males, eight as females and fourteen as hermaphrodites. The occurrence of hermaphrodites in the supposed wild material is proof of gene flow from cultivated to wild germplasm. Gene flow from cultivated grapes to wild *sylvestris* is thought to be common [38,39]. It is important that future work on wild grape germplasm focuses on all morphological attributes of the material under study and not only flower phenotype, as female vines are not uncommon with gene flow from cultivated to wild forms.

We sequenced a 620 bp fragment that included the 143 bp resistance-linked allele of SC8-0071-014 [2]. The sequence of this region for all the resistant accessions matched with the sequences of the two previously described alleles linked tightly to *Ren1* with occasional SNPs between genetically related and unrelated accessions. The occurrence of SNPs between parental and progeny genomic fragments is likely due to the accumulation of somatic mutations over multiple cycles of vegetative propagation. Vegetative or clonal propagation of grapes is an ancient practice used to maintain desirable cultivars. The occurrence of somatic mutations in grapes, some resulting in significant phenotypic differences is well documented [5]. There is no historical record for most of the powdery mildew resistant plant material identified in this study. It is impossible to determine the number of clonal variants of these varieties that may have existed at different times in different regions. The vines sampled for this study are, of necessity, separated by multiple cycles of vegetative propagation from the actual parent vines and the ortet involved in the natural or purposeful crosses that occurred possibly hundreds or thousands of years ago.

Genetic analysis expanded to a 26 cM genomic fragment with six linked SSR markers (Figure 4) revealed that six of the powdery mildew resistant accessions, including the *sylvestris* O34-16 had an allelic profile for this region that matched the two haplotypes known to carry *Ren1*. It is most likely that individuals sharing an identical allelic profile for such a large fragment of DNA, 40% of chromosome 13, also share powdery mildew resistance with the same ancestral lineage, which is further supported by the fact that three of the above six resistant accessions are related to the previously identified accessions that carry *Ren1* co-segregating alleles (Figure 2, Figure 4).

Several key questions arise from this study. Did the group of powdery mildew resistant accessions, now numbering 12 from a variety of regions, acquire resistance after the introduction of North American powdery mildew into the Old World, or was powdery mildew present in Central Asia and China prior to early 1800 thus allowing disease resistance to evolve over a longer time period? This latter possibility seems unlikely given the number of accessions identified in this study carrying unbroken introgression of 26 cM genomic region. This is highly suggestive of recent events of hybridization and introgression. Is it possible that resistance to powdery mildew is also associated with resistance to other pathogens prevalent in Central Asia, and if so, which pathogens? To address these questions, one must first better understand the history of grape domestication in Central Asia, the past and current biodiversity of this region’s grape germplasm, and be able to deduce information from the molecular nature and evolutionary structural organization of disease resistance genes in the *Ren1* locus.

The historical accounts based on archeological digs and observations of natural and cultivated populations of grapevines [8,11-13,18,19] consider that large scale cultivation of *V. vinifera* existed in Transcaucasia about 8,000 – 6,000 BC, spread to North Africa by the end of 5^th^ millennium BC, and during 1^st^ millennium BC, grape culture was established in Europe [19]. These reports state that cultivation of *V. vinifera* was established in Afghanistan and the oases of Central Asia by the fourth century BC, and that its culture reached China in the second century BC. Despite all this information, there are many gaps in our understanding of grape culture in the ancient World. We do not know whether Central Asia, the Near East and China were in contact in the earliest phases of development of grape culture, although there is evidence of wine making in these cultures around 7,000 BC [19]. There is no information available on the influence of Chinese *Vitis* species in the long history of grape domestication, even though China harbors as many as 40 *Vitis* species [40], and some of them are resistant to powdery and downy mildew [4,41]. Given the small number of Chinese accessions sampled in this study set, it is difficult to assess the direct role and genetic contribution of the Chinese species and other indigenous native species in shaping the morphologically diverse wild *sylvestris* populations in Central Asia. Most importantly, historical grape literature is devoid of information on the presence of diseases and pests in Central Asia, they are not noted widely until the early 1800 s. The only disease of grapevine thought to have originated in the region of grape domestication is grapevine fanleaf virus [42,43]. In the distant past, people may have viewed plant diseases very differently due to a lack of detailed understanding, perhaps resulting in the dearth of historical records. The existence of a greater natural biodiversity in ancient times, which helped to regulate undesirable pests and diseases, is also possible. Then the process of domestication, which emphasized clonal propagation and monoculture lead to biological simplicity resulting in more noticeable incidences of disease outbreaks. It is well known that greater genetic diversity confers at least partial resistance to diseases that are specific to certain strains of pathogens. The developing understanding of resistance to grape powdery mildew disease suggests that resistance is often strain specific [44].

Based on seed morphology, leaf characteristics, flower phenotype, and diversity analyses, O34-16 is most likely a true *sylvestris*. Sequence comparison and linkage analysis in this study and organization of underlying genes in previous study [2] provides highly suggestive evidence that powdery mildew resistance represented by the cluster of genes in the *Ren1* locus was introgressed from the wild progenitor. The principal genes of the *Ren1* locus belong to the NBS-LRR family. This group of genes has a clustered organization that enables resistance to evolve in concert with changing pest and disease strains. Previous work by Coleman et al. [2] analyzed the structural organization of NBS-LRR genes in the *Ren1* locus region and concluded that one of the factors that contribute to the chaotic arrangement of genes in this region is intragenic recombination between tandemly arrayed paralogs. The results presented in this study provide clues that a wild progenitor played an active role in the evolution of powdery mildew resistance, which potentially evolved over a long period of sexual reproduction, and was later, bred and selected into cultivated forms. It is also probable that two different accessions of *sylvestris* had different genetic background of resistance, thus providing unique opportunity to expand the gene pool for powdery mildew resistance breeding. The comparative sequence analysis of resistance genes from the two *sylvestris* accessions would be very useful to gain further insight into the evolution of powdery mildew resistance in Central Asia in the absence of the pathogen. It is also possible that powdery mildew disease existed in the Old World for longer than we currently assume and that resistance in wild populations evolved over a longer time period through sexual recombination.

The main function of plant NBS-LRR proteins is to specifically recognizes pathogen effectors and to initiate and control defense response that severely limit pathogen growth [45]. Several studies have determined varied sites of pre-activation and post-activation localization of NB-LRR proteins. Expanded functions beyond pathogen recognition are likely due to evolutionarily flexible NB-LRR interfaces integrated in other cellular machinery as part of their immune surveillance function [46-48]. Additionally, an ancient sub-clade of NB-LRR proteins can 'help' amplify the function of NB-LRR proteins that sense pathogens [49]. We scanned the PN40024 reference genome and found that the Ren1 locus in this mildew susceptible cultivar co-segregates with 11 NB-LRR genes, with the highest identity with CC-NBS-LRR disease resistance genes from soybean (*Rps1-k*) and potato (*R3a*) [2]. Sequence comparison and functional characterization of cloned NB-LRR genes from the resistant accessions we describe and the susceptible reference haplotype are necessary to determine any of the NB-LRR genes in this interval are responsible for the powdery mildew phenotypes we describe here.

## Conclusions

This international collaboration discovered 10 new powdery mildew resistant *V. vinifera* accessions that will prove invaluable to grape breeding programs focused on high quality fruit and strong resistance to powdery mildew. This discovery also forces a reevaluation of grape evolution in its widely considered center of domestication. The results support the notion that mildew resistance was present in wild species and potentially evolved via sexual recombination. The fact that many of the parent progeny relationships discovered here involved crosses of two self-pollinating hermaphroditic parents further supports the existence of grape breeders and their intentional hybridization efforts. Further evidence of breeding activities is documented by the existence of ‘Thompson seedless’ in the parentage of many seedless grapes from Central Asia. We cannot determine the time period when what appears to be very active grape breeding occurred with the fingerprint database created here, but given the lack of a historical record it seems that these breeding efforts may have occurred hundreds or thousands of years ago. The discovery of powdery mildew resistance in Chinese grape species and the possible transport and role of these species in grape breeding along the Asian trade routes is also intriguing. However, with the limited amount of Chinese grape germplasm, we were unable to prove this occurred with the data presented here. Comparative sequencing of multiple lines of Ren1 from different regions and species would greatly aid our understanding of the evolution of powdery mildew resistance. Finally, it is clear that additional collections of Central Asian grape germplasm are needed to fully understand grape evolution, discover additional sources of mildew resistance, and ensure the survival of this historic and valuable resource.

## Methods

### Plant material and DNA extractions

Grapevine accessions from two germplasm collections were selected based on their presumed geographic origins – from the Eastern Mediterranean through Caucasia to the Middle and Far East (Table 1, Additional file 1: Table S1). A total of 559 accessions representing 461 *Vitis vinifera* subsp. *sativa*, 43 *V. vinifera* subsp. *sylvestris*, 38 accessions of 10 Chinese/Central Asia *Vitis* species, 4 accessions of three North American species and 13 interspecific *Vitis* hybrids were analyzed (Table 1). Fresh or dried leaf tissue, or stem cambium tissue was used for DNA extractions. At the University of California, Davis (UCD), samples were processed using a modified CTAB procedure as described by Lodhi et al. [50] with the exclusion of the RNase step. Standard alcohol DNA precipitation were carried out following one chloroform-isoamyl alcohol wash; DNA was dissolved in 1X TE buffer and stored at −20°C for further use.

### SSR amplification and genotyping

A total of 34 markers were used for simple sequence repeat (SSR) analysis (Additional file 4: Table S4). Twenty of these markers have been used to examine germplasm diversity at the INRA Domaine de Vassal collection [17]. Other markers were selected, either due to their linkage to the known powdery mildew resistance loci *Ren1*, *Run1*, *Run2* and *Ren4* from previous studies or because they were used to manage the USDA National Clonal Germplasm Repository collection at Davis [2,30,41,51].

At UCD, genomic DNA amplifications were carried out based on previously described protocols [52]. Amplifications for each primer pair were carried out separately. The PCR amplifications were performed in 10 μl reaction consisting of 10 ng of template DNA, 5 pmoles of each primer, 2.5 mM of each NTP, 1 μl 10x gold PCR buffer (Perkin Elmer), 0.05 unit AmpliTaq Gold DNA polymerase (Perkin Elmer) and 2 mM MgCl_2_ solution. All SSR markers were amplified under the same thermocycler conditions: 10 min at 95°C; 35 cycles of 45 s at 92°C, 45 s at 56°C, 1 min at 72°C; with a final extension of 10 min at 72°C.

Amplified fragments were separated and sized using polyacrylamide sequencing gel electrophoresis (PAGE) or capillary electrophoresis. When PAGE was used, two independent amplifications of each sample at each marker were run on denaturing 5% polyacrylamide gels with a sequencing ladder as a size standard. Fragments were visualized by silver staining with a commercial kit (Promega, Madison, Wisconsin, USA). Scoring for each marker was double checked, and any ambiguous accessions were rerun, or scored as missing data.

In the later part of the study, fragments were separated and sized on an ABI 3130 Genetic Analyzer (Applied Biosystems, Foster City, CA). Products from up to four primers were analyzed in one injection by using different fluorescent labels (6-FAM, HEX, and NED) on different primers and taking into account the expected fragment size. PCR products were added to an 11 μl: 0.2 μl mixture of HD-formamide and GeneScan HD 400 ROX (Applied Biosystems, Foster City, CA) as the internal size standard, respectively. The fragments were denatured for 2 min at 92°C then injected into a 36 cm capillary filled with the polymer POP-7 (Applied Biosystems, Foster City, CA). Fragment sizes were determined and rounded using Genotyper 2.5 software (Applied Biosystems, Foster City, CA). Four to six common *V. vinifera* cultivars were used as an internal control and to ensure allele calls were consistent with samples run on silver stained sequencing gels.

Allele size data for 133 accessions from the INRA Domaine de Vassal germplasm collection (INRA) were generated in France for 20 SSR markers following procedures described by Laucou et al. [17]. Allele sizes were transformed to match the allele sizes from the UCD data set based on known references and samples common to both group’s data sets. Multi-locus accessions from the INRA set were compared to the UCD data set to identify synonymous samples. Unique accessions from the INRA set were analyzed at UCD with an additional 14 markers to increase marker overlap with the UCD data set.

### Identification of powdery mildew resistant accessions

Two SSR markers reported to flank the *Ren1* locus, VMCNg4E10.1 and UDV124, were used to screen the combined datasets for additional resistant accessions [1]. We hypothesized that resistant accessions would have both flanking resistance associated alleles with SSR markers (VMCNg4e10.1 allele 260 and UDV124 allele 216) and powdery mildew resistance in field screens. Three additional markers (SC8_0071_014, sc47_18 and sc47_20) reported to be closely linked to the *Ren1* locus [2] was also tested. Two of them (SC8_0071_014 and sc47_18) gave clean amplifications and were added to the entire data set of unique germplasm.

### Disease evaluation

Powdery mildew resistance evaluations were made on selected accessions in a field nursery trial under unsprayed conditions. These trials were carried out in the summer of 2009 and 2010. Accessions that carry either one or both flanking SSR marker alleles (UDV124 - allele 216; VMCNg4e10.1 - allele 260) linked to the *Ren1* locus were evaluated. Powdery mildew resistant and susceptible accessions from the UCD breeding program, and well known resistant interspecific hybrids and highly susceptible *V. vinifera* cultivars were used as positive and negative controls (Additional file 6: Table S6). A total of 65 accessions were screened in 2009. Five to six replicates of each accession were propagated from hardwood cuttings and planted in three field nursery rows with 30 cm between plants and rows. Powdery mildew symptoms were evaluated based on the extent of infection following the Organisation Internationale de la Vigne et du Vin criteria (OIV 1984) and scored from 0 to 5: 0 (no disease symptoms); 1 (OIV 9) one or two very small spots; 2 (OIV 7) limited patches of powdery mildew infection; 3 (OIV 5) patches of infection wider than 5 cm in diameter; 4 (OIV 3) many powdery mildew infection spots and abundant mycelium growth; and 5 (OIV 1) where leaves and other tissue types were covered with unlimited patches of powdery mildew infection. In 2009, disease evaluations were carried out twice on the same plant during the last week of August and last week of September. Each observation was considered as one replicate for that accession.

A total of 43 accessions including positive and negative controls were screened in 2010 (Additional file 6: Table S6). The majority of these accessions were Chinese or Central Asian species that were difficult to propagate by hardwood cuttings. These accessions were propagated from herbaceous cuttings that were dipped in rooting hormone and rooted under intermittent mist with bottom heat. Rooted cuttings were planted into small plastic pots and once established were planted into the field nursery. Disease symptoms were evaluated twice during the first week of September and first week of October as described above. Four of the wild *V. vinifera* subsp. *sylvestris* accessions with the resistance allele of the closely linked marker SC8-0071-014 were screened in 2012 under the conditions described above. Accessions maintained in the INRA collection that had *Ren1* linked alleles were evaluated for powdery mildew resistance in an unsprayed greenhouse with susceptible controls and artificial inoculum in 2012.

Because powdery mildew disease symptoms were recorded as discrete categories, the ordinal logistic regression model platform of JMP (9.0) (SAS Institute Inc, North Carolina, USA) was used to estimate the effectiveness of the screen by comparing the significance level of genotype, date, field nursery bed and year.

### Sequencing of resistant allele that co-segregates with *Ren1* locus

Two SSR markers (SC8-0071-014 and sc47-18) were reported to co-segregate with *Ren1* locus in ‘Kishmish vatkana’ and ‘Karadzhandal’ [2]. A sequence fragment was obtained using the PN40024 genome [53] by aligning the sequence of SC8-0071-014. Primers were designed around the region of SC8-0071-014 that generated a 625 bp amplification product. PCR products were cloned for 16 accessions using the pGEM®-T Easy vector system using standard protocols. ‘Khwangi’ was the seventeenth accession that had the 143 bp allele, but it was powdery mildew susceptible in the field trial and was not included for sequencing. Eight to twelve positive colonies were selected for each accession and DNA was extracted using the Qiagen plasmid mini kit. PCR amplifications were carried out with the SC8-0071-014 primers [2] in order to identify two alleles of each accession using standard protocols. Sequencing with SP6 primer was carried out only on those samples that represent 143 allele haplotype. Sequences were aligned with the Clustal V method by using the MegAlign application of DNASTAR Lasergene V8.1.

### Genetic diversity and parentage analysis

Accessions with seven or more missing data were not included in the genetic diversity analysis. Next, two different data sets were prepared: the first set consisted of 394 unique accessions that included interspecific hybrids, European reference winegrape varieties and North American species; there were 380 accessions in the second set after the 14 samples of hybrids, North American species and European wine grape varieties were excluded. Simple matching distance (SMD) [54] was calculated with 19 (representing the 19 grape chromosomes) and 34 SSR markers on both data sets. Hierarchical clustering (Ward method) and principal coordinate analysis were carried out with DARWIN V5.0.158 [55] to determine the number of groups.

Following these analyses, STRUCTURE V2.3.1 was used to infer the number of clusters with 19 and 34 markers, and with both data sets of 394 and 380 accessions [56]. The membership of each accession was run for a range of genetic clusters with K values of 1 to 10 using the admixture model, and it was replicated 10 times for each K. Each run was implemented with a burn-in period of 100,000 steps followed by 400,000 Monte Carlo Markov Chain replicates using no prior information and assuming correlated allele frequencies. The posterior probability was then calculated for each value of K using the estimated log-likelihood of K to choose the optimal K [57]. The results from STRUCTURE were displayed by DISTRUCT software [58].

The microsatellite tool kit software [59] was used to calculate standard parameters of genetic variability: expected heterozygosity (He); allele frequencies (AF); and observed heterozygosity (Ho). The deviation from Hardy-Weinberg equilibrium at each locus was examined by calculating the inbreeding coefficient ‘F_IS_’ for each group, and the overall differentiation index ‘F_ST_’ with FSTAT V2.9.3.2 software [60]. The probability of identity (PI), probability of exclusion (PE) and LOD likelihood ratios for potential parent-progeny relationships were calculated with FAMOZ software [61]. The 10,000 simulated pairs were performed to identify a log of the odds ratios (LOD) score threshold to assess a potential parent pair with 34 SSR markers. Only pairs with LOD scores higher than the threshold level were considered. A discrepancy of a maximum of two loci was allowed to cover possible data errors [62], null alleles [63], and clonal mutations as previously described [64]. Potential parental pairs were further evaluated if a discrepancy in the allelic data was observed. They were amplified and repeated either on denaturing polyacrylamide gels or using the ABI 3130 Genetic Analyzer. Additional markers were also added on putative parental pairs.

### Determination of flower phenotype and evaluation of bunch and berry characters

Without prior knowledge of flower phenotype, we utilized a specifically designed marker from gene APT3 (adenine phosphoribosyl transferase) capable of distinguishing female plants from males or hermaphrodites [36; personal communication R. Töpfer, Julius Kuhn-Institut for Grapevine Breeding, Geilweilerhof, Germany]. We also used a specific allele of the SSR marker VVIb23 that is closely linked with the sex locus on chromosome 2, and is capable of distinguishing hermaphrodites from females or male plants. A total of 380 accessions were analyzed that consisted of 40 *V. vinifera* subsp*. sylvestris*, 29 Chinese/Central Asian species, and 311 cultivated varieties including ‘Thompson seedless’ (Table 4, Additional file 11: Table S10).

All fruiting accessions were evaluated for bunch and berry characteristics as per OIV grape descriptor (http://www.oiv.int/) guidelines. Bunch size (without the peduncle), bunch density, berry size, berry shape, berry presence of seeds, berry skin color (without bloom) were recorded. Simple matching distance (SMD) [54] and principal coordinate analysis (PCoA) were carried out on this data using DARWIN software.

## Competing interests

The authors declare that they have no competing interests.

## Authors’ contributions

SR conceived the idea and design of the study, developed fingerprint and sequence database, carried out population, kinship and sequence alignment analysis, assisted in field powdery mildew evaluations, and wrote the manuscript; JMB provided flower phenotype data and participated in drafting the manuscript; GSD assisted in developing finger print profiles and with drafting the manuscript; TL assisted in the interpretation of the genetic diversity, variety identification and population analysis; VL provided the French grape germplasm fingerprint database, contributed to the discussion, assisted in sending the dried leaf samples, and helped with the interpretation of FAMOZ results; ACT assisted in gathering germplasm, carrying out disease evaluations and provided feedback for the manuscript; MAW was involved in conceiving and designing the study, discussions on results and interpretations and oversaw the final draft and revisions. All authors have read and approved the final manuscript.

## Supplementary Material

Additional file 1: Table S1 List of the 559 accessions and control reference varieties analyzed in the study. Country of origin codes are according to the ISO 3166–1 alpha 3 standards. Fifty-five genotypes with accession identification starting with "Turkmn" were introduced from the 'Turkmenian Experimental Station of Plant Genetic Resources, Garrygala, Turkmenistan' in 1996. Dr. Nolsulchak acquired ten accessions of *V. vinifera* subsp. *sylvestris* from Turkmenistan as seeds gathered from the Koptdag mountain range in 1993. Click here for file

Additional file 2: Table S2 List of accessions that were potentially identical between the two collections based on fingerprint profiles from 10 SSR markers, but with different names. Accession IDs and names in blue font are maintained in the Vassal germplasm repository, and all others are maintained at Davis, California. Unique SSR profiles of accessions in bold are presented in Table S3. Three accessions highlighted in grey matched to ‘Houssein blanc’ (0Mtp484) in the Vassal collection; the genotypic profile for this group is missing in Table S3. Click here for file

Additional file 3: Table S3 SSR marker allelic data for 403 unique accessions with 19 SSR markers. Missing data are indicated with a hyphen. Click here for file

Additional file 4: Table S4 List of SSR markers and allele size ranges. Click here for file

Additional file 5: Table S5 SSR allele data with four markers linked to the *Ren1* locus. Alleles associated with powdery mildew resistance are in bold. Two previously identified and 10 newly identified powdery mildew resistant accessions are underlined. Missing data are indicated with a hyphen. Click here for file

Additional file 6: Table S6 List of accessions screened for powdery mildew resistance in a no-spray field nursery trial. Bold and italicized accessions were included as known resistant and susceptible controls. Powdery mildew symptoms were recorded on leaves and canes. Plants in group A were screened in 2009; plants in group B were screened in year 2010; plants in group C were screened in 2012. Group D accessions are maintained in the Vassal collection (INRA, France) – they were evaluated under unsprayed greenhouse evaluations. Click here for file

Additional file 7: Table S7 Observed probability of identity [P(Id)], probability of exclusion of a single parent and parent pair, and cumulated power of exclusion of a single parent and parent pair calculated from 394 unique accessions using FAMOZ on 34 SSR markers. Click here for file

Additional file 8: Table S8 SSR marker profiles for 13 accessions with 34 markers. Eight additional markers were added to six accessions to verify parent progeny relationships. Missing data are noted with hyphens. Click here for file

Additional file 9: Table S9 The estimated coefficients of membership proportions values (Q-values) for the three ancestral genetic clusters inferred with STRUCTURE. Click here for file

Additional file 10: Figure S1 Dendrogram of 40 *V. vinifera* subsp. *sylvestris* accessions based on hierarchal cluster analysis (Ward method). Two accessions in red font are *V. vinifera* subsp. *sylvestris* that were resistant to powdery mildew in field trials. Click here for file

Additional file 11: Table S10 Determination of flower phenotype and genotype. APT3 gene marker distinguished females (F) from males (M) or hermaphrodites (H). SSR marker VVIb23 is tightly linked to the flower sex locus and the unique allele 282 is linked to hermaphrodism. With the combination of these two markers, the sex phenotype of a grapevine could be determined without prior knowledge. All bold and italicized flower phenotypes for samples from the Vassal collection were verified with field data. Click here for file

Additional file 12: Figure S2 Seeds of ten accessions from the Olmo-series *V. vinifera* subsp. *sylvestris*; the last two samples have seeds with a fragile cap on small beaks. Click here for file
