# Supplementary material for: Identification of mildew resistance in wild and cultivated Central Asian grape germplasm
Source: BMC Plant Biol. 2013 Oct 4;13:149. doi: 10.1186/1471-2229-13-149 (PMC3851849; doi:10.1186/1471-2229-13-149)
Supplement: Additional file 1: Table S1 — List of the 559 accessions and control reference varieties analyzed in the study. Country of origin codes are according to the ISO 3166–1 alpha 3 standards. Fifty-five genotypes with accession identification starting with "Turkmn" were introduced from the 'Turkmenian Experimental Station of Plant Genetic Resources, Garrygala, Turkmenistan' in 1996. Dr. Nolsulchak acquired ten accessions of V. vinifera subsp. sylvestris from Turkmenistan as seeds gathered from the Koptdag mountain range in 1993. [file 1471-2229-13-149-S1.pdf]

Supplementary Table S1. List of the 559 accessions and control reference varieties analyzed in the study. Country of origin codes are according to the ISO 3166-1 alpha 3 standards. Fifty-five genotypes with accession identification starting with "Turkmn" were introduced from the 'Turkmenian Experimental Station of Plant Genetic Resources, Garrygala, Turkmenistan' in 1996. Dr. Nolsulchak acquired ten accessions of *V. vinifera* subsp. *sylvestris* from Turkmenistan as seeds gathered from the Koptdag mountain range in 1993.

Accession ID	Repository	Accession Name	Prime Variety Name	Country of origin/ Source collection	Species Davis / Vassal
	Davis-Control1	Chardonnay	Chardonnay	ITA	*V. vinifera*
	Davis-Control2	Riesling	Riesling	DEU	"
	Davis-Control3	Thompson Seedless	Sultanine	FRA	"
	Davis-Control4	Zinfandel	Primitivo	FRA	"
	Davis-Control5	Pinot noir		FRA	"
	Davis-Control6	Cabernet Sauvignon		FRA	"
199Mtp1	Vassal-Control1	Chardonnay	Chardonnay	ITA	"
274Mtp5	Vassal-Control2	Riesling	Riesling	GRC	"
1566Mtp2	Vassal-Control3	Sultanie ou Belabesses	Sultanine	FRA	"
1277Mtp6	Vassal-Control4	Zinfandel	Primitivo	FRA	"
2076Mtp1	Vassal	A Kalatchel	A Kalatchel	IRN	"
1979-0-2219-S1	Davis	A'asemi S1		YEM	"
DVIT0333	Davis	Ab Jusht	Ab Jusht	AFG	"
1748Mtp1	Vassal	Abdjouch	Abjouch	"	"

DVIT0305	Davis	Abla Aganin Isium	Abla Aganyn Isyum (A. A. Isium)	USSR	"
DVIT0327	Davis	Abla Aganin Isium	Abla Aganyn Isyum (A. A. Isium)	"	"
TYR VI 10-01	Davis	Abla Aganin Isium		Unknown	"
TYR VI 10-03	Davis	Afuz-Ali Urmasti		Unknown	"
2842Mtp1	Vassal	Ag Kiourdach p.e.	Ag Kiourdach faux	AZE	"
TYR VI 10-05	Davis	Agaday		Unknown	"
DVIT0334	Davis	Agadia	Agadai (Agadia)	USSR	"
TYR VI 10-07	Davis	Agadia		Unknown	"
2982Mtp1	Vassal	Ahmeh Sal	Ahmeh Sal apyrène	IRN	"
DVIT2037	Davis	Aitah	Aita (Aitah)	AFG	"
DVIT0336	Davis	Aitah Ghulaman	Aitah Ghulaman	"	"
DVIT0563	Davis	Ajmi	Ajmi	IRQ	"
DVIT3154	Davis	Ajmi	Ajmi	Unknown	"
2897Mtp1	Vassal	Ak ouzioum tapapskii	Ak ouzioum tagapskii	RUS	"
DVIT0612	Davis	Ak Schekerek	Ak Shekerek	TKM	"
2898Mtp1	Vassal	Ak schekerek	Ak chekerek	"	"
Turkmn547	Davis	Ak Shekerek		"	"
DVIT0307	Davis	Al Borla	al Borla	USSR	"
DVIT0634	Davis	Alburla	Albourla (Alburla)	"	"

Turkmn19652	Davis	Ali Shaitan		TKM	"
2858Mtp1	Vassal	Alia boka	Alia boka	UZB	"
DVIT0337	Davis	Alloued Zeine	Alloued Zeine	LBN	"
1183Mtp1	Vassal	Alü tierskü	Alii tierskii	RUS	"
DVIT0338	Davis	Alulu	Alulu	IRQ	"
0Mtp27	Vassal	Anab E Shabi	Anab-e-Shabi	IND	"
TYR VI 10-09	Davis	Anab-E-Shaki	Anab-E-Shahi	Unknown	"
2113Mtp1	Vassal	Aragatzi	Aragatzi	ARM	"
2001-9-8093-01	Davis	Asgari 01		IRN	"
DVIT0343	Davis	Askari	Askari	AFG	"
DVIT3157	Davis	Askari	Askari	Unknown	"
2088Mtp1	Vassal	Askari	Askari noir	IRN	"
DVIT0344	Davis	Askari Khari	Askari Khari	AFG	"
DVIT0565	Davis	Askary	Askary	IRN	"
2083Mtp1	Vassal	Askudi	Askudi	"	"
DVIT0308	Davis	Asma	Asma	USSR	"
DVIT2054	Davis	Asma	Asma	"	"
DVIT0309	Davis	Asma Sohwars	Asma Sohwars	"	"
2505Mtp1	Vassal	Assylkara	Assyl kara	RUS	"

DVIT2085	Davis	Aswad		YEM	"
DVIT2087	Davis	Aswad	Aswad	"	"
DVIT2171	Davis	B 15-19		Unknown	"
DVIT2306	Davis	Baharat Early		IND	"
2271Mtp2	Vassal	Baïan chirei	Bayan Shirei	AZE	"
DVIT0346	Davis	Baidh Ul Haman	Baidh ul Haman	Unknown	"
HOP VD19-01	Davis	Barbara		USA	"
DVIT2499	Davis	Bargoon	Bargoon	PAK	"
DVIT0310	Davis	Barmak Isium	Barmak Isium	TUR	"
156Mtp1	Vassal	Baxtiori	Baxtiori	UZB	"
1749Mtp1	Vassal	Bayad	Bayad	YEM	"
DVIT1841	Davis	Beli Potok		Yugoslavia	"
DVIT2501	Davis	Berurargoon	Berurargoon	PAK	"
1981-0-2233-S1	Davis	Bez el Anza S1		EGY	"
TYR VI 10-21	Davis	Bharat Early		Unknown	"
DVIT0311	Davis	Bias Kukuzeti	Bias Kukuzeti	USSR	"
2001-9-8095-01	Davis	Bidaneh 01		Unknown	"
2087Mtp1	Vassal	Bidaneh Ghelmez	Askéri	IRN	"
DVIT0358	Davis	Black Kishmish	Kishmish Chernyi (Black Kishmish)	USSR	"

DVIT1326	Davis	Black Kishmish	Kishmish Chernyi (Black Kishmish)	"	"
DVIT2055	Davis	Black Kishmish	Black Kishmish	RUS	"
1830Mtp1	Vassal	Black Monucca	Black Monukka	USA	"
DVIT0312	Davis	Blanc De Crimei	Blanc De Crimee (Blanc de Crimei)	USSR	"
2507Mtp1	Vassal	Bouaki nor	Bouaki nor	UZB	"
2509Mtp1	Vassal	Boulany	Bulanyi	RUS	"
DVIT2496	Davis	Boyalsing I	Boyalsing I	"	"
DVIT2505	Davis	Boyalsing II	Boyalsing II	"	"
DVIT0673	Davis	Buaki		RUS	"
DVIT2506	Davis	Budrilay	Budrilay	PAK	"
DVIT2512	Davis	Buraburi	Buraburi	"	"
TYR VI 11-11	Davis	Caus		Yugoslavia	"
TYR VI 11-13	Davis	Caus X Pearl Csaba		"	"
DVIT2308	Davis	Caus X Perle Of Csaba		"	"
2675Mtp1	Vassal	Chaani blanc	Chaani biely	AZE	"
2676Mtp1	Vassal	Chaani noir	Chaani tchernyi	"	"
2677Mtp2	Vassal	Chakour angour	Chakar angour de Tachkent	UZB	"
DVIT2730	Davis	Chamo	Chamo	PAK	"
DVIT0613	Davis	Chan Isium	Chan Isium	USSR	"

DVIT0371	Davis	Chaouch	Chaouch blanc (Chaouch)	TUR	"
DVIT0685	Davis	Charas	Charas	USSR	"
TYR VI 12-05	Davis	Charli Sar		IND	"
0Mtp225	Vassal	Chassany	Chassany	AZE	"
DVIT1088	Davis	Chaush White	Chaush White	TUR	"
TYR VI 12-07	Davis	Chiradzouli White		USSR	"
1186Mtp1	Vassal	Chirai (oback)	Chirai obak	TJK	"
2678Mtp2	Vassal	Chirvan chakki	Chirvan chakhi	AZE	"
Turkmn16392	Davis	Chol Uzyum		TKM	"
DVIT2532	Davis	Churgoon	Churgoon	PAK	"
DVIT0313	Davis	Cirmisi Sap De Sudak	Cirmisi Sap de Sudak	USSR	"
DVIT0384	Davis	Coudsi	Dabouki (coudsi)	Unknown	"
DVIT0388	Davis	Dabouki	Dabouki	ISR	"
DVIT1982	Davis	Dabouki	Dabouki	"	"
DVIT0569	Davis	Dais-el-anz	Dais-el-anz	IRQ	"
DVIT2509	Davis	Dalnato I	Dalnato I	PAK	"
DVIT2536	Davis	Dardari	Dardari	"	"
635Mtp1	Vassal	Dattier noir	Hunisa	IRN	"
857Mtp1	Vassal	De Hengril	Kizliarsky	RUS	"

DVIT0314	Davis	Demir Kara	Demir Kara	USSR	"
Turkmn13358	Davis	DK #01		TKM	"
Turkmn13359	Davis	DK #02		"	"
Turkmn13360	Davis	DK #03		"	"
Turkmn13361	Davis	DK #04		"	"
Turkmn13362	Davis	DK #05		"	"
Turkmn13364	Davis	DK #07		"	"
Turkmn13365	Davis	DK #08		"	"
Turkmn13366	Davis	DK #09		"	"
Turkmn13388	Davis	DK #10		"	"
Turkmn13387	Davis	DK #11		"	"
Turkmn13386	Davis	DK #12		"	"
Turkmn13381	Davis	DK #17		"	"
Turkmn13389	Davis	DK #2		"	"
Turkmn13379	Davis	DK #21 Chernyi		"	"
Turkmn13377	Davis	DK #39		"	"
Turkmn6272	Davis	DK #9		"	"
Turkmn6977	Davis	DK Belyi		"	"
Turkmn13375	Davis	DK Melkii Chernyi		"	"

Turkmn13374	Davis	DK Melkii Krasnyi		"	"
Turkmn30743	Davis	DK N15 (#15)		"	"
Turkmn6979	Davis	DK S Opushonnym Listom		"	"
0Mtp318	Vassal	Doppelaugen	Doppel Augen	AZE	"
DVIT0315	Davis	Dschanim Isium		USSR	"
Turkmn6218	Davis	Ekdona Turkmenskaya		TKM	"
0Mtp354	Vassal	Fachren blanc	Fachren blanc	AZE	"
DVIT0316	Davis	Fachren Weis		USSR	"
DVIT0402	Davis	Fahri	Fahri Kalamak (Fahri)	AFG	"
DVIT0403	Davis	Fahri Kalamak	Fahri Kalamak	"	"
DVIT2510	Davis	Fatai	Fatai	PAK	"
DVIT2636	Davis	Fayoumi	Fayoumi	EGY	"
0000-0-4432-S1	Davis	Fetyaska S1		RUS	"
DVIT0615	Davis	Ganjehi	Ganjehi	IRN	"
DVIT2537	Davis	Gaschochi	Gaschochi	PAK	"
Turkmn3028	Davis	Gechi Kyrlen		TKM	"
0Mtp414	Vassal	Geok souli	Kizil sapak	"	"
DVIT0409	Davis	Ghula Dani	Ghula Dari	AFG	"
DVIT2039	Davis	Ghula Dari	Ghula Dari	"	"

0Mtp428	Vassal	Golodan	Golodan	"	"
0Mtp429	Vassal	Gora Chirine n°1 (par erreur)	Gora Chirine faux	IRN	"
DVIT0413	Davis	Gros Colman	Gros Colman	USSR	"
DVIT3164	Davis	Gros Colman	Gros Colman	RUS	"
DVIT2500	Davis	Gungargoon	Gungargoon	PAK	"
Turkmn3033	Davis	Gurgon		TKM	"
TYR VI 13-09	Davis	Guzal Kara		UZB	"
0Mtp468	Vassal	Guzal Kara	Guzal Kara	"	"
0Mtp471	Vassal	Haïtha	Haita safid	AFG	"
Turkmn6971	Davis	Halili Nobat Niyaz		TKM	"
Turkmn6994	Davis	Han Uzyum		"	"
DVIT0416	Davis	Hassaine	Hassaine	Unknown	"
DVIT2638	Davis	Hassaine	Hassaine	Unknown	"
DVIT0417	Davis	Henab	Henab	TUR	"
DVIT0328	Davis	Himrisnky	Himrisnky	USSR	"
DVIT2175	Davis	Himrisnky	Himrisnky	"	"
DVIT0759	Davis	Hisakasy		RUS	"
Turkmn19806	Davis	Hiv Uzyum		TKM	"
DVIT2503	Davis	Hosargoon	Hosargoon	PAK	"

0Mtp484	Vassal	Houssein blanc (par erreur)	Houssein rouge	IRN	"
TYR VI 13-15	Davis	Huseine Rozvoj		Unknown	"
DVIT0419	Davis	Husseine	Husseine	AFG	"
DVIT0420	Davis	Husseine	Husseine	"	"
DVIT0576	Davis	Husseine	Husseine	"	"
DVIT0421	Davis	Husseive	Husseine (Rish Baba)	"	"
DVIT2050	Davis	Husseive	Husseine	"	"
2086Mtp1	Vassal	Inconnu blanc	Blanc d'Iran (Charif)	IRN	"
1752Mtp1	Vassal	Irki ou Ergi	Irki	YEM	"
Turkmn3026	Davis	Irtyk Yaprak		TKM	"
DVIT2508	Davis	Ishkin	Ishkin	PAK	"
DVIT2677	Davis	Itchkimar	Itchkimar	RUS	"
2845Mtp1	Vassal	Itchkimar biely	Itchkimar biely faux (Collection Kichinev)	UZB	"
1221Mtp2	Vassal	Itshkimar	Ichkimar	"	"
TYR VI 13-17	Davis	Jane De Smirna		Unknown	"
TYR VI 13-19	Davis	Jefferson Persian R27		Unknown	"
2679Mtp1	Vassal	Joumalak blanc	Yumalak lelyi	UZB	"
DVIT0766	Davis	Kabajink	Kabajink	Unknown	"
DVIT2497	Davis	Kabuli	Kabuli	PAK	"

DVIT0317	Davis	Kakourdess Weis	Kakourdess Biely	USSR	"
DVIT2534	Davis	Kala Kostan	Kala Kostan	PAK	"
DVIT0335	Davis	Kalamak	Kalamak Safid	AFG	"
DVIT2917	Davis	Kali Sag		Unknown	"
TYR VI 13-21	Davis	Kali Sag		Unknown	"
657Mtp1	Vassal	Kalily	Khalili belyi	IRN	"
2663Mtp2	Vassal	Kalily noir	Khalili tcherni	"	"
DVIT0428	Davis	Kandahar	Kandhari Kara	Unknown	"
HOP L06-23	Davis	Kandahar		Unknown	"
2908Mtp1	Vassal	Kandahar	Kandahar	USA	"
DVIT0429	Davis	Kandahari	Kandahari Siah	AFG	"
1746Mtp1	Vassal	Kandari noir	Kandahari siah	"	"
DVIT2918	Davis	Kandhar		Unknown	"
TYR VI 13-23	Davis	Kandhar		Unknown	"
DVIT2081	Davis	Kandhari		IND	"
TYR VI 14-01	Davis	Kandhari		Unknown	"
0Mtp565	Vassal	Kanfet isium	Kanfet isioum	RUS	"
TYR VI 14-03	Davis	Kara Djandjal		USSR	"
2277Mtp1	Vassal	Kara Djandjal	Djandjal kara	UZB	"

DVIT2322	Davis	Kara Dzhidzhigi	Kara Dzhidzhigi	"	"
Turkmn6255	Davis	Kara Dzhidzhigi		TKM	"
TYR VI 14-05	Davis	Kara Dzhidzhigi		Unknown	"
2690Mtp1	Vassal	Kara Kaltak	Kara Kaltak	UZB	"
DVIT0773	Davis	Kara Lakana	Kara Lakana	USSR	"
2276Mtp1	Vassal	Kara ouzume d'Aschkhabadsky	Kara ouzume Aschkhabadsky	TKM	"
2780Mtp1	Vassal	Kara Palvan	Kara Palvan	UZB	"
Turkmn6982	Davis	Kara Terbash		TKM	"
Turkmn551	Davis	Kara Uzyum Ashhabadskii		"	"
Turkmn3036	Davis	Kara Uzyum Nuhurskii		"	"
DVIT2703	Davis	Karabournov	Karabournov	Unknown	"
DVIT2323	Davis	Karadzhandal	Karadzhandal	USSR	"
TYR VI 14-07	Davis	Karazhumdal		Unknown	"
Turkmn21551	Davis	Karga Dili		TKM	"
Turkmn6981	Davis	Kash Uzyum		"	"
DVIT2451	Davis	Kashiri	Kashiri	PAK	"
DVIT2103	Davis	Kastour Rumi	Kastour Rumi	EGY	"
1738Mtp1	Vassal	Katta	Kata	AFG	"
556Mtp2	Vassal	Katta kourgan	Katta-kourgan	UZB	"

TYR VI 14-09	Davis	Katta Kourgane		Unknown	"
DVIT0774	Davis	Katta Kurgan	Katta Kourgan (Katta Kurgan)	USSR	"
2951Mtp1	Vassal	Kaytagi	Kaitagi	RUS	"
1679Mtp2	Vassal	Kechmisch ali violet	Kechmisch aly violet	IRN	"
DVIT0580	Davis	Keshmesh		IRN	"
Turkmn29892	Davis	Keshmesh Heshrau		TKM	"
ARM Q01-20	Davis	Keshmesh Zeravshan		"	"
DVIT0430	Davis	Khalchili	Khalchili	AFG	"
DVIT0431	Davis	Khalchili	Khalchili	"	"
DVIT0432	Davis	Khaldar	Khaldar	"	"
DVIT0433	Davis	Khalili		"	"
DVIT2040	Davis	Khalili		"	"
DVIT2084	Davis	Khalili	Khalili	"	"
0Mtp1473	Vassal	Khan isium	Khan ousioum femelle	TKM	"
2190Mtp1	Vassal	Khatmi	Khatmi	RUS	"
DVIT2919	Davis	Khawngi		IND	"
TYR VI 14-11	Davis	Khawngi		Unknown	"
2664Mtp1	Vassal	Khindogny	Khindogny	IRN	"
DVIT0434	Davis	Khir Ghulaman	Khir Ghulaman	AFG	"

DVIT2605	Davis	Khorestini	Khorestini	PAK	"
1227Mtp2	Vassal	Khoussaïné blanc	Khoussaïné blanc	UZB	"
DVIT2075	Davis	Khusaine Red	Khusaine Red	USSR	"
PI 349737	Davis	Khusaine White		UZB	"
2781Mtp1	Vassal	Kibraïski	Kibraïski	"	"
0Mtp1491	Vassal	Kichmich noir	Kichmich tcherni	TUR	"
DVIT2495	Davis	Kini Yatch	Kini Yatch	PAK	"
1678Mtp5	Vassal	Kischmisch	Kichmich rond	TUR	"
DVIT0435	Davis	Kishmish	Kishmishi	AFG	"
TYR VI 14-15	Davis	Kishmish Charni		Unknown	"
DVIT2325	Davis	Kishmish Charni	Kishmish-charni	IND	"
DVIT2324	Davis	Kishmish Early	Kishmish Early	UZB	"
TYR VI 14-17	Davis	Kishmish Early		Unknown	"
DVIT1103	Davis	Kishmish Hishrau	Kishmish Khishrau	UZB	"
Turkmn6999	Davis	Kishmish Krasnyi Turkmenskii		TKM	"
DVIT2071	Davis	Kishmish Of Vir	Kishmish (of) Vir	USSR	"
DVIT2074	Davis	Kishmish Of Vir	Kishmish (of) Vir	RUS	"
DVIT0437	Davis	Kishmish Sorkh	Kishmish Sorkh	AFG	"
DVIT2041	Davis	Kishmish Sorkh	Kishmish Sorh (Kishmish Sorkh)	"	"

DVIT0438	Davis	Kishmish Spien	Kishmish Spien	"	"
DVIT0436	Davis	Kishmishi	Kishmishi	"	"
DVIT0439	Davis	Kishmishi		"	"
TYR VI 14-19	Davis	Kishmishi		Unknown	"
0Mtp587	Vassal	Kishmishi siogag	Sahebi	AFG	"
0Mtp589	Vassal	Kisil izium	Kizil ousum faux	RUS	"
746Mtp1	Vassal	Kisil sapak	Kizil sapak faux	"	"
20008-14 B	Davis	Kismish Vatkana		UZB	"
Turkmn19697	Davis	Kismish Turkmenskii		TKM	"
Turkmn545	Davis	Kizil Sapak		"	"
DVIT2077	Davis	Klusaine Red	Khusaine Red	USSR	"
2001-9-8100-01	Davis	Kondori 01		IRN	"
DVIT0783	Davis	Koptcha	Kopchak (Koptcha)	RUS	"
0Mtp610	Vassal	Korisa kechmich	Korza erevani	ARM	"
DVIT2680	Davis	Kouldjinski	Kouldjinski	RUS	"
DVIT0329	Davis	Kovalewka	Kovalevka	USSR	"
2635Mtp1	Vassal	Koz ouzioum	Koz ouzioum	RUS	"
DVIT1070	Davis	Kule Dary	Kule Dary	Unknown	"
TYR VI 14-21	Davis	Kulidzhinski		Yugoslavia	"

Turkmn18820	Davis	Kush Dzhumurtka		TKM	"
DVIT2452	Davis	Kwar II	Kwar II	PAK	"
DVIT0441	Davis	Lal	Lal	AFG	"
DVIT0442	Davis	Lal Sorkh	Lal Sorh	"	"
ARM Q01-16	Davis	Late Vavilov		TKM	"
Turkmn29890	Davis	Late Vavilov		"	"
DVIT2641	Davis	Leanoy	Leanoy	USSR	"
0Mtp1475	Vassal	Liali bidona	Vardabouïre	AZE	"
0Mtp640	Vassal	Liali Iakdona	Liali Yakdona	KAZ	"
2640Mtp1	Vassal	Lkeni noir	Lkeny chernyi	AZE	"
0Mtp1449	Vassal	Malahy	Malahy	IRN	"
Turkmn3025	Davis	Mamidon		TKM	"
Turkmn6984	Davis	Mamidon Deli		"	"
Turkmn21604	Davis	Mamidon Kizil		"	"
0Mtp703	Vassal	Matrassa	Matrassa blanc faux	RUS	"
2642Mtp2	Vassal	Matrassa	Matrassa	AZE	"
2001-9-8101-01	Davis	Mehdi 01		IRN	"
2082Mtp1	Vassal	Mehdik	Mehdik	"	"
Turkmn3030	Davis	Mellei		TKM	"

DVIT1042	Davis	Mermark	Mermark	IRQ	"
Turkmn6217	Davis	Meshei		TKM	"
0Mtp1795	Vassal	Mesisti rose	Mesisti	RUS	"
DVIT2498	Davis	Millishun	Millishun	PAK	"
1742Mtp1	Vassal	Monaca	Monaca	AFG	"
DVIT0460	Davis	Monakka	Monakka	"	"
DVIT0462	Davis	Monukka		"	"
0Mtp750	Vassal	Mouchketny	Mouchketny faux	RUS	"
DVIT0319	Davis	Mourvedre Famellestadt	Mourvèdre Famellestadt	USSR	"
DVIT2046	Davis	Munuka Sufed	Munuka Sufed	AFG	"
DVIT0320	Davis	Mursa Isium	Murza Isyum (Murma Isium)	USSR	"
TYR VI 15-13	Davis	Mzivani		Unknown	"
DVIT2511	Davis	Namonia	Namonia	PAK	"
1741Mtp1	Vassal	Naosé	Naosé	AFG	"
2648Mtp2	Vassal	Narma	Narma	RUS	"
0Mtp780	Vassal	Nassau	Nassau	"	"
DVIT2514	Davis	Neeli	Neeli	PAK	"
DVIT2507	Davis	Neelilay	Neelilay	"	"
Turkmn30746	Davis	Neriiski		TKM	"

Turkmn13368	Davis	Neriiskii		"	"
DVIT2504	Davis	Nilok	Nilok	PAK	"
TYR VI 15-17	Davis	Nimrang		RUS	"
TYR VI 15-19	Davis	Nimrang		"	"
DVIT0476	Davis	Nimrang #58	Nimrang (Nimrang #58)	USSR	"
2736Mtp1	Vassal	Nimrang rouge	Nimrang rouge	UZB	"
DVIT0330	Davis	Noir D'automne	Noir d' Automne (Noir D'automne)	USSR	"
DVIT2683	Davis	Norakert PRG 2224	Norakert PRG 2224	RUS	"
DVIT2533	Davis	Nosargoon	Nosargoon	PAK	"
2649Mtp1	Vassal	Noulizok	Noulizok	UZB	"
DVIT2045	Davis	Nunaka Sia	Nunaka Sia	AFG	"
2650Mtp1	Vassal	Obak blanc	Obak biely	UZB	"
0Mtp828	Vassal	Oeil de Dragon	Long Yan	CHN	"
2854Mtp1	Vassal	Oktiabrskii	Oktiabrskii	UZB	"
Turkmn30748	Davis	Orion		TKM	"
2952Mtp1	Vassal	Otscha bala	Otcha bala	UZB	"
2075Mtp1	Vassal	Ozaan Daii	Ozaan Daii	IRN	"
DVIT2282	Davis	Pakistan Collection 25168		PAK	"
DVIT2269	Davis	Pakistan Collection 25180		"	"

DVIT2270	Davis	Pakistan Collection 25193		"	"
DVIT2262	Davis	Pakistan Collection 25206		"	"
DVIT2273	Davis	Pakistan Collection 25226		"	"
DVIT2261	Davis	Pakistan Collection 25227		"	"
DVIT2267	Davis	Pakistan Collection 25234		"	"
DVIT2272	Davis	Pakistan Collection 25237		"	"
DVIT2271	Davis	Pakistan Collection 25241		"	"
DVIT2281	Davis	Pakistan Collection 25246		"	"
DVIT2268	Davis	Pakistan Collection 25258		"	"
DVIT2293	Davis	Pakistan Collection 25265		"	"
DVIT2263	Davis	Pakistan Collection 25275		"	"
DVIT2266	Davis	Pakistan Collection 25281		"	"
DVIT2296	Davis	Pakistan Collection 25290		"	"
DVIT2283	Davis	Pakistan Collection 25296		"	"
DVIT2291	Davis	Pakistan Collection 25305		"	"
DVIT2264	Davis	Pakistan Collection 25311		"	"
DVIT2502	Davis	Parargoon		"	"
2691Mtp1	Vassal	Parkentskii	Parkent	UZB	"
2597Mtp1	Vassal	Peikani	Peikani	IRN	"

0Mtp865	Vassal	Pejnery blanc	Peïneri	IRN	"
TYR VI 16-03	Davis	Persian		Unknown	"
DVIT2755	Davis	Persian R27		Unknown	"
2651Mtp2	Vassal	Pervenetz Praskoveisky	Pervenetz praskoveïsky	RUS	"
2788Mtp1	Vassal	Pin el pou tao	Pinger putao	CHN	"
Turkmn19735	Davis	Porsi Shekerek		TKM	"
DVIT0321	Davis	Precoce d'Astrachan		USSR	"
0Mtp928	Vassal	Précoce d'Astrakan	Bekalny	RUS	"
0Mtp950	Vassal	Rajulan	Rajoulan	UZB	"
DVIT2684	Davis	Ranny Vira		RUS	"
2654Mtp1	Vassal	Ranny Vira	Rannii Vira	"	"
2737Mtp1	Vassal	Razakiia piembiana	Razaki pembe	"	"
1750Mtp1	Vassal	Raziki ou Razaki	Raziki	YEM	"
DVIT0499	Davis	Red Ohanez		RUS	"
TYR VI 16-09	Davis	Rhazaki (Pa 1882)		Unknown	"
TYR VI 16-11	Davis	Rhazaki (Pb 1886) (Dattier?)		Unknown	"
TYR VI 16-13	Davis	Rhazaki (Pg 1887)		Unknown	"
DVIT0608	Davis	Rhazaki Anatolico		GRC	"
DVIT0554	Davis	Rhazaki Arhanon		Unknown	"

DVIT0501	Davis	Rhazaki De Crete		GRC	"
DVIT0555	Davis	Rhazaki Mavro		Unknown	"
HOP L04-19	Davis	Rish Baba		Unknown	"
DVIT2338	Davis	Rizamat		UZB	"
2783Mtp1	Vassal	Rouchaki	Rouchaki	USSR	"
DVIT0507	Davis	Rozovii Kishmish		"	"
DVIT3077	Davis	Sabalkanskoi	Dabouki (Sabalkanskoi)	Unknown	"
0Mtp1007	Vassal	Sabs angur	Sabza angur	TJK	"
2090Mtp1	Vassal	Sahami	Sahami	IRN	"
DVIT0508	Davis	Sahebi		AFG	"
DVIT0509	Davis	Sahibi		"	"
DVIT0510	Davis	Sahibi Sorkh		"	"
2078Mtp1	Vassal	Sahilii	Sahilii	IRN	"
0Mtp1010	Vassal	Saïd guliami	Saïd guliami	UZB	"
DVIT0511	Davis	Saidi-Gulami		USSR	"
Turkmn6987	Davis	Sary Aygyr		TKM	"
0Mtp1031	Vassal	Sary Kiriak	Sary Kirak	AZE	"
388Mtp2	Vassal	Sateni tcherni	Sateni tcherny	ARM	"
1737Mtp1	Vassal	Schiradzouli violet	Schiradzouli violet	IRN	"

DVIT2604	Davis	Schwin		PAK	"
DVIT1152	Davis	Selection 2798		Yugoslavia	"
DVIT2653	Davis	Sereksiya Rosavi		RUS	"
DVIT0331	Davis	Setzlinge Dodreliabi	Gros Colman (Setzlinge Dodreliabi)	USSR	"
2001-9-7097-01	Davis	Shahani 01		IRN	"
TYR VI 16-21	Davis	Shakar Angur		USSR	"
DVIT0520	Davis	Shando Khani	Shando Khari	AFG	"
DVIT0521	Davis	Shindu Khani	Shindu Khari	"	"
DVIT2603	Davis	Shingargoon		PAK	"
2079Mtp1	Vassal	Shirazi	Shirazi	IRN	"
DVIT2168	Davis	Shtur Angur		USSR	"
DVIT2513	Davis	Shuwarti		PAK	"
2074Mtp1	Vassal	Siah	Siah	IRN	"
DVIT0522	Davis	Siar		AFG	"
2681Mtp1	Vassal	Siborskory	Sibirkovy	RUS	"
DVIT1126	Davis	Sochal		USSR	"
DVIT2340	Davis	Sochal Kara-kash		"	"
2657Mtp1	Vassal	Soïaki	Soïaki	UZB	"
DVIT0597	Davis	Solaimani	Salomani	IRQ	"

0Mtp1071	Vassal	Soultani (avec graine)	Soultani (Collection Kichinev)	UZB	"
2856Mtp1	Vassal	Sourkhak bely	Sourkhak biely	"	"
DVIT2602	Davis	Spin Ugad		PAK	"
DVIT0332	Davis	Stambulari	Stambulari (Stambul Ali)	USSR	"
0Mtp1073	Vassal	Starinky	Starinky	RUS	"
2857Mtp1	Vassal	Stour Angour	Chtour angour	UZB	"
DVIT0526	Davis	Sultana		RUS	"
DVIT1321	Davis	Sultani	Sultana	USSR	"
DVIT0530	Davis	Sultanina Rose		RUS	"
0Mtp1597	Vassal	Sultanine noire	Sultanine noire faux	UZB	"
Turkmn6304	Davis	Sumbarskii Bekyi		TKM	"
2659Mtp1	Vassal	Tagobi	Tagobi	TJK	"
DVIT2174	Davis	Taifi		USSR	"
TYR VI 16-23	Davis	Taifi		Unknown	"
1192Mtp1	Vassal	Taïfi rose	Taïfi rosovy	UZB	"
TYR VI 17-01	Davis	Taifi Rosmovyi		USSR	"
TYR VI 17-03	Davis	Taka Sago		JPN	"
2661Mtp2	Vassal	Tana kouzy	Tana kouzy	UZB	"
DVIT2928	Davis	Tana-Kuzi		Unknown	"

TYR VI 17-05	Davis	Tana-Kuzi		USSR	"
TYR VI 17-07	Davis	Tarnau		"	"
TYR VI 17-09	Davis	Tasch Buaky		Unknown	"
1218Mtp1	Vassal	Tavkveri	Tavkveri	AZE	"
DVIT2688	Davis	Tchilar		RUS	"
2671Mtp1	Vassal	Tchiliaki blanc	Tchiliaki belyi	TJK	"
2221Mtp1	Vassal	Terbach	Terbasch	TKM	"
Turkmn549	Davis	Terbash		"	"
1985Mtp2	Vassal	Tolstokory	Tolstokorii	RUS	"
DVIT2531	Davis	Torgoon		PAK	"
0000-0-2411-S1	Davis	Trapanlarin kara S1		USSR	"
0Mtp1148	Vassal	Tscharma	Tscharma	UZB	"
664Mtp1	Vassal	Tschetirbak	Tchatyrbac	"	"
DVIT0539	Davis	Tschilaci		RUS	"
1985-0-2415-S1	Davis	Tufachi S1		ISR	"
2760Mtp1	Vassal	Tuia tiche	Tuia-tiche	UZB	"
654Mtp1	Vassal	Tuia-tiche p.e.	Kok pandas	RUS	"
TYR VI 17-11	Davis	Uzbekistan Muscat		Unknown	"
TYR VI 17-12	Davis	Uzbekistan Muscat		Unknown	"

DVIT2072	Davis	Uzbekistanian Muscat		USSR	"
0Mtp1165	Vassal	Varuschkin	Varyoshkin	RUS	"
126Mtp2	Vassal	Vassarga blanc	Vassarga bielaia	UZB	"
2510Mtp1	Vassal	Vassarga tchernaia	Vassarga tchernaia	"	"
TYR VI 17-15	Davis	Vitis Vinifera #1359		"	"
1983-0-2424-S1	Davis	Voivoginiova S1		USSR	"
TYR VI 17-17	Davis	Volgo Don		UZB	"
1736Mtp5	Vassal	Weisse Schirastraube	Schiradzouli = Tebrizi	IRN	"
DVIT0604	Davis	Yaghotti No. 1		"	"
DVIT2052	Davis	Yaghotti No. 2		"	"
2687Mtp1	Vassal	Yaï izium rose	Yaï isioum rosovy	RUS	"
TYR VI 17-19	Davis	Yarghouti		Unknown	"
2077Mtp1	Vassal	Yhsouh Ali	Yhsouh ali	IRN	"
0Mtp1798	Vassal	Zarevoï	Zorevoi	RUS	"
DVIT0606	Davis	Zerk		IRQ	"
2666Mtp1	Vassal	Zimliansky noir	Tzimlansky chernyi	RUS	"
DVIT2664	Davis	Zimsko Belo		Yugoslavia	"
TYR VI 17-21	Davis	Zimsko Belo		Unknown	"
DVIT2442.1	Davis	Arybata		TKM	*V. sylvestris*

DVIT2442.6	Davis	Arybata	"	"
DVIT2440.1	Davis	Ayedere	"	"
DVIT2440.9	Davis	Ayedere	"	"
DVIT2445.12	Davis	Kara Kaytak	"	"
DVIT2444.19	Davis	Kochtemyr	"	"
DVIT2447.4	Davis	Uzuntakoy	"	"
DVIT2447.9	Davis	Uzuntakoy	"	"
DVIT2446.9	Davis	Yuvankala	"	"
DVIT2446.12	Davis	Yuvankala	"	"
DVIT1799	Davis	O30-51	AFG	"
DVIT1800	Davis	O30-53	"	"
DVIT1798	Davis	O30-44	IRN	"
DVIT1801	Davis	O33-50	"	"
DVIT1802	Davis	O33-60	"	"
DVIT1803	Davis	O34-16	"	"
DVIT1804	Davis	O34-29	"	"
DVIT1805	Davis	O34-46	"	"
DVIT1806	Davis	O34-54	"	"
DVIT1807	Davis	O34-55	"	"

DVIT1808	Davis	O35-07	"	"
DVIT1809	Davis	O35-11	"	"
DVIT1811	Davis	O35-41	"	"
DVIT1812	Davis	O35-47	"	"
DVIT1813	Davis	O35-50	"	"
DVIT1814	Davis	O35-58	"	"
DVIT1816	Davis	O35-64	"	"
DVIT3355.4	Davis	*V. sylvestris*	ARM	"
DVIT3355.5	Davis	*V. sylvestris*	"	"
DVIT3353.31	Davis	*V. sylvestris*	"	"
DVIT3353.43	Davis	*V. sylvestris*	"	"
DVIT3351.23	Davis	*V. sylvestris*	"	"
DVIT3351.27	Davis	*V. sylvestris*	"	"
DVIT3356.38	Davis	*V. sylvestris*	"	"
DVIT3356.33	Davis	*V. sylvestris*	"	"
DVIT3350.25	Davis	*V. sylvestris*	GEO	"
DVIT3350.2	Davis	*V. sylvestris*	"	"
DVIT3348.14	Davis	*V. sylvestris*	"	"
DVIT3348.17	Davis	*V. sylvestris*	"	"

DVIT3357.30	Davis	*V. sylvestris*	"	"
DVIT3357.4	Davis	*V. sylvestris*	"	"
DVIT3349.12	Davis	*V. sylvestris*	"	"
DVIT3349.8	Davis	*V. sylvestris*	"	"
545685	Geneva. NY	545685	Unknown	*Ampelopsis delavayana*
GVIT 859	Geneva. NY	597257.03	CHN	*V. piasezkii*
588421	Geneva. NY	588421.a	"	*V. yenshanensis*
588422	Geneva. NY	588422.a	"	*V. yenshanensis*
GVIT 813	Geneva, NY	588451.c	USSR	*V. coignetiae*
GVIT0814	Geneva, NY	588452.b	"	*V. amurensis*
588650	Geneva. NY	588650.a	CHN	*V. yenshanensis*
597294.01	Geneva. NY	A-166-003	"	*Vitis* species
DVIT1158.4	Davis	*amurensis*	"	*V. amurensis*
DVIT1157.12	Davis	*amurensis*	"	*V. amurensis*
DVIT1158.1	Davis	*amurensis*	"	*V. amurensis*
DVIT2006.1	Davis	*amurensis*	KOR	*V. amurensis*
DVIT1156.2	Davis	*amurensis*	CHN	*V. amurensis*
DVIT1157.2	Davis	*amurensis*	"	*V. amurensis*
DVIT2005.5	Davis	*amurensis*	"	*V. amurensis*

597298.01	Geneva. NY	B-166-016	"	*Vitis* species
588715.01	Geneva. NY	B-166-019	"	*Vitis* species
DVIT1432	Davis	Beaumont	USA	*V. riparia*
DVIT2596.1	Davis	*betulifolia*	CHN	*V. betulifolia*
597296.01	Geneva, NY	C-166-025	"	*V. romanetii*
DVIT2550	Davis	C-166-026	"	*V. romanetii*
597297.01	Geneva, NY	C-166-039	"	*V. romanetii*
DVIT3192	Davis	C-166-043	"	*V. romanetii*
DVIT1159.10	Davis	*coignetiae*	Unknown	*V. coignetiae*
DVIT1159.3	Davis	*coignetiae*	Unknown	*V. coignetiae*
DVIT1159.9	Davis	*coignetiae*	Unknown	*V. coignetiae*
DVIT2008.5	Davis	*ficifolia*	KOR	*V. ficifolia*
DVIT2008.7	Davis	*ficifolia*	"	*V. ficifolia*
DVIT1160.2	Davis	*ficifolia*	CHN	*V. ficifolia*
DVIT1160.7	Davis	*ficifolia*	"	*V. ficifolia*
DVIT1385	Davis	*flexuosa*	Unknown	*V. flexousa*
597295.01	Geneva. NY	J-167-048	CHN	*Vitis* species
DVIT2349.13	Davis	*jacquemontii*	PAK	*V. jacquemontii*
DVIT2350.17	Davis	*jacquemontii*	"	*V. jacquemontii*

DVIT2354.7	Davis	*jacquemontii*		"	*V. jacquemontii*
DVIT2355.11	Davis	*jacquemontii*		"	*V. jacquemontii*
DVIT2539	Davis	Kali Dakh II		"	*V. jacquemontii*
DVIT1815	Davis	O35-59		"	*V. lanata*
DVIT2027	Davis	*piasezkii*		Unknown, Asia	*V. piasezkii*
DVIT2032	Davis	*piasezkii*		Unknown, Asia	*V. piasezkii*
DVIT1772	Davis	Thomas		USA	*M. rotundifolia*
DVIT1756	Davis	Trayshed		USA	*M. rotundifolia*
DVIT3009	Davis	Aurore (S. 5279)		FRA	Interspecific hybrids
0000-0-289-04	Davis	Baco blanc (Baco 22A)		FRA	"
C 67-00	Davis	Chambourcin (JS 26.205)		FRA	"
GVIT0342	Davis	Couderc 13		FRA	"
DVIT0257	Davis	De Chaunac (Seibel 9549)		FRA	"
DVIT0196	Davis	Jaeger 70		USA	"
6039Mtp1	Vassal	Joannès Seyve 23416	Joannès Seyve 23416	FRA	"
DVIT0197	Davis	JS23-416		FRA	"
DVIT0214	Davis	Kuhlmann 188-2	Kuhlmann 188-2 (Marechal Foch)	FRA	"
DVIT2685	Davis	Sev Lernatu PRG 2224		RUS	"
DVIT2685	Davis	Sev Lernatu PRG 2224		RUS	"

HOP W11-13	Davis	SV 12.375 Villard blanc	FRA	"
DVIT2741	Davis	Vignoles	FRA	"
