# Supplementary material for: Identification of mildew resistance in wild and cultivated Central Asian grape germplasm
Source: BMC Plant Biol. 2013 Oct 4;13:149. doi: 10.1186/1471-2229-13-149 (PMC3851849; doi:10.1186/1471-2229-13-149)
Supplement: Additional file 2: Table S2 — List of accessions that were potentially identical between the two collections based on fingerprint profiles from 10 SSR markers, but with different names. Accession IDs and names in blue font are maintained in the Vassal germplasm repository, and all others are maintained at Davis, California. Unique SSR profiles of accessions in bold are presented in Table S3. Three accessions highlighted in grey matched to ‘Houssein blanc’ (0Mtp484) in the Vassal collection; the genotypic profile for this group is missing in Table S3. [file 1471-2229-13-149-S2.pdf]

Supplementary Table S2. List of accessions that were potentially identical between the two collections based on fingerprint profiles from 10 SSR markers, but with different names. Accession IDs and names in blue font are maintained in the Vassal germplasm repository, and all others are maintained at Davis, California. Unique SSR profiles of accessions in bold are presented in Table S3. Three accessions highlighted in grey had similar fingerprint profiles to 'Houssein blanc' (0Mtp484) in the Vassal collection; the genotypic profile for this group is missing in Table S3.

Accession ID	Accession Name
**DVIT0634**	**Alburla**
TYR VI 10-01	Abla Aganin Isium
DVIT0327	Abla Aganin Isium
DVIT0332	Stambulari
1606Mtp1	Albourlah
**TYR VI 10-03**	**Afuz-Ali Urmasti**
DVIT2703	Karabournov
TYR VI 16-13	Rhazaki (Pg 1887)
DVIT0554	Rhazaki Arhanon
634Mtp6	Rhazaki
**TYR VI 10-05**	**Agaday**
2189Mtp1	Agadaï
**DVIT0338**	**Alulu**
1662Mtp3	Rosa menna di vacca
**2001-9-8093-01**	**Asgari 01**
DVIT3157	Askari
DVIT0344	Askari Khari
DVIT0565	Askary
2087Mtp1	Bidaneh Ghelmez
**DVIT0308**	**Asma**
DVIT0309	Asma Sohwars
DVIT2054	Asma
0Mtp811	Noir de Crimée p.e.
**DVIT2306**	**Baharat Early**
TYR VI 10-21	Bharat Early
570Mtp11	Orange muscat

**DVIT0310**	**Barmak Isium**
1736Mtp5	Weisse Schirastraube
**DVIT0311**	**Bias Kukuzeti**
DVIT0317	Kakourdess Weis
734Mtp1	Mischung
**DVIT0312**	**Blanc De Crimei**
2249Mtp1	Gordin
**DVIT0673**	**Buaki**
585Mtp58	Chasselas 1
**DVIT0613**	**Chan Isium**
0Mtp1473	Khan isium
**DVIT0371**	**Chaouch**
DVIT1088	Chaush White
TYR VI 12-07	Chiradzouli White
1673Mtp8	Chaouch de Bulgarie
**DVIT0685**	**Charas**
2460Mtp2	Negru mare
**DVIT0315**	**Dschan Im Isium**
0Mtp819	Nuju isium
0000-0-4432-S1	Fetyaska S1
1661Mtp1	Leanyka
**DVIT0432**	**Khaldar**
DVIT0409	Ghula Dari
1738Mtp1	Katta
**DVIT0417**	**Henab**
DVIT2103	Kastour Rumi
1677Mtp1	Hénab
**DVIT0328**	**Himrisnky**
DVIT2175	Himrisnky
9Mtp7	Graciano
**DVIT0759**	**Hisakasy**
2512Mtp2	Mardgenni

**TYR VI 13-15**	**Huseine Rozvoj**
0Mtp484	Houssein blanc
DVIT2075	Khusaine Red
DVIT2074	Kishmish Of Vir
DVIT2077	Klusaine Red
0Mtp484	Houssein blanc
**Turkmn 3026**	**Irtyk Yaprak**
0Mtp865	Pejnery blanc
**DVIT0196**	**Jaeger 70**
5854Mtp3	Maréchal Joffre
**TYR VI 13-17**	**Jane De Smirna**
433Mtp1	Darkaïa de Jérusalem
**DVIT0576**	**Husseine**
DVIT0429	Kandahari
DVIT0539	Tschilaci
1746Mtp1	Kandari noir
**DVIT2322**	**Kara Dzhidzhigi**
Turkmn 6255	Kara Dzhidzhigi
TYR VI 14-05	Kara Dzhidzhigi
0Mtp1793	Karadjidji
**Turkmn 551**	**Kara Uzyum Ashhabadskii**
2276Mtp1	Kara ouzume d'Aschkhabadsky
**DVIT2323**	**Karadzhandal**
TYR VI 14-03	Kara Djandjal
TYR VI 14-07	Karazhumdal
2277Mtp1	Kara Djandjal
**Turkmn 29892**	**Keshmesh Heshrau**
ARM Q01-20	Keshmesh Zeravshan
DVIT1103	Kishmish Hishrau
2773Mtp1	Kichmich chichraou
**DVIT0430**	**Halili beliij**
657Mtp1	Kalily
**TYR VI 17-19**	**Yarghouti**

DVIT2052	Yaghotti No. 2
DVIT2324	Kishmish Early
TYR VI 14-17	Kishmish Early
Turkmn 6999	Kishmish Krasnyi Turkmenskii
1741Mtp1	Naosé
**DVIT0435**	**Kishmishi**
DVIT0436	Kishmishi
1221Mtp2	Itshkimar
**DVIT1070**	**Kule Dary**
Turkmn 549	Terbash
2221Mtp1	Terbach
**DVIT2641**	**Leanoy**
0Mtp639	Lesnoy
**2001-9-8101-01**	**Mehdi 01**
2082Mtp1	Mehdik
**DVIT0462**	**Monukka**
1830Mtp1	Black Monucca
**DVIT0319**	**Mourvedre Famellestadt**
64Mtp2	Mourvèdre
**TYR VI 15-13**	**Mzivani**
1676Mtp2	Mtzvané
**TYR VI 15-17**	**Nimrang**
TYR VI 15-19	Nimrang
0Mtp29	Angoor Kalan
**DVIT0330**	**Noir D'automne**
0Mtp809	Noir d'Automne
**DVIT0321**	**Precoce D'astrachan**
0Mtp87	Bekalny
**DVIT0499**	**Red Ohanez**
DVIT3077	Sabalkanskoi
1731Mtp1	Sabalkanskoï
**DVIT0608**	**Rhazaki Anatolico**

1247Mtp1	Baresan
**DVIT0555**	**Rhazaki Mavro**
0Mtp1935	Inconnu noir glabre (Archanes)
**HOP L04-19**	**Rish Baba**
1227Mtp2	Khoussaïné blanc
**DVIT2168**	**Shtur Angur**
DVIT2653	Sereksiya Rosavi
3007Mtp1	Babeasca gris
**DVIT2174**	**Taifi**
TYR VI 16-23	Taifi
TYR VI 17-01	Taifi Rosmovyi
1192Mtp1	Taïfi rose
**DVIT2928**	**Tana-Kuzi**
TYR VI 17-05	Tana-Kuzi
2661Mtp2	Tana kouzy
**TYR VI 17-07**	**Tarnau**
2841Mtp2	Turnau
**TYR VI 17-11**	**Uzbekistan Muscat**
316Mtp1	Verdelho de Madère
**DVIT2664**	**Zimsko Belo**
TYR VI 17-21	Zimsko Belo
2709Mtp1	Zimsko belo
