# Supplementary material for: Identification of mildew resistance in wild and cultivated Central Asian grape germplasm
Source: BMC Plant Biol. 2013 Oct 4;13:149. doi: 10.1186/1471-2229-13-149 (PMC3851849; doi:10.1186/1471-2229-13-149)
Supplement: Additional file 3: Table S3 — SSR marker allelic data for 403 unique accessions with 19 SSR markers. Missing data are indicated with a hyphen. [file 1471-2229-13-149-S3.pdf]

**Supplementary Table S3.** SSR marker allelic data for 403 unique accessions with 19 SSR markers. Missing data are indicated with a hyphen.

| Variety name and ID | VVIp60 | VVIb01 | VVMD28 | VVMD32 | VVMD27 | VVMD21 | VVMD7 | VMC1b11 | VVIq52 | VVIv37 | VVS02 | VMC4f3.1 | VVIh54 | VVMD24 | VVIv67 | VVMD5 | VVIn73 | VVIn16 | VVIp31 |
|---|---|---|---|---|---|---|---|---|---|---|---|---|---|---|---|---|---|---|---|
| Zinfandel (Reference) | 315-319 | 290-298 | 247-257 | 255-263 | 179-181 | 241-247 | 247-249 | 173-181 | 83-83 | 155-157 | 133-143 | 172-178 | 167-177 | 206-206 | 353-360 | 226-236 | 256-263 | 149-151 | 174-186 |
| Riesling (Reference) | 303-321 | 288-298 | 227-233 | 251-271 | 181-189 | 247-247 | 249-257 | 165-183 | 77-83 | 157-157 | 143-151 | 172-188 | 149-163 | 206-214 | 353-360 | 226-234 | 263-263 | 149-151 | 174-182 |
| Chardonnay (Reference) | 315-319 | 288-294 | 217-227 | 239-271 | 181-189 | 247-247 | 239-243 | 165-183 | 77-83 | 149-159 | 137-143 | 172-178 | 163-167 | 206-214 | 360-368 | 234-238 | 263-263 | 149-149 | 178-182 |
| Thompson Seedless (Reference) | 315-317 | 290-298 | 217-243 | 249-249 | 181-194 | 247-254 | 239-253 | 165-183 | 79-83 | 155-177 | 145-151 | 188-190 | 163-165 | 206-215 | 353-353 | 234-234 | 261-263 | 151-155 | 178-182 |
| A Kalatchel (2076Mtp1) | 319-319 | 290-306 | 243-257 | 249-251 | 181-194 | 241-247 | 247-249 | 165-165 | 83-83 | 149-149 | 125-151 | 188-188 | 165-167 | 206-218 | 353-363 | 234-234 | 256-263 | 149-155 | 184-188 |
| A'asemi S1 (1979-0-2219-S1) | 315-319 | 290-290 | 247-257 | 249-255 | 194-194 | 247-247 | 239-251 | 165-181 | 77-79 | 149-171 | 145-151 | 164-188 | 159-177 | 206-206 | 357-366 | 232-240 | 263-263 | 147-149 | 184-188 |
| Ab Jusht (DVIT2040) | 315-317 | 290-294 | 243-257 | 249-261 | 179-194 | 247-254 | 247-247 | 165-193 | 79-83 | 159-159 | 151-151 | 172-182 | 147-165 | 206-210 | 353-361 | 234-240 | 256-267 | 151-151 | 184-186 |
| Abjouch (1748Mtp1) | 319-319 | 290-290 | 243-243 | 257-271 | 179-194 | 241-254 | 247-247 | 165-165 | 77-79 | 157-177 | 151-151 | 188-188 | 165-165 | 206-210 | 353-375 | 234-240 | 263-263 | 151-151 | 184-190 |
| Abla Aganin Isium (DVIT0305) | 315-325 | 290-294 | 235-257 | 257-261 | 179-189 | 247-254 | 239-239 | 165-181 | 77-83 | 155-159 | 143-145 | 166-206 | 165-165 | 206-206 | 353-360 | 240-246 | 263-263 | 149-149 | 182-194 |
| Afuz-Ali Urmasti (TYR VI 10-03) | 315-315 | 290-294 | 233-257 | 257-271 | 185-185 | 254-254 | 239-249 | 183-183 | 77-79 | 147-159 | 133-135 | 166-188 | 165-177 | 206-206 | -:- | 226-232 | 263-263 | 149-149 | 172-182 |
| Ag Kiourdach p.e. (2842Mtp1) | 315-315 | 290-294 | 243-257 | 243-271 | 194-194 | 246-247 | 239-249 | 165-173 | -:- | 149-149 | 145-145 | 166-174 | 159-165 | 206-210 | 353-357 | 234-236 | 256-265 | 149-151 | 172-194 |
| Agaday (TYR VI 10-05) | 315-319 | 290-294 | 235-257 | 249-271 | 185-185 | 247-249 | 249-249 | 173-183 | 77-83 | 155-173 | 135-151 | 174-202 | 151-165 | 206-210 | 353-353 | 228-234 | 256-263 | 151-151 | 188-190 |
| Ahmeh Sal apyrène (2982Mtp1) | 315-317 | 298-306 | 217-235 | 249-251 | 181-194 | 241-247 | 239-249 | 165-165 | 79-83 | 159-177 | 135-151 | 186-188 | 163-175 | 206-206 | 371-371 | 234-246 | 256-261 | 147-155 | 182-186 |

| | | | | | | | | | | | | | | | | | | | |
|---|---|---|---|---|---|---|---|---|---|---|---|---|---|---|---|---|---|---|---|
| Ajmi (DVIT0563) | **323-327** | **290-290** | **243-257** | **255-273** | **185-194** | **247-247** | **239-249** | **177-187** | **79-83** | **149-149** | **143-153** | **182-202** | **139-139** | **204-215** | **355-355** | **240-246** | **263-263** | **-:-** | **172-186** |
| Ak ouzioum tapapskii (2897Mtp1) | **299-328** | **290-306** | **237-243** | **249-251** | **179-194** | **241-247** | **243-249** | **165-165** | **77-77** | **155-167** | **143-153** | **164-172** | **165-165** | **210-214** | **353-357** | **228-228** | **256-263** | **147-151** | **174-186** |
| Ak Schekerek (DVIT0612) | **315-315** | **290-306** | **243-257** | **249-273** | **179-185** | **246-246** | **239-253** | **165-165** | **79-83** | **159-159** | **135-141** | **174-188** | **165-165** | **206-206** | **353-353** | **236-240** | **256-256** | **149-151** | **184-190** |
| Al Borla (DVIT0307) | **317-319** | **294-294** | **247-257** | **249-261** | **183-187** | **246-254** | **239-239** | **165-171** | **79-81** | **155-173** | **143-145** | **168-206** | **151-167** | **206-206** | **353-377** | **228-238** | **263-267** | **149-151** | **176-188** |
| Alburla (DVIT0634) | **319-321** | **294-294** | **251-257** | **251-251** | **179-194** | **241-247** | **249-249** | **177-183** | **77-79** | **155-159** | **145-145** | **174-188** | **165-167** | **206-224** | **351-371** | **240-240** | **256-263** | **151-151** | **174-182** |
| Ali Shaitan (Turkmn 19652) | **-:-** | **290-294** | **234-247** | **257-271** | **179-179** | **241-254** | **235-243** | **165-183** | **79-83** | **159-159** | **133-133** | **172-188** | **147-151** | **206-214** | **353-360** | **240-240** | **256-263** | **149-151** | **184-188** |
| Alia boka (2858Mtp1) | **319-319** | **290-298** | **243-245** | **257-271** | **179-194** | **247-247** | **233-247** | **165-193** | **79-83** | **173-177** | **141-155** | **172-182** | **139-177** | **214-215** | **353-360** | **238-240** | **263-267** | **147-151** | **178-186** |
| Alloued Zeine (DVIT0337) | **315-315** | **290-294** | **243-257** | **249-271** | **179-181** | **241-247** | **239-243** | **169-169** | **77-81** | **149-159** | **149-151** | **186-202** | **167-177** | **206-210** | **353-353** | **224-246** | **263-263** | **147-149** | **178-188** |
| Alü tierskü (1183Mtp1) | **-:-** | **290-294** | **-:-** | **249-251** | **189-192** | **-:-** | **239-245** | **165-183** | **77-79** | **157-159** | **135-147** | **166-178** | **163-165** | **208-210** | **353-371** | **232-240** | **263-265** | **151-151** | **178-180** |
| Alulu (DVIT0338) | **323-323** | **290-294** | **235-257** | **251-271** | **181-185** | **241-254** | **239-247** | **183-187** | **79-79** | **159-167** | **139-143** | **166-172** | **165-177** | **206-210** | **357-357** | **232-246** | **263-263** | **147-149** | **178-182** |
| Anab-e-Shabi (0Mtp27) | **315-319** | **290-294** | **257-257** | **255-271** | **179-185** | **256-264** | **239-243** | **169-173** | **83-83** | **149-149** | **155-155** | **178-178** | **139-139** | **206-206** | **360-366** | **236-238** | **263-263** | **151-151** | **184-184** |
| Anab-E-Shaki (TYR VI 10-09) | **315-319** | **-:-** | **257-257** | **255-271** | **179-185** | **256-264** | **239-243** | **169-173** | **83-83** | **149-149** | **151-151** | **178-178** | **139-139** | **206-206** | **360-366** | **236-236** | **263-263** | **151-151** | **184-184** |
| Aragatzi (2113Mtp1) | **315-319** | **290-290** | **243-243** | **249-271** | **181-194** | **247-254** | **239-243** | **165-183** | **79-79** | **149-177** | **151-155** | **182-188** | **139-163** | **214-215** | **360-360** | **234-234** | **263-267** | **151-155** | **178-184** |
| Asgari 01 (2001-9-8093-01) | **315-317** | **290-298** | **217-257** | **249-271** | **179-194** | **247-254** | **239-253** | **165-183** | **77-83** | **167-177** | **141-145** | **182-188** | **165-177** | **210-215** | **353-397** | **234-238** | **263-263** | **147-155** | **182-182** |
| Askari (DVIT0343) | **315-315** | **290-294** | **233-243** | **271-271** | **185-185** | **247-254** | **249-249** | **165-183** | **79-81** | **159-167** | **133-135** | **166-172** | **165-177** | **206-210** | **353-387** | **226-238** | **263-263** | **149-149** | **172-178** |
| Askari noir (2088Mtp1) | **305-319** | **294-306** | **235-247** | **251-261** | **179-185** | **241-247** | **239-247** | **165-169** | **77-77** | **159-161** | **135-141** | **166-178** | **151-165** | **214-215** | **353-371** | **226-246** | **256-263** | **149-149** | **184-194** |

| | | | | | | | | | | | | | | | | | | | |
|---|---|---|---|---|---|---|---|---|---|---|---|---|---|---|---|---|---|---|---|
| Askudi (2083Mtp1) | **315-317** | **294-298** | **217-257** | **249-271** | **179-194** | **241-247** | **247-253** | **165-171** | **75-83** | **159-177** | **143-151** | **178-188** | **165-167** | **206-206** | **353-357** | **234-234** | **261-263** | **151-155** | **182-190** |
| Asma (DVIT0308) | **315-319** | **290-294** | **257-257** | **251-255** | **185-185** | **241-254** | **243-249** | **173-183** | **77-83** | **157-177** | **135-137** | **178-188** | **165-175** | **215-215** | **353-353** | **226-236** | **263-263** | **149-151** | **176-178** |
| Assylkara (2505Mtp1) | **315-317** | **290-298** | **243-274** | **251-255** | **192-194** | **247-247** | **239-245** | **165-183** | **79-83** | **157-177** | **135-145** | **178-186** | **165-167** | **210-214** | **371-373** | **228-232** | **256-263** | **147-151** | **172-180** |
| Aswad (DVIT2085) | **319-323** | **290-294** | **243-243** | **247-255** | **179-185** | **241-264** | **233-249** | **165-187** | **77-81** | **147-173** | **135-151** | **182-202** | **159-165** | **206-210** | **353-371** | **226-238** | **263-263** | **149-151** | **178-188** |
| B 15-19 (DVIT2171) | **-:-** | **-:-** | **217-247** | **249-261** | **194-194** | **-:-** | **233-253** | **165-183** | **83-83** | **157-177** | **137-145** | **185-188** | **163-165** | **206-206** | **357-357** | **234-234** | **263-263** | **147-155** | **180-184** |
| Baharat Early (DVIT2306) | **315-319** | **294-294** | **245-267** | **239-271** | **179-185** | **247-264** | **247-249** | **173-187** | **77-79** | **149-159** | **133-133** | **166-172** | **163-165** | **206-210** | **358-358** | **228-236** | **261-263** | **147-157** | **182-192** |
| Baïan chirei (2271Mtp2) | **315-319** | **290-294** | **235-243** | **271-271** | **193-194** | **241-247** | **243-247** | **165-171** | **75-79** | **149-155** | **143-143** | **174-186** | **163-165** | **206-206** | **353-368** | **228-238** | **263-263** | **151-155** | **178-188** |
| Baidh Ul Haman (DVIT0358) | **315-315** | **290-290** | **243-257** | **271-271** | **179-181** | **241-241** | **243-249** | **183-187** | **79-81** | **159-167** | **145-151** | **182-202** | **167-175** | **206-210** | **353-371** | **236-238** | **263-263** | **149-151** | **180-188** |
| Bargoon (DVIT2499) | **315-328** | **290-294** | **233-235** | **251-255** | **189-194** | **246-246** | **233-253** | **165-165** | **77-79** | **165-167** | **135-151** | **164-188** | **163-165** | **210-214** | **355-360** | **228-240** | **263-263** | **151-155** | **178-184** |
| Barmak Isium (DVIT0310) | **319-319** | **290-306** | **257-259** | **243-271** | **185-189** | **241-247** | **239-245** | **165-183** | **83-83** | **155-157** | **137-137** | **166-202** | **165-165** | **206-206** | **353-362** | **234-238** | **256-263** | **149-151** | **178-194** |
| Baxtiori (156Mtp1) | **319-319** | **290-294** | **233-245** | **249-271** | **179-185** | **241-241** | **247-253** | **165-165** | **77-83** | **149-177** | **137-141** | **174-184** | **165-165** | **204-214** | **353-360** | **228-240** | **263-263** | **149-151** | **188-194** |
| Bayad (1749Mtp1) | **315-323** | **290-290** | **243-259** | **247-255** | **179-185** | **254-264** | **249-251** | **165-183** | **81-83** | **147-149** | **135-151** | **182-202** | **159-165** | **210-215** | **353-371** | **236-238** | **263-263** | **149-149** | **178-178** |
| Beli Potok (DVIT1841) | **-:-** | **-:-** | **235-253** | **239-255** | **189-189** | **-:-** | **263-263** | **165-167** | **77-77** | **149-149** | **151-151** | **174-178** | **149-163** | **204-212** | **355-363** | **228-234** | **263-263** | **149-151** | **184-192** |
| Berurargoon (DVIT2501) | **319-328** | **290-290** | **235-277** | **271-271** | **187-189** | **254-254** | **247-249** | **181-193** | **77-77** | **149-176** | **125-147** | **164-182** | **151-165** | **206-210** | **355-355** | **228-238** | **263-263** | **149-151** | **166-190** |
| Bez el Anza (S11981-0-2233-S1) | **315-328** | **290-306** | **233-253** | **257-271** | **185-194** | **254-264** | **239-253** | **181-183** | **79-83** | **151-159** | **133-141** | **174-188** | **167-177** | **206-206** | **353-353** | **232-232** | **263-263** | **149-151** | **172-184** |
| Bias Kukuzeti (DVIT0311) | **315-315** | **294-306** | **257-257** | **249-255** | **185-187** | **241-254** | **243-249** | **171-183** | **79-83** | **155-157** | **137-145** | **182-188** | **165-165** | **206-215** | **353-353** | **236-238** | **263-263** | **151-151** | **176-178** |

| | | | | | | | | | | | | | | | | | | | |
|---|---|---|---|---|---|---|---|---|---|---|---|---|---|---|---|---|---|---|---|
| Black Kishmish (DVIT2055) | **315-319** | **290-298** | **217-245** | **249-249** | **181-194** | **246-254** | **249-253** | **169-183** | **79-83** | **155-167** | **151-155** | **190-202** | **163-165** | **206-217** | **355-397** | **234-234** | **256-261** | **147-155** | **178-180** |
| Blanc d'Iran (Charif) (2086Mtp1) | **315-319** | **290-298** | **235-257** | **271-271** | **179-181** | **247-247** | **239-247** | **165-173** | **77-79** | **159-167** | **141-151** | **182-182** | **165-177** | **210-214** | **343-353** | **238-246** | **263-263** | **147-151** | **182-184** |
| Blanc De Crimei (DVIT0312) | **319-325** | **294-294** | **227-235** | **263-271** | **179-181** | **254-256** | **239-239** | **173-183** | **79-83** | **165-167** | **133-135** | **166-206** | **165-165** | **206-206** | **353-360** | **226-246** | **263-263** | **149-149** | **176-194** |
| Bouaki nor (2507Mtp1) | **315-319** | **290-294** | **234-257** | **249-259** | **185-194** | **241-247** | **243-243** | **165-183** | **79-83** | **155-155** | **141-155** | **172-202** | **177-177** | **206-210** | **353-360** | **234-236** | **256-267** | **151-155** | **184-188** |
| Boulany (2509Mtp1) | **319-326** | **294-294** | **235-257** | **261-271** | **179-183** | **246-255** | **239-239** | **-:-** | **77-83** | **159-167** | **143-145** | **166-166** | **151-165** | **206-206** | **-:-** | **228-228** | **263-267** | **149-149** | **158-194** |
| Boyalsing I (DVIT2496) | **289-319** | **290-306** | **235-245** | **251-261** | **185-189** | **247-254** | **243-251** | **165-165** | **83-83** | **159-159** | **133-135** | **182-190** | **141-163** | **204-206** | **347-353** | **228-228** | **263-265** | **151-151** | **166-190** |
| Boyalsing II (DVIT2505) | **315-328** | **290-294** | **245-257** | **251-255** | **181-185** | **241-247** | **243-251** | **165-173** | **77-79** | **167-173** | **143-155** | **172-182** | **151-165** | **210-214** | **355-355** | **240-240** | **263-263** | **149-151** | **172-178** |
| Buaki (DVIT0673) | **315-319** | **290-294** | **217-267** | **239-239** | **185-189** | **247-264** | **239-247** | **171-173** | **79-83** | **149-159** | **133-143** | **172-178** | **163-167** | **206-210** | **358-358** | **228-236** | **263-263** | **157-157** | **180-192** |
| Budrilay (DVIT2506) | **315-319** | **290-294** | **247-257** | **251-257** | **179-179** | **241-254** | **243-249** | **173-173** | **77-83** | **149-153** | **143-143** | **172-188** | **151-165** | **210-215** | **357-360** | **234-240** | **263-263** | **149-151** | **172-172** |
| Buraburi (DVIT2512) | **289-328** | **290-294** | **243-247** | **257-273** | **179-179** | **247-247** | **243-251** | **165-183** | **83-83** | **153-167** | **143-147** | **164-190** | **139-139** | **206-206** | **353-353** | **228-234** | **256-263** | **149-151** | **172-172** |
| Caus X Pearl Csaba (TYR VI 11-13 | **315-319** | **290-290** | **217-257** | **239-251** | **179-185** | **247-254** | **247-247** | **173-181** | **79-83** | **149-149** | **135-143** | **172-182** | **163-165** | **204-206** | **353-358** | **228-238** | **256-263** | **151-157** | **164-182** |
| Chaani biely (2675Mtp1) | **315-315** | **290-306** | **243-276** | **249-271** | **194-194** | **247-247** | **239-249** | **183-193** | **77-83** | **-:-** | **143-145** | **178-188** | **151-165** | **206-214** | **353-371** | **234-236** | **261-263** | **149-151** | **178-194** |
| Chaani noir (2676Mtp1) | **315-315** | **290-294** | **233-257** | **249-271** | **194-194** | **241-253** | **249-249** | **165-183** | **77-79** | **149-159** | **145-155** | **166-172** | **165-175** | **206-210** | **353-357** | **234-234** | **263-263** | **149-151** | **172-186** |
| Chakar angour de Tachkent (2677Mtp2) | **315-317** | **290-298** | **234-257** | **249-259** | **179-194** | **241-247** | **233-243** | **165-173** | **79-83** | **149-149** | **125-155** | **172-202** | **177-177** | **206-210** | **360-360** | **234-240** | **267-267** | **149-155** | **182-188** |
| Chamo (DVIT2730) | **289-289** | **290-290** | **243-247** | **243-273** | **179-187** | **-:-** | **249-251** | **165-181** | **77-83** | **167-167** | **143-153** | **186-190** | **165-165** | **206-210** | **355-360** | **234-236** | **263-263** | **149-151** | **172-172** |
| Chan Isium (DVIT0613) | **319-319** | **290-306** | **235-243** | **249-255** | **179-185** | **247-247** | **249-253** | **171-185** | **79-83** | **159-167** | **135-141** | **178-188** | **139-147** | **206-210** | **347-353** | **234-236** | **256-256** | **149-151** | **182-186** |

| | | | | | | | | | | | | | | | | | | | |
|---|---|---|---|---|---|---|---|---|---|---|---|---|---|---|---|---|---|---|---|
| Chaouch (DVIT0371) | 319-319 | 290-294 | 257-257 | 251-271 | 179-183 | 254-254 | 247-249 | 165-181 | 79-83 | 149-171 | 135-151 | 182-188 | 159-165 | 204-215 | 353-353 | 228-238 | 256-263 | 149-151 | 182-186 |
| Charas (DVIT0685) | 315-319 | 290-290 | 243-243 | 257-257 | 179-185 | 246-254 | 245-249 | 165-183 | 77-83 | 173-177 | 125-135 | 172-182 | 165-165 | 206-206 | 353-360 | 226-228 | 256-256 | 149-151 | 184-190 |
| Charli Sar (TYR VI 12-05) | 315-327 | -:- | 243-243 | -:- | 183-194 | -:- | 249-253 | 165-183 | 79-83 | 173-177 | 137-151 | 182-188 | -:- | -:- | -:- | 240-240 | 256-263 | 149-155 | 182-182 |
| Chassany (0Mtp225) | 319-330 | 294-298 | 239-257 | 271-271 | 183-194 | 241-247 | 239-247 | 171-173 | 75-83 | 155-163 | 137-141 | 174-174 | 151-163 | 206-215 | 353-353 | 226-236 | 256-263 | 149-151 | 178-190 |
| Chirai obak (1186Mtp1) | 315-317 | 294-294 | 235-243 | 249-255 | 181-194 | 241-246 | 243-243 | 165-169 | 79-83 | 145-177 | 137-143 | 172-182 | 151-165 | 206-206 | 353-366 | 236-240 | 256-263 | 151-151 | 158-188 |
| Chirvan chakki (2678Mtp2) | 315-315 | 294-294 | 243-247 | 249-271 | 192-194 | 241-253 | 245-249 | 165-165 | 77-79 | 149-159 | 143-145 | 166-172 | 163-165 | 204-208 | 353-353 | 232-234 | 263-263 | 149-151 | 178-194 |
| Chol Uzyum (Turkmn 16392) | 315-319 | 290-294 | 233-243 | 257-271 | 194-194 | 241-256 | 249-263 | 165-183 | 79-83 | 149-149 | 143-143 | 186-192 | 167-175 | 206-210 | 353-357 | 228-236 | 263-263 | 151-151 | 186-194 |
| Chtour angour (2857Mtp1) | 319-327 | 290-290 | 243-247 | 255-257 | 185-194 | 241-247 | 243-253 | 165-165 | 79-83 | 149-149 | 135-151 | 182-190 | 139-165 | 206-215 | 357-375 | 234-236 | 256-263 | 151-151 | 174-186 |
| Churgoon (DVIT2532) | 289-315 | 294-306 | 235-235 | 243-251 | 185-185 | 246-246 | 243-251 | 165-165 | 77-79 | 149-149 | 125-151 | 188-202 | 139-139 | 206-206 | 360-360 | 238-240 | 263-265 | 149-149 | 188-194 |
| Cirmisi Sap De Sudak (DVIT0313) | 315-319 | 290-294 | 243-257 | 255-271 | 185-185 | 241-254 | 249-249 | 171-173 | 83-83 | 155-177 | 137-145 | 174-188 | 165-175 | 206-215 | 353-353 | 236-236 | 263-263 | 146-151 | 178-178 |
| Coudsi (DVIT0384) | 315-315 | 290-294 | 247-257 | 261-271 | 179-185 | 241-254 | 247-247 | 169-187 | 79-81 | 149-157 | 143-151 | 186-202 | 165-175 | 204-206 | 347-353 | 236-236 | 263-263 | 151-151 | 178-182 |
| Dabouki (DVIT0388) | 323-323 | 290-290 | 257-257 | 249-271 | 179-183 | 241-254 | 247-249 | 165-187 | 79-81 | 157-157 | 135-151 | 202-202 | 165-175 | 206-220 | 353-353 | 234-236 | 263-263 | 149-151 | 178-182 |
| Dabouki (DVIT1982) | 315-323 | 290-290 | 257-257 | 249-271 | 179-185 | 247-264 | 247-249 | 165-187 | 79-81 | 149-157 | 143-151 | 202-202 | 165-175 | 206-220 | 353-353 | 228-236 | 263-263 | 149-151 | 182-190 |
| Dais-el-anz (DVIT0569) | 315-319 | 290-298 | 237-257 | 249-271 | 179-194 | 247-254 | 249-249 | 165-169 | 79-79 | 149-167 | 151-155 | 182-182 | 167-177 | 206-206 | 353-357 | 234-236 | 263-263 | 151-151 | 178-178 |
| Dalnato I (DVIT2509) | 328-328 | 290-290 | 245-257 | 251-271 | 179-185 | 241-247 | 247-255 | 165-165 | 75-77 | 149-167 | 135-155 | 182-190 | 163-165 | 214-214 | 353-353 | 236-240 | 263-263 | 149-151 | 172-190 |
| Dardari (DVIT2536) | 289-315 | 290-294 | 245-247 | 251-261 | 179-189 | 246-254 | 233-243 | 165-165 | 77-77 | 159-159 | 133-135 | 182-188 | 139-139 | 204-206 | 353-360 | 228-240 | 256-263 | 149-151 | 190-190 |
| De Hengril (857Mtp1) | 315-328 | 294-294 | 233-235 | 249-271 | 181-189 | 241-247 | 243-249 | 165-183 | 79-83 | 149-163 | 135-145 | 186-186 | 163-165 | 206-215 | 353-357 | 234-240 | 263-265 | 151-151 | 178-188 |

| | | | | | | | | | | | | | | | | | | | |
|---|---|---|---|---|---|---|---|---|---|---|---|---|---|---|---|---|---|---|---|
| Demir Kara (DVIT0314) | **315-323** | **290-294** | **257-257** | **249-271** | **179-187** | **246-254** | **239-253** | **181-183** | **77-77** | **157-167** | **135-145** | **166-166** | **165-165** | **206-206** | **353-371** | **232-240** | **256-267** | **149-149** | **176-184** |
| DK #01 (Turkmn 13358) | **315-328** | **290-298** | **217-277** | **239-251** | **181-187** | **246-246** | **249-249** | **173-183** | **77-79** | **155-155** | **125-139** | **182-188** | **151-167** | **204-214** | **353-357** | **232-248** | **256-256** | **151-151** | **188-190** |
| DK #02 (Turkmn 13359) | **315-328** | **288-306** | **233-247** | **251-251** | **179-179** | **247-247** | **235-243** | **173-183** | **79-83** | **155-155** | **133-139** | **164-182** | **139-165** | **210-215** | **353-360** | **226-228** | **256-263** | **151-151** | **192-194** |
| DK #03 (Turkmn 13360) | **315-328** | **290-298** | **257-257** | **247-249** | **179-179** | **241-254** | **235-251** | **165-169** | **79-79** | **153-177** | **139-153** | **182-188** | **139-147** | **206-210** | **353-353** | **234-240** | **256-263** | **149-149** | **188-190** |
| DK #04 (Turkmn 13361) | **319-319** | **294-298** | **277-277** | **255-257** | **187-187** | **247-247** | **235-249** | **165-169** | **79-83** | **167-167** | **137-139** | **186-188** | **139-165** | **204-210** | **353-353** | **234-236** | **256-263** | **151-151** | **174-194** |
| DK #05 (Turkmn 13362) | **319-319** | **298-298** | **247-277** | **257-271** | **187-187** | **-:-** | **235-249** | **169-183** | **77-79** | **167-173** | **137-143** | **186-188** | **139-151** | **210-215** | **353-360** | **234-240** | **256-263** | **151-151** | **174-190** |
| DK #07 (Turkmn 13364) | **315-328** | **294-294** | **243-257** | **251-261** | **179-194** | **254-254** | **235-253** | **165-193** | **83-83** | **149-177** | **135-151** | **182-182** | **147-159** | **206-214** | **353-353** | **234-240** | **256-256** | **151-151** | **186-194** |
| DK #08 (Turkmn 13365) | **319-319** | **290-294** | **233-257** | **251-271** | **185-194** | **241-247** | **251-263** | **165-173** | **77-79** | **145-145** | **135-135** | **178-188** | **139-151** | **204-204** | **358-358** | **234-238** | **256-267** | **151-151** | **174-190** |
| DK #10 (Turkmn 13388) | **315-315** | **-:-** | **233-247** | **249-271** | **179-187** | **-:-** | **235-253** | **165-173** | **79-83** | **149-177** | **125-153** | **164-188** | **147-147** | **-:-** | **353-353** | **234-234** | **256-263** | **149-151** | **184-190** |
| DK #11 (Turkmn 13387) | **299-301** | **290-294** | **235-243** | **249-255** | **179-187** | **241-247** | **235-249** | **173-173** | **83-83** | **149-155** | **-:-** | **182-188** | **147-165** | **214-214** | **353-353** | **226-234** | **256-263** | **151-151** | **184-188** |
| DK #12 (Turkmn 13386) | **319-319** | **272-290** | **233-233** | **249-257** | **179-194** | **241-247** | **235-253** | **165-165** | **77-79** | **145-167** | **125-137** | **172-190** | **139-165** | **206-215** | **357-360** | **228-236** | **256-263** | **149-151** | **174-188** |
| DK #17 (Turkmn 13381) | **328-328** | **290-312** | **271-277** | **239-251** | **185-187** | **247-247** | **247-249** | **165-183** | **77-79** | **155-173** | **133-139** | **178-188** | **139-167** | **206-214** | **336-357** | **232-234** | **256-263** | **151-151** | **184-190** |
| DK #2 (Turkmn 13389) | **317-317** | **290-294** | **243-247** | **249-271** | **179-187** | **247-247** | **235-237** | **173-193** | **79-83** | **149-177** | **133-133** | **188-188** | **147-147** | **210-214** | **353-353** | **232-234** | **263-267** | **151-151** | **182-188** |
| DK #21 Chernyi (Turkmn 13379) | **328-328** | **290-312** | **271-277** | **239-251** | **179-194** | **247-247** | **247-249** | **165-183** | **77-79** | **155-173** | **133-133** | **178-188** | **139-167** | **206-214** | **336-357** | **232-234** | **256-263** | **151-151** | **184-190** |
| DK #39 (Turkmn 13377) | **319-319** | **294-294** | **233-243** | **247-257** | **179-189** | **247-247** | **235-251** | **165-183** | **79-79** | **155-173** | **133-135** | **164-164** | **147-151** | **206-214** | **353-360** | **226-226** | **256-256** | **151-151** | **178-182** |
| DK #9 (Turkmn 6272) | **319-319** | **290-294** | **235-235** | **247-251** | **185-187** | **241-247** | **235-249** | **-:-** | **83-83** | **-:-** | **133-135** | **178-188** | **147-165** | **206-215** | **353-360** | **226-234** | **-:-** | **151-151** | **178-186** |
| DK Belyi (Turkmn 6977) | **299-301** | **290-294** | **235-243** | **249-255** | **179-187** | **241-247** | **235-253** | **173-173** | **83-83** | **149-155** | **133-133** | **164-188** | **147-165** | **215-215** | **353-353** | **234-234** | **256-263** | **151-151** | **184-188** |

| | | | | | | | | | | | | | | | | | | | |
|---|---|---|---|---|---|---|---|---|---|---|---|---|---|---|---|---|---|---|---|
| DK Melkii Chernyi (Turkmn 13375) | 303-311 | 290-290 | 233-235 | 239-239 | 175-189 | 247-256 | 239-239 | 183-183 | 77-83 | 159-159 | 133-151 | 172-178 | 165-179 | 206-215 | 360-369 | 232-240 | 263-267 | 151-151 | 186-188 |
| DK Melkii Krasnyi (Turkmn 13374) | 315-315 | 288-294 | 243-245 | 247-249 | 179-181 | 241-247 | 247-247 | 183-183 | 77-77 | 153-159 | 133-133 | 182-188 | 139-165 | 206-214 | -:- | 236-246 | -:- | 149-151 | 182-190 |
| DK N15 (#15) (Turkmn 30743) | 315-328 | 288-294 | 243-245 | 243-257 | 179-185 | 241-247 | 235-243 | 169-169 | 77-79 | 153-155 | -:- | 182-202 | 159-159 | 206-206 | 353-360 | 234-240 | 256-256 | 151-151 | 170-186 |
| Doppelaugen (0Mtp318) | 315-319 | 290-294 | 234-257 | 261-271 | 181-185 | 241-246 | 245-249 | 171-183 | 79-83 | 155-159 | 135-143 | 166-174 | 151-169 | 206-215 | 353-353 | 236-236 | 256-265 | 149-151 | 178-190 |
| Dschan Im Isium (DVIT0315) | 315-319 | 290-294 | 243-257 | 249-271 | 179-185 | 246-254 | 239-249 | 171-173 | 79-83 | 159-177 | 145-145 | 168-188 | 175-177 | 206-215 | 353-353 | 228-236 | 263-267 | 149-151 | 178-188 |
| Ekdona Turkmenskaya (Turkmn 6218) | 315-317 | 290-298 | 234-257 | 249-259 | 179-194 | 241-247 | 233-243 | 165-173 | 79-83 | 149-149 | 125-133 | 172-202 | 175-177 | 206-210 | 360-360 | 234-240 | 267-267 | 149-155 | 180-188 |
| Fachren Weis (DVIT0316) | 317-317 | 294-298 | 235-243 | 249-271 | 189-194 | 241-254 | 239-239 | 165-171 | 83-83 | 149-177 | 133-145 | 174-188 | 155-165 | 206-215 | 353-353 | 234-240 | 263-263 | 149-151 | 178-182 |
| Fahri (DVIT0402) | 315-319 | 290-290 | 234-259 | 255-271 | 185-194 | 241-247 | 253-253 | 165-165 | 79-79 | 155-177 | 141-151 | 182-182 | 165-175 | 210-215 | 353-375 | 234-240 | 263-263 | 147-155 | 184-190 |
| Fatai (DVIT2510) | 289-289 | 290-306 | 235-235 | 261-271 | 179-189 | 254-254 | 239-249 | 165-181 | 83-83 | 153-173 | 141-145 | 182-188 | 139-165 | 204-206 | 347-358 | 228-234 | 256-263 | -:- | 172-194 |
| Fayoumi (DVIT2636) | 328-328 | 290-306 | 251-255 | -:- | 185-194 | 247-264 | 249-253 | 181-181 | 81-83 | 151-155 | 141-143 | 174-186 | -:- | 206-215 | -:- | 232-232 | 263-263 | 151-151 | 172-184 |
| Fetyaska (S10000-0-4432-S1) | 327-327 | 290-294 | 247-259 | 247-251 | 185-194 | 247-247 | 247-253 | 171-183 | 77-83 | 155-159 | 133-133 | 172-202 | 139-167 | 206-206 | 353-357 | 226-236 | 256-265 | 149-149 | 174-194 |
| Gaschochi (DVIT2537) | 289-317 | 290-306 | 235-245 | 243-261 | 181-189 | 247-254 | 239-243 | 165-165 | 83-83 | 159-173 | 135-145 | 182-190 | 139-141 | 204-214 | 347-361 | 228-246 | 256-265 | 151-151 | 188-192 |
| Gechi Kyrlen (Turkmn 3028) | 315-319 | 290-294 | 235-247 | 271-271 | 179-179 | 247-247 | 253-253 | 187-193 | 79-83 | 159-177 | 135-153 | 186-188 | 165-165 | 210-210 | 353-353 | 234-234 | 256-256 | 149-151 | 186-186 |
| Golodan (0Mtp428) | 319-319 | 290-294 | 217-243 | 249-249 | 179-194 | 247-254 | 239-253 | 165-173 | 79-79 | 149-159 | 137-145 | 172-188 | 139-165 | 206-215 | 353-360 | 234-240 | 261-263 | 151-151 | 178-184 |
| Gora Chirine faux (0Mtp429) | 315-317 | 298-298 | 217-243 | 249-249 | 181-194 | 247-247 | 239-239 | 183-183 | 79-83 | 155-177 | 145-151 | 188-188 | 163-165 | 206-215 | 353-353 | 234-234 | 263-263 | 151-155 | 178-182 |
| Gros Colman (DVIT0413) | 315-323 | 294-294 | 227-243 | 251-271 | 179-185 | 247-247 | 247-255 | 173-183 | 77-79 | 155-159 | 133-135 | 166-166 | 163-165 | 206-206 | 353-353 | 226-236 | 256-256 | 149-149 | 186-188 |

| | | | | | | | | | | | | | | | | | | | |
|---|---|---|---|---|---|---|---|---|---|---|---|---|---|---|---|---|---|---|---|
| Gungargoon (DVIT2500) | **319-328** | **290-294** | **243-259** | **257-271** | **194-194** | **247-247** | **247-253** | **165-173** | **77-79** | **155-155** | **135-141** | **182-182** | **139-165** | **-:-** | **353-357** | **236-240** | **263-263** | **149-155** | **178-186** |
| Gurgon (Turkmn 3033) | **315-328** | **290-294** | **217-257** | **249-249** | **179-187** | **246-254** | **235-253** | **165-165** | **77-83** | **173-177** | **133-135** | **164-188** | **147-175** | **210-215** | **347-353** | **234-240** | **256-263** | **151-151** | **186-190** |
| Guzal Kara (TYR VI 13-09) | **319-323** | **294-298** | **227-233** | **251-271** | **185-194** | **249-249** | **243-247** | **183-193** | **79-79** | **153-155** | **135-151** | **166-172** | **163-177** | **206-210** | **353-375** | **226-240** | **256-263** | **149-155** | **178-186** |
| Haita safid (0Mtp471) | **315-315** | **290-294** | **243-243** | **249-251** | **179-185** | **247-254** | **249-249** | **165-173** | **77-83** | **155-177** | **133-135** | **180-188** | **165-165** | **210-215** | **368-368** | **234-234** | **256-263** | **155-155** | **182-186** |
| Halili beliij (DVIT0430) | **315-317** | **290-294** | **243-257** | **249-261** | **179-185** | **247-254** | **235-249** | **165-193** | **79-83** | **159-159** | **125-135** | **172-182** | **147-165** | **206-210** | **353-361** | **234-240** | **256-267** | **149-151** | **186-186** |
| Halili Nobat Niyaz (Turkmn 6971) | **299-315** | **294-294** | **233-257** | **257-261** | **179-185** | **247-247** | **235-249** | **193-193** | **83-83** | **145-145** | **133-141** | **182-182** | **165-165** | **206-218** | **353-353** | **236-240** | **256-263** | **149-151** | **182-186** |
| Hassaine (DVIT0416) | **319-319** | **290-290** | **234-259** | **249-255** | **194-194** | **241-247** | **243-253** | **173-183** | **79-79** | **-:-** | **147-151** | **182-188** | **165-177** | **210-215** | **353-353** | **224-228** | **256-263** | **151-155** | **184-184** |
| Henab (DVIT0417) | **315-323** | **290-306** | **243-257** | **255-273** | **181-194** | **241-247** | **243-253** | **183-183** | **77-83** | **157-167** | **143-149** | **164-202** | **139-177** | **215-215** | **353-353** | **226-232** | **263-263** | **151-151** | **188-188** |
| Himrisnky (DVIT0328) | **307-315** | **290-290** | **243-257** | **239-255** | **179-183** | **247-252** | **239-239** | **171-183** | **83-83** | **161-173** | **139-151** | **178-206** | **165-166** | **206-206** | **353-360** | **226-238** | **263-263** | **149-157** | **178-190** |
| Hisakasy (DVIT0759) | **315-319** | **294-294** | **233-257** | **251-271** | **194-194** | **247-247** | **245-249** | **171-183** | **83-83** | **167-177** | **135-143** | **178-202** | **151-165** | **206-210** | **353-353** | **234-236** | **263-263** | **149-151** | **176-178** |
| Hiv Uzyum (Turkmn 19806) | **319-321** | **290-298** | **233-243** | **249-257** | **194-194** | **241-254** | **243-247** | **165-183** | **79-79** | **167-167** | **133-135** | **182-182** | **153-165** | **206-214** | **-:-** | **226-234** | **263-263** | **151-155** | **186-194** |
| Hosargoon (DVIT2503) | **317-317** | **290-306** | **243-247** | **255-255** | **185-185** | **246-246** | **249-251** | **165-169** | **77-79** | **149-153** | **133-135** | **164-202** | **165-165** | **206-210** | **355-355** | **226-232** | **263-263** | **149-151** | **172-178** |
| Hunisa (635Mtp1) | **315-315** | **290-290** | **243-243** | **249-273** | **181-194** | **247-264** | **239-253** | **183-187** | **77-83** | **151-157** | **143-149** | **164-202** | **139-159** | **206-215** | **353-355** | **228-232** | **263-263** | **149-151** | **172-188** |
| Huseine Rozvoj (TYR VI 13-15) | **315-319** | **290-290** | **243-243** | **249-257** | **185-194** | **246-246** | **243-249** | **165-173** | **77-79** | **149-173** | **135-155** | **182-202** | **165-177** | **206-210** | **353-353** | **228-234** | **256-263** | **149-155** | **182-184** |
| Husseine (DVIT0576) | **313-315** | **290-298** | **243-245** | **257-271** | **179-194** | **241-249** | **233-243** | **165-193** | **77-79** | **173-177** | **141-155** | **172-172** | **139-139** | **215-215** | **353-353** | **234-240** | **263-263** | **149-149** | **178-186** |
| Irki (1752Mtp1) | **315-323** | **290-306** | **243-243** | **255-271** | **179-185** | **254-264** | **239-249** | **183-187** | **77-83** | **147-147** | **143-151** | **182-182** | **165-165** | **206-210** | **353-371** | **238-246** | **261-263** | **149-151** | **176-178** |
| Irtyk Yaprak (Turkmn 3026) | **315-319** | **290-294** | **247-257** | **-:-** | **179-185** | **241-247** | **235-251** | **183-193** | **79-83** | **159-159** | **135-135** | **188-188** | **129-139** | **204-210** | **-:-** | **234-240** | **256-263** | **151-151** | **182-184** |

| | | | | | | | | | | | | | | | | | | | |
|---|---|---|---|---|---|---|---|---|---|---|---|---|---|---|---|---|---|---|---|
| Itchkimar biely faux (2845Mtp1) | 319-327 | 290-290 | 233-243 | 255-271 | 179-185 | 241-248 | 249-253 | 165-183 | 77-83 | 155-155 | 143-151 | 188-200 | 165-165 | 206-206 | 347-371 | 234-240 | 256-256 | 151-151 | 176-186 |
| Jane De Smirna (TYR VI 13-17) | 315-319 | 288-290 | 235-276 | 261-263 | 183-194 | 247-247 | 239-247 | 171-183 | 77-83 | 149-167 | 139-143 | 172-188 | 165-177 | 206-206 | 361-371 | 228-228 | 263-263 | 149-149 | 176-194 |
| Kabuli (DVIT2497) | 315-317 | 290-294 | 235-245 | 257-271 | 185-187 | 246-254 | 239-249 | 165-169 | 83-83 | 149-159 | 135-145 | 172-182 | 139-141 | 210-214 | 353-361 | 228-234 | 263-265 | 151-151 | 170-190 |
| Kala Kostan (DVIT2534) | 315-319 | 290-294 | 243-247 | -:- | 177-194 | 247-254 | 253-253 | 169-183 | 83-83 | 147-155 | 129-145 | 190-206 | 151-163 | 207-215 | -:- | 234-238 | 254-263 | 155-155 | 180-180 |
| Kalamak (DVIT0335) | 319-328 | 290-304 | 243-280 | 249-271 | 179-194 | 247-247 | 247-253 | 165-183 | 79-83 | 149-167 | 137-141 | 182-182 | 165-165 | 206-214 | 360-375 | 232-234 | 256-263 | 151-155 | 184-186 |
| Kali Sag (DVIT2917) | 315-319 | 290-290 | 217-234 | 237-253 | 181-194 | 247-254 | 247-253 | 165-173 | 77-79 | 149-177 | 141-153 | 182-188 | 165-177 | 210-215 | -:- | 234-240 | 263-263 | 147-155 | 180-186 |
| Kandahar (DVIT0428) | 317-317 | 290-290 | 217-233 | 249-255 | 194-194 | 249-254 | 239-253 | 165-165 | 79-83 | 153-177 | 151-155 | 172-188 | 165-175 | 215-218 | 353-353 | 234-240 | 263-263 | 149-151 | 182-190 |
| Kandhari (DVIT2081) | 319-319 | 290-298 | 243-257 | 249-255 | 189-194 | 254-254 | 243-247 | 165-165 | 77-77 | 177-177 | 143-151 | 188-190 | 163-165 | 210-218 | 360-360 | 234-236 | 263-263 | 151-155 | 162-190 |
| Kanfet isium (0Mtp565) | 319-319 | 294-294 | 239-257 | 249-271 | 193-194 | 241-249 | 245-259 | 173-183 | 83-83 | 165-167 | 143-143 | 178-202 | 151-165 | 206-210 | 368-368 | 234-234 | 256-263 | 149-151 | 178-190 |
| Kara Dzhidzhigi (DVIT2322) | 319-319 | 290-298 | 234-245 | 249-255 | 181-185 | 241-254 | 249-253 | 165-169 | 83-83 | 155-155 | 141-155 | 182-202 | 165-165 | 206-214 | 360-360 | 234-236 | 256-256 | 149-155 | 178-186 |
| Kara Lakana (DVIT0773) | 315-319 | 294-294 | 247-257 | 243-261 | -:- | 247-256 | 239-249 | 165-183 | 79-83 | 159-167 | 145-145 | 166-178 | 163-177 | 206-206 | 353-357 | 226-240 | 263-263 | 149-151 | 180-204 |
| Kara Palvan (2780Mtp1) | 319-327 | 290-290 | 243-243 | 257-257 | 179-194 | 241-254 | 249-253 | 165-183 | 77-79 | 155-177 | 125-151 | 172-182 | 139-165 | 206-206 | 353-357 | 234-240 | 263-263 | 151-155 | 172-172 |
| Kara Terbash (Turkmn 6982) | 315-319 | 290-290 | 243-257 | 261-271 | 179-185 | 241-254 | 235-253 | 165-173 | 77-79 | 159-159 | 125-151 | 182-182 | 147-165 | 206-214 | 353-361 | 234-236 | 256-256 | 149-151 | 186-186 |
| Kara Uzyum Ashhabadskii (Turkmn 551) | 319-319 | 290-306 | 247-247 | 271-271 | 179-185 | 241-247 | 247-253 | 165-193 | 77-83 | 155-159 | 135-141 | 174-188 | 165-165 | 206-206 | 353-358 | 236-240 | 256-263 | 151-151 | 186-190 |
| Kara Uzyum Nuhurskii (Turkmn 3036) | 319-328 | 290-294 | 257-271 | 251-271 | 179-187 | 247-254 | 235-253 | 173-193 | 79-83 | 149-159 | 135-151 | 182-188 | 139-147 | 210-214 | 336-353 | 234-236 | 256-256 | 149-151 | 186-188 |
| Karadzhandal (DVIT2323) | 315-319 | 290-294 | 233-257 | 249-271 | 179-194 | 247-247 | 243-249 | 165-169 | 77-79 | 177-177 | 125-155 | 182-202 | 139-139 | 210-214 | 353-360 | 234-240 | 263-267 | 155-155 | 182-186 |

| | | | | | | | | | | | | | | | | | | | |
|---|---|---|---|---|---|---|---|---|---|---|---|---|---|---|---|---|---|---|---|
| Karga Dili (Turkmn 21551) | **315-319** | **294-306** | **243-247** | **255-271** | **185-185** | **246-246** | **239-247** | **165-171** | **77-83** | **155-159** | **139-141** | **172-178** | **165-165** | **206-206** | **353-358** | **226-236** | **256-263** | **151-151** | **178-190** |
| Kash Uzyum (Turkmn 6981) | **315-319** | **290-306** | **235-235** | **249-251** | **185-194** | **241-241** | **251-253** | **165-183** | **77-83** | **155-155** | **141-143** | **178-202** | **165-175** | **204-206** | **353-360** | **236-240** | **263-263** | **147-151** | **186-190** |
| Kashiri (DVIT2451) | **289-328** | **290-306** | **235-235** | **243-271** | **181-185** | **247-247** | **239-249** | **165-165** | **83-83** | **159-159** | **135-145** | **164-182** | **139-141** | **204-206** | **355-361** | **228-228** | **263-265** | **151-151** | **172-194** |
| Katta Kurgan (DVIT0774) | **315-319** | **290-298** | **234-234** | **255-271** | **179-194** | **247-249** | **243-253** | **165-193** | **77-79** | **153-177** | **151-155** | **172-182** | **175-177** | **210-218** | **353-353** | **240-240** | **263-263** | **149-155** | **178-190** |
| Kaytagi (2951Mtp1) | **315-319** | **290-294** | **257-259** | **247-271** | **181-194** | **247-249** | **245-259** | **165-171** | **83-83** | **167-177** | **135-135** | **178-186** | **165-165** | **206-210** | **353-371** | **234-234** | **263-263** | **151-151** | **176-188** |
| Kechmisch aly violet (1679Mtp2) | **315-319** | **290-294** | **243-247** | **251-271** | **185-194** | **247-264** | **243-249** | **167-187** | **77-83** | **157-163** | **135-149** | **168-202** | **165-165** | **206-208** | **357-360** | **226-246** | **263-263** | **147-149** | **182-184** |
| Keshmesh Heshrau (Turkmn 29892) | **315-319** | **290-298** | **217-243** | **-:-** | **181-194** | **247-254** | **243-249** | **169-183** | **79-83** | **155-155** | **151-151** | **188-202** | **-:-** | **206-206** | **-:-** | **228-234** | **256-256** | **147-151** | **182-184** |
| Khalchili (DVIT0431) | **315-319** | **298-298** | **243-245** | **249-271** | **181-194** | **247-247** | **243-253** | **165-183** | **77-79** | **155-173** | **143-145** | **172-186** | **165-177** | **206-218** | **353-360** | **238-240** | **263-267** | **149-155** | **190-194** |
| Khaldar (DVIT0432) | **317-319** | **290-290** | **243-257** | **257-271** | **179-179** | **254-254** | **247-249** | **165-183** | **77-77** | **157-159** | **135-151** | **182-188** | **165-175** | **210-210** | **353-360** | **226-234** | **263-263** | **151-155** | **190-194** |
| Khalili (DVIT2084) | **317-319** | **290-290** | **234-243** | **249-249** | **185-194** | **241-247** | **239-253** | **165-183** | **79-83** | **155-177** | **145-151** | **182-188** | **163-165** | **215-215** | **353-375** | **234-234** | **263-263** | **151-155** | **182-186** |
| Khalili tcherni (2663Mtp2) | **317-319** | **290-294** | **234-243** | **249-257** | **185-194** | **241-254** | **247-249** | **165-165** | **77-83** | **159-159** | **125-151** | **172-182** | **139-165** | **206-214** | **353-361** | **228-240** | **256-263** | **151-151** | **172-186** |
| Khatmi (2190Mtp1) | **315-315** | **294-294** | **257-259** | **249-271** | **185-194** | **246-247** | **249-249** | **171-183** | **83-83** | **157-177** | **135-143** | **174-178** | **151-167** | **206-210** | **371-371** | **224-236** | **256-263** | **151-151** | **178-184** |
| Khawngi (DVIT2919) | **317-317** | **294-296** | **225-235** | **247-271** | **183-183** | **247-247** | **235-249** | **169-175** | **77-77** | **155-155** | **123-151** | **172-172** | **167-167** | **206-206** | **344-360** | **238-238** | **263-263** | **151-155** | **176-178** |
| Khindogny (2664Mtp1) | **319-319** | **290-298** | **257-270** | **257-271** | **181-194** | **241-247** | **239-249** | **165-183** | **79-79** | **149-159** | **133-155** | **178-178** | **151-165** | **210-215** | **357-363** | **226-234** | **263-269** | **149-151** | **182-194** |
| Khir Ghulaman (DVIT0434) | **319-319** | **290-298** | **241-251** | **249-255** | **181-194** | **247-247** | **243-261** | **171-187** | **79-83** | **159-167** | **125-133** | **188-204** | **147-161** | **204-214** | **329-353** | **234-244** | **254-254** | **-:-** | **176-184** |
| Khorestini (DVIT2605) | **315-319** | **290-290** | **235-243** | **255-257** | **194-194** | **-:-** | **247-251** | **165-165** | **79-81** | **149-177** | **153-155** | **188-202** | **165-177** | **206-210** | **353-360** | **234-240** | **263-263** | **-:-** | **184-188** |
| Kibraïski (2781Mtp1) | **315-319** | **294-298** | **245-257** | **249-271** | **181-183** | **247-254** | **249-253** | **165-169** | **83-83** | **155-171** | **135-155** | **182-190** | **159-165** | **215-218** | **353-353** | **234-238** | **256-263** | **147-151** | **182-182** |

| | | | | | | | | | | | | | | | | | | | |
|---|---|---|---|---|---|---|---|---|---|---|---|---|---|---|---|---|---|---|---|
| Kichmich rond (1678Mtp5) | 317-319 | 290-290 | 243-247 | 249-271 | 181-189 | 241-254 | 239-249 | 165-165 | 79-79 | 159-177 | 145-151 | 178-188 | 163-165 | 206-210 | 371-373 | 228-234 | 261-263 | 151-155 | 178-194 |
| Kini Yatch (DVIT2495) | 319-328 | 290-294 | 233-243 | 247-261 | 179-179 | 247-247 | 241-249 | 165-175 | 75-79 | 159-159 | 131-135 | 188-188 | 139-163 | 204-210 | 347-355 | 228-236 | 256-263 | 149-149 | 172-174 |
| Kishmish Of Vir (DVIT2071) | 315-319 | 290-298 | 233-245 | 249-249 | 185-194 | 241-247 | 247-253 | 165-169 | 79-83 | 155-177 | 141-155 | 186-204 | 139-163 | 204-218 | 360-360 | 224-234 | 261-263 | 151-155 | 182-188 |
| Kishmish Sorkh (DVIT0437) | 315-315 | 294-298 | 217-233 | 249-271 | 179-194 | 247-247 | 233-253 | 165-193 | 77-79 | 155-159 | 141-151 | 172-188 | 129-139 | 206-215 | -:- | 234-246 | 261-263 | -:- | 174-182 |
| Kishmishi (TYR VI 14-19) | 315-328 | 290-294 | 217-217 | 249-255 | 185-194 | 241-247 | 243-253 | 165-193 | 83-83 | 155-167 | 141-151 | 182-188 | 139-165 | 206-210 | -:- | 234-240 | 263-263 | 151-155 | -:- |
| Kishmishi (DVIT0435) | 319-319 | 290-290 | 234-243 | 249-255 | 179-181 | 241-247 | 249-249 | 165-165 | 79-79 | 177-177 | 137-141 | -:- | 139-165 | 214-215 | 360-375 | 234-246 | 263-263 | 151-155 | 184-186 |
| Kisil izium (0Mtp589) | 315-319 | 290-294 | 233-234 | 249-249 | 179-179 | 241-247 | 245-253 | 183-183 | 79-83 | 153-177 | 135-153 | 178-182 | 147-165 | 206-210 | 353-353 | 240-240 | 256-256 | 151-151 | 178-186 |
| Kisil sapak (746Mtp1) | 315-319 | 294-306 | 257-270 | 271-271 | 179-187 | 246-247 | 233-253 | 165-173 | 83-83 | 149-159 | 135-153 | 182-188 | 147-165 | 206-214 | 337-353 | 236-240 | 256-256 | 149-149 | 184-186 |
| Kismis Vatkana (20008-14 B) | 315-319 | 294-298 | 217-233 | 249-271 | 179-194 | 247-254 | 239-249 | 165-183 | 77-83 | 177-177 | 137-145 | 164-190 | 139-163 | 206-215 | 353-353 | 234-240 | 261-263 | -:- | 178-182 |
| Kismish Turkmenskii (Turkmn 19697) | 317-317 | 290-298 | 243-257 | 249-271 | 181-181 | -:- | 233-253 | 183-193 | 79-83 | 155-177 | 135-145 | 188-188 | 165-165 | 210-215 | 353-353 | -:- | 256-261 | 149-155 | 182-186 |
| Kizil Sapak (Turkmn 545) | 319-319 | 290-294 | 235-257 | 249-251 | 179-185 | 247-247 | 243-253 | 185-193 | 79-83 | 159-159 | 133-135 | 164-188 | 147-165 | 206-210 | 347-353 | 232-234 | 256-256 | 149-151 | 178-186 |
| Kizil sapak (0Mtp414) | 319-321 | 290-290 | 245-247 | 249-251 | 179-185 | 247-249 | 247-253 | 165-185 | 83-83 | 159-159 | 133-153 | 182-188 | 165-165 | 206-206 | 347-353 | 234-234 | 256-256 | 149-151 | 176-186 |
| Kondori 01 (2001-9-8100-01) | 315-319 | 290-294 | 234-257 | 249-249 | 185-193 | 246-246 | 243-247 | 165-171 | 75-79 | 155-155 | 137-151 | 174-182 | 165-167 | 206-215 | 353-360 | 236-240 | 256-263 | -:- | 188-192 |
| Koptcha (DVIT0783) | 315-327 | 290-294 | 227-247 | 251-271 | 181-181 | 247-255 | 239-249 | 169-183 | 77-79 | 155-159 | 133-143 | 168-204 | -:- | 206-206 | 353-360 | 238-240 | 263-263 | 149-149 | 174-182 |
| Korza erevani (0Mtp610) | 315-315 | 290-298 | 217-235 | 249-271 | 185-194 | 241-254 | 239-247 | 177-183 | 83-83 | 155-155 | 137-151 | 188-188 | 165-165 | 215-215 | 357-357 | 234-240 | 261-263 | 151-155 | 176-182 |
| Kouldjinski (DVIT2680) | 303-315 | 290-294 | 243-243 | 249-271 | 179-181 | 241-254 | 233-247 | 165-173 | 77-83 | 149-155 | 143-151 | 172-188 | 165-165 | 210-215 | 353-353 | 228-240 | 256-263 | 151-155 | 172-184 |

| | | | | | | | | | | | | | | | | | | | |
|---|---|---|---|---|---|---|---|---|---|---|---|---|---|---|---|---|---|---|---|
| Kovalewka (DVIT0329) | **319-325** | **294-294** | **235-276** | **249-257** | **179-179** | **247-254** | **239-249** | **165-181** | **79-83** | **155-155** | **133-145** | **186-206** | **151-165** | **206-206** | **353-361** | **238-246** | **263-263** | **149-149** | **188-194** |
| Koz ouzioum (2635Mtp1) | **315-319** | **294-294** | **233-257** | **271-271** | **185-194** | **246-249** | **249-249** | **171-183** | **83-83** | **155-157** | **135-137** | **178-202** | **151-165** | **206-210** | **353-360** | **234-236** | **263-263** | **151-151** | **178-184** |
| Kule Dary (DVIT1070) | **-:-** | **290-294** | **233-257** | **249-271** | **179-179** | **247-247** | **235-245** | **165-183** | **-:-** | **177-177** | **125-153** | **178-182** | **165-165** | **210-214** | **353-353** | **236-240** | **256-256** | **151-151** | **186-190** |
| Kush Dzhumurtka (Turkmn 18820) | **315-328** | **290-294** | **247-271** | **-:-** | **179-187** | **246-254** | **233-233** | **165-165** | **77-83** | **159-173** | **137-155** | **164-182** | **165-165** | **210-215** | **353-353** | **240-240** | **256-263** | **149-151** | **186-190** |
| Kwar II (DVIT2452) | **317-317** | **290-298** | **235-257** | **249-255** | **179-189** | **246-254** | **243-247** | **165-165** | **75-79** | **177-177** | **133-151** | **188-190** | **163-165** | **215-215** | **355-360** | **226-240** | **263-263** | **151-155** | **160-190** |
| Lal Sorkh (DVIT0442) | **319-319** | **290-298** | **243-245** | **249-271** | **179-194** | **249-254** | **239-247** | **165-165** | **79-83** | **173-177** | **145-155** | **172-190** | **163-177** | **215-215** | **353-353** | **234-238** | **261-263** | **149-155** | **178-190** |
| Late Vavilov (ARM Q01-16) | **315-319** | **290-290** | **233-243** | **249-271** | **179-194** | **247-247** | **249-253** | **165-169** | **77-79** | **177-177** | **125-151** | **182-202** | **139-165** | **210-215** | **360-376** | **234-240** | **263-263** | **151-155** | **182-186** |
| Leanoy (DVIT2641) | **315-319** | **288-294** | **239-257** | **243-271** | **-:-** | **246-246** | **249-249** | **171-183** | **79-83** | **149-157** | **135-145** | **172-202** | **165-165** | **206-206** | **353-357** | **228-236** | **263-263** | **151-151** | **178-188** |
| Liali bidona (0Mtp1475) | **317-319** | **290-290** | **217-243** | **249-257** | **185-194** | **246-247** | **239-239** | **165-183** | **79-83** | **173-177** | **145-151** | **188-188** | **165-167** | **206-206** | **355-355** | **226-234** | **256-263** | **147-155** | **178-184** |
| Liali Yakdona (0Mtp640) | **315-319** | **294-294** | **243-243** | **249-257** | **179-185** | **247-254** | **233-249** | **183-193** | **83-83** | **155-155** | **135-135** | **182-182** | **165-165** | **206-210** | **353-360** | **228-236** | **256-263** | **149-155** | **172-190** |
| Lkeni noir (2640Mtp1) | **328-328** | **294-298** | **239-257** | **271-271** | **181-185** | **241-241** | **239-239** | **165-183** | **75-79** | **151-167** | **125-137** | **172-190** | **139-165** | **214-215** | **343-353** | **234-240** | **256-263** | **149-149** | **178-180** |
| Long Yan (0Mtp828) | **319-338** | **290-290** | **243-243** | **257-271** | **193-194** | **241-247** | **233-247** | **165-183** | **77-79** | **149-149** | **143-167** | **164-172** | **165-165** | **214-215** | **353-378** | **234-236** | **263-263** | **151-151** | **170-182** |
| Malahy (0Mtp1449) | **319-319** | **298-306** | **257-270** | **255-271** | **185-194** | **241-247** | **239-247** | **183-183** | **77-79** | **155-173** | **135-135** | **178-213** | **139-151** | **210-214** | **360-360** | **236-246** | **256-265** | **149-151** | **172-182** |
| Mamidon (Turkmn 3025) | **319-328** | **290-298** | **217-247** | **271-271** | **-:-** | **247-254** | **233-251** | **165-165** | **79-83** | **155-159** | **135-153** | **164-188** | **139-147** | **206-206** | **353-357** | **234-240** | **263-263** | **149-151** | **184-190** |
| Mamidon Deli (Turkmn 6984) | **315-328** | **290-290** | **217-257** | **249-271** | **179-179** | **247-254** | **233-251** | **169-193** | **79-83** | **153-177** | **135-153** | **182-188** | **147-151** | **206-206** | **353-353** | **240-240** | **263-263** | **149-149** | **174-186** |
| Mamidon Kizil (Turkmn 21604) | **317-328** | **290-298** | **217-257** | **-:-** | **179-185** | **241-254** | **249-251** | **165-169** | **79-79** | **-:-** | **139-153** | **164-182** | **-:-** | **206-206** | **-:-** | **226-240** | **256-263** | **149-151** | **174-190** |
| Matrassa (0Mtp703) | **315-317** | **294-298** | **234-243** | **257-259** | **179-185** | **241-246** | **233-243** | **173-183** | **81-83** | **149-149** | **125-141** | **172-172** | **165-177** | **206-206** | **360-360** | **-:-** | **256-267** | **149-151** | **186-188** |

| | | | | | | | | | | | | | | | | | | | |
|---|---|---|---|---|---|---|---|---|---|---|---|---|---|---|---|---|---|---|---|
| Matrassa (2642Mtp2) | 315-315 | 288-290 | 239-270 | 243-251 | 181-185 | 241-241 | 249-262 | 183-183 | 77-79 | 159-167 | 135-145 | 186-202 | 151-165 | 206-210 | 357-368 | 228-240 | 256-265 | 151-151 | 178-182 |
| Mehdi 01 (2001-9-8101-01) | -:- | 294-294 | 257-257 | -:- | 179-193 | 241-247 | 233-249 | 165-171 | 75-83 | 159-159 | 135-143 | 178-182 | -:- | 210-215 | -:- | 234-236 | 256-263 | 149-149 | 184-186 |
| Mellei (Turkmn 3030) | 315-319 | 290-294 | 235-247 | 261-271 | 179-185 | 249-254 | 249-253 | 187-193 | 79-79 | 153-176 | 139-153 | 164-188 | 165-165 | 206-210 | 353-353 | 234-234 | 256-256 | 149-149 | 184-190 |
| Mermark (DVIT1042) | 315-319 | 290-306 | 243-257 | 261-271 | 179-179 | 247-247 | 239-249 | 185-185 | 79-79 | 149-149 | 125-143 | 182-214 | 159-167 | 206-218 | -:- | 232-236 | 263-263 | 149-151 | 176-176 |
| Mesisti rose (0Mtp1795) | 320-326 | 290-298 | 243-243 | 257-263 | 181-185 | 246-247 | 233-247 | 165-171 | 79-79 | 149-159 | 135-135 | 202-207 | 151-165 | 206-215 | 347-368 | 228-236 | 263-267 | 151-157 | 188-194 |
| Millishun (DVIT2498) | 315-328 | 290-294 | 235-247 | 271-271 | 179-189 | 241-254 | 249-249 | 173-181 | 83-83 | 153-153 | 141-141 | 186-188 | 139-165 | 206-214 | 358-358 | 234-238 | 263-263 | 149-151 | 174-174 |
| Monaca (1742Mtp1) | 319-319 | 290-290 | 217-234 | 255-257 | 194-194 | 241-247 | 243-245 | 165-165 | 79-83 | 155-155 | 143-151 | 182-190 | 139-165 | 214-218 | 357-360 | 234-234 | 263-263 | 151-155 | 182-186 |
| Monukka (DVIT0462) | 317-319 | 290-290 | 217-243 | 249-255 | 181-194 | 247-247 | 253-253 | 165-183 | 79-79 | 177-177 | 141-151 | 182-188 | 163-165 | 215-215 | 353-375 | 234-240 | 261-263 | 155-155 | 178-184 |
| Mouchketny (0Mtp750) | 315-315 | 294-294 | 257-257 | 263-271 | 179-179 | 253-263 | 243-249 | 165-183 | 77-77 | -:- | 133-133 | 182-205 | 165-165 | 206-215 | 371-371 | 228-228 | 261-263 | 147-147 | 182-182 |
| Mourvedre Famellestadt (DVIT0319) | 315-319 | 290-290 | 243-257 | 239-255 | 179-189 | 241-247 | 249-249 | 171-187 | 83-83 | 161-167 | 133-151 | 178-178 | 165-165 | 206-215 | 353-360 | 226-240 | 263-263 | 151-157 | 178-190 |
| Murma Isium (DVIT0320) | 315-319 | 290-294 | 257-257 | 255-261 | 179-185 | 252-256 | 239-253 | 171-195 | 77-83 | 155-173 | 143-149 | 168-188 | 166-177 | 206-206 | 355-377 | 238-240 | 256-263 | 147-149 | 178-188 |
| Mzivani (TYR VI 15-13) | 311-319 | 288-294 | 235-239 | 261-271 | 179-179 | 241-254 | 249-253 | 165-171 | 77-79 | 157-163 | 135-141 | 166-180 | 139-163 | 206-214 | 345-353 | 234-240 | 263-263 | 149-151 | 178-194 |
| Namonia (DVIT2511) | 289-328 | 290-294 | 233-237 | 255-273 | 179-194 | 247-247 | 239-249 | 165-167 | 75-77 | 153-177 | 145-153 | 166-182 | 139-165 | 206-215 | 355-355 | 234-248 | 256-263 | 151-151 | 168-184 |
| Narma (2648Mtp2) | 303-319 | 290-294 | 235-257 | 243-271 | 183-194 | 249-263 | 245-249 | 183-183 | 79-83 | 159-177 | 135-149 | 178-202 | 139-165 | 206-206 | 353-353 | 234-236 | 263-265 | 151-151 | 176-188 |
| Nassau (0Mtp780) | 303-315 | 290-306 | 235-235 | 239-257 | 189-189 | 247-253 | 239-239 | 165-187 | 77-77 | 149-159 | 143-145 | 166-178 | 165-173 | 206-212 | 353-353 | 240-246 | 263-263 | 149-151 | 176-182 |
| Neeli (DVIT2514) | 315-328 | -:- | 243-247 | 257-257 | 179-194 | -:- | 249-253 | 165-167 | 79-81 | -:- | 135-155 | 182-188 | 165-165 | 206-206 | 357-360 | 232-240 | 263-263 | 147-155 | 172-184 |

| | | | | | | | | | | | | | | | | | | | |
|---|---|---|---|---|---|---|---|---|---|---|---|---|---|---|---|---|---|---|---|
| Neelilay (DVIT2507) | 289-315 | 294-294 | 235-257 | 249-251 | 179-181 | 247-247 | 239-251 | 165-165 | 77-83 | 149-173 | 135-143 | 188-190 | 165-165 | 215-215 | 358-361 | 236-240 | 263-263 | 151-151 | 174-190 |
| Nimrang (TYR VI 15-17) | 319-319 | 290-290 | 234-243 | 249-271 | 185-194 | 247-254 | 243-247 | 165-183 | 77-79 | 155-177 | 143-151 | 172-188 | 165-165 | 206-215 | 353-360 | 228-234 | 256-263 | 151-151 | 172-184 |
| Nimrang rouge (2736Mtp1) | 315-317 | 290-294 | 234-243 | 257-257 | 185-194 | 241-246 | 233-243 | 173-183 | 77-83 | 155-155 | 143-151 | 172-202 | 165-165 | 206-215 | 360-360 | 236-240 | 256-267 | 151-151 | 186-188 |
| Noir D'automne (DVIT0330) | 315-315 | 290-294 | 257-275 | 251-271 | 194-194 | 247-247 | 239-249 | 183-183 | 79-83 | 157-177 | 135-137 | 178-202 | 151-165 | 206-214 | 353-371 | 232-236 | 256-263 | 151-151 | 178-180 |
| Norakert PRG 2224 (DVIT2683) | 319-321 | 298-308 | 239-271 | 261-271 | 179-194 | 241-247 | 239-255 | 183-183 | 77-79 | 149-173 | 125-145 | 182-202 | 163-165 | 214-214 | 353-353 | 234-246 | -:- | -:- | 172-194 |
| Nosargoon (DVIT2533) | 315-317 | 290-290 | 235-243 | 255-273 | 187-194 | 241-247 | 243-253 | 165-183 | 77-83 | 165-167 | 135-151 | 172-202 | 139-165 | 204-206 | 355-360 | 226-240 | 263-263 | 155-155 | 178-178 |
| Noulizok (2649Mtp1) | 315-319 | 290-290 | 233-243 | 257-271 | 185-194 | 241-254 | 249-253 | 165-169 | 79-83 | 149-149 | 135-135 | 182-182 | 139-165 | 206-218 | 356-357 | 234-236 | 256-263 | 147-151 | 174-188 |
| Nunaka Sia (DVIT2045) | 315-317 | 290-290 | 217-259 | 249-271 | 185-194 | 254-264 | 239-249 | 165-165 | 83-83 | 155-159 | 145-151 | 172-190 | 163-165 | 206-206 | 371-371 | 228-234 | 263-263 | 147-151 | 178-182 |
| Oktiabrskii (2854Mtp1) | 319-319 | 290-290 | 234-245 | 249-251 | 185-194 | 241-247 | 243-243 | 165-183 | 77-83 | 157-177 | 135-151 | 172-188 | 165-167 | 206-208 | 353-357 | 232-234 | 256-263 | 149-151 | 184-188 |
| Orion (Turkmn 30748) | 303-315 | 290-294 | 235-235 | 247-271 | 185-189 | 241-254 | 247-253 | 183-193 | 79-79 | 173-177 | 135-139 | 172-188 | 151-165 | 204-210 | 353-353 | 232-234 | 256-263 | 149-151 | 172-190 |
| Otcha bala (2952Mtp1) | 303-317 | 290-294 | 233-243 | 249-255 | 179-194 | 246-254 | 245-253 | 165-193 | 77-77 | 167-177 | 135-151 | 178-182 | 165-165 | 210-210 | 353-353 | 228-234 | 263-263 | 151-155 | 184-188 |
| Ozaan Daii (2075Mtp1) | 315-319 | 290-298 | 235-243 | 249-271 | 181-185 | 247-254 | 239-249 | 171-183 | 77-79 | 155-159 | 135-145 | 168-188 | 165-165 | 206-220 | 353-363 | 234-246 | 261-263 | 147-155 | 182-184 |
| Pakistan Collection 25168 (DVIT2282) | 315-328 | 290-294 | 233-235 | 251-255 | 189-189 | 246-246 | 231-253 | 165-165 | 77-79 | 165-167 | 135-151 | 164-188 | 163-165 | 210-214 | 355-360 | 228-240 | 263-263 | 151-155 | 178-186 |
| Pakistan Collection 25180 (DVIT2269) | -:- | -:- | -:- | -:- | 187-189 | -:- | 231-253 | -:- | -:- | -:- | 135-151 | 164-188 | -:- | -:- | -:- | 228-240 | -:- | -:- | -:- |
| Pakistan Collection 25237 (DVIT2272) | 315-317 | 290-294 | 235-243 | 251-255 | 185-194 | 247-247 | 251-253 | 165-173 | 77-79 | 149-165 | 135-151 | 188-202 | 139-165 | 206-214 | 357-360 | 232-240 | 263-263 | 149-155 | 178-194 |
| Pakistan Collection 25241 (DVIT2271) | 315-319 | 290-294 | 243-247 | 255-255 | 179-194 | 246-246 | 249-251 | 169-173 | 77-79 | 165-167 | 135-135 | 164-188 | 139-165 | 206-214 | 357-360 | 232-234 | 263-263 | 151-155 | 186-194 |
| Pakistan Collection 25265 (DVIT2293) | 289-319 | 290-294 | 235-247 | 257-271 | 189-189 | 241-254 | 249-249 | 173-181 | 83-83 | 173-173 | 135-141 | 172-182 | 139-139 | 206-206 | 353-353 | 228-246 | 256-263 | 151-151 | 172-174 |

| | | | | | | | | | | | | | | | | | | | |
|---|---|---|---|---|---|---|---|---|---|---|---|---|---|---|---|---|---|---|---|
| Pakistan Collection 25275 (DVIT2263) | **319-328** | **290-306** | **235-247** | **243-257** | **185-189** | **241-246** | **249-249** | **-:-** | **77-83** | **141-159** | **131-133** | **164-190** | **151-165** | **210-214** | **347-353** | **226-246** | **263-263** | **151-151** | **166-190** |
| Pakistan Collection 25290 (DVIT2296) | **289-328** | **290-290** | **233-235** | **257-271** | **179-179** | **247-254** | **249-249** | **165-185** | **81-83** | **141-153** | **125-141** | **182-182** | **165-165** | **204-214** | **347-347** | **228-234** | **263-263** | **147-151** | **178-194** |
| Pakistan Collection 25296 (DVIT2283) | **289-317** | **294-306** | **235-243** | **251-261** | **181-181** | **247-254** | **243-251** | **165-173** | **79-79** | **165-173** | **135-151** | **182-202** | **141-165** | **214-214** | **355-361** | **232-248** | **263-265** | **-:-** | **178-190** |
| Pakistan Collection 25311 (DVIT2264) | **315-317** | **-:-** | **235-243** | **243-255** | **179-185** | **247-247** | **239-253** | **165-173** | **77-83** | **149-165** | **147-151** | **172-202** | **141-165** | **204-206** | **353-353** | **232-240** | **263-263** | **149-151** | **172-194** |
| Parargoon (DVIT2502) | **317-328** | **290-294** | **233-243** | **243-255** | **179-185** | **246-246** | **251-251** | **165-169** | **77-83** | **149-159** | **141-151** | **182-188** | **139-165** | **206-210** | **355-360** | **226-240** | **263-263** | **149-149** | **172-194** |
| Parkent (2691Mtp1) | **313-319** | **290-294** | **243-243** | **257-271** | **183-194** | **247-247** | **233-247** | **165-183** | **79-83** | **155-177** | **143-151** | **172-172** | **165-177** | **206-215** | **353-353** | **228-236** | **256-256** | **149-151** | **184-186** |
| Peikani (2597Mtp1) | **319-329** | **294-298** | **243-257** | **271-271** | **183-194** | **241-247** | **239-239** | **169-173** | **79-83** | **163-177** | **137-141** | **174-174** | **163-165** | **206-206** | **353-353** | **236-240** | **263-263** | **147-151** | **178-190** |
| Persian R27 (DVIT2755) | **317-317** | **294-294** | **225-243** | **247-271** | **183-185** | **247-247** | **241-249** | **169-175** | **79-79** | **155-167** | **123-133** | **166-172** | **163-163** | **206-206** | **346-360** | **232-232** | **263-263** | **149-155** | **174-184** |
| Pervenetz Praskoveisky (2651Mtp2) | **317-328** | **290-294** | **239-257** | **243-261** | **179-181** | **247-253** | **249-259** | **167-193** | **83-83** | **167-167** | **125-145** | **-:-** | **165-165** | **208-210** | **353-368** | **234-240** | **256-267** | **151-151** | **172-188** |
| Pinger putao (2788Mtp1) | **319-338** | **290-290** | **243-257** | **271-271** | **179-194** | **247-247** | **239-247** | **165-173** | **79-79** | **145-145** | **143-155** | **172-202** | **139-165** | **210-215** | **353-357** | **236-236** | **263-263** | **151-151** | **170-184** |
| Porsi Shekerek (Turkmn 19735) | **315-315** | **290-294** | **243-257** | **271-271** | **-:-** | **241-254** | **243-245** | **171-193** | **79-83** | **159-177** | **137-155** | **178-190** | **165-165** | **206-210** | **-:-** | **-:-** | **256-263** | **151-151** | **184-190** |
| Precoce D'astrachan (DVIT0321) | **-:-** | **-:-** | **257-274** | **-:-** | **185-194** | **-:-** | **245-249** | **165-183** | **79-83** | **157-177** | **135-137** | **186-202** | **-:-** | **210-214** | **-:-** | **228-236** | **263-263** | **147-151** | **180-184** |
| Précoce d'Astrakan (0Mtp928) | **315-319** | **294-298** | **257-274** | **255-271** | **185-194** | **246-247** | **245-249** | **165-183** | **79-83** | **157-177** | **135-137** | **186-202** | **151-165** | **210-214** | **353-373** | **228-236** | **263-263** | **147-151** | **180-184** |
| Rajoulan (0Mtp950) | **319-319** | **290-294** | **243-257** | **251-271** | **-:-** | **254-264** | **247-249** | **165-183** | **79-79** | **163-171** | **143-151** | **174-182** | **159-167** | **206-215** | **353-371** | **226-228** | **263-263** | **149-149** | **174-182** |
| Ranny Vira (2654Mtp1) | **319-319** | **290-298** | **216-257** | **249-271** | **181-183** | **253-253** | **249-249** | **165-183** | **79-79** | **167-171** | **151-151** | **182-202** | **159-163** | **204-218** | **353-356** | **234-238** | **261-263** | **147-151** | **178-182** |
| Razakiia piembiana (2737Mtp1) | **315-315** | **290-294** | **233-243** | **271-271** | **181-185** | **241-246** | **239-249** | **173-183** | **79-79** | **165-177** | **135-145** | **186-207** | **163-175** | **206-206** | **353-368** | **236-246** | **263-263** | **149-151** | **178-178** |

| | | | | | | | | | | | | | | | | | | | |
|---|---|---|---|---|---|---|---|---|---|---|---|---|---|---|---|---|---|---|---|
| Raziki (1750Mtp1) | **315-319** | **290-306** | **243-253** | **255-255** | **179-179** | **247-254** | **239-251** | **171-183** | **83-83** | **149-173** | **133-135** | **182-208** | **165-165** | **214-215** | **368-371** | **226-238** | **261-263** | **149-151** | **176-178** |
| Red Ohanez (DVIT0499) | **315-323** | **294-294** | **233-233** | **257-271** | **185-185** | **241-254** | **239-249** | **183-187** | **79-83** | **147-159** | **135-145** | **178-188** | **165-165** | **206-215** | **357-357** | **226-232** | **263-263** | **149-149** | **172-174** |
| Rhazaki (Pa 1882) (TYR VI 16-09) | **315-315** | **290-294** | **233-257** | **257-271** | **183-185** | **254-254** | **239-249** | **183-183** | **77-79** | **147-159** | **133-135** | **166-188** | **165-177** | **206-206** | **353-357** | **228-234** | **263-263** | **-:-** | **172-182** |
| Rhazaki Anatolico (DVIT0608) | **315-328** | **290-294** | **227-243** | **271-271** | **179-194** | **254-264** | **247-253** | **165-169** | **77-83** | **149-155** | **135-151** | **164-202** | **159-165** | **206-206** | **355-368** | **234-236** | **263-263** | **151-151** | **174-184** |
| Rhazaki De Crete (DVIT0501) | **315-315** | **290-294** | **233-257** | **257-271** | **179-194** | **254-254** | **243-249** | **183-183** | **77-79** | **147-159** | **133-135** | **166-188** | **165-177** | **206-206** | **353-357** | **226-238** | **-:-** | **149-149** | **172-182** |
| Rhazaki Mavro (DVIT0555) | **319-319** | **290-290** | **243-259** | **257-271** | **181-185** | **247-264** | **249-249** | **165-183** | **77-83** | **157-173** | **133-151** | **188-206** | **167-173** | **206-210** | **353-361** | **228-234** | **263-263** | **149-151** | **174-194** |
| Rish Baba (HOP L04-19) | **315-319** | **290-290** | **243-257** | **249-271** | **194-194** | **247-247** | **243-243** | **165-173** | **79-79** | **149-149** | **147-155** | **182-202** | **139-177** | **210-214** | **353-360** | **224-234** | **263-267** | **155-155** | **182-184** |
| Rizamat (DVIT2338) | **319-319** | **290-294** | **233-243** | **255-257** | **179-194** | **247-249** | **233-253** | **165-183** | **79-83** | **153-177** | **151-155** | **172-182** | **177-177** | **215-218** | **353-353** | **228-240** | **256-263** | **149-155** | **178-184** |
| Rouchaki (2783Mtp1) | **315-315** | **290-298** | **217-235** | **249-271** | **179-181** | **241-247** | **239-247** | **165-183** | **79-83** | **173-177** | **151-151** | **178-188** | **163-177** | **206-210** | **353-353** | **234-238** | **261-263** | **147-155** | **182-182** |
| Sabza angur (0Mtp1007) | **315-319** | **290-294** | **233-243** | **271-273** | **185-194** | **247-254** | **239-249** | **165-169** | **83-83** | **155-167** | **151-155** | **182-202** | **139-165** | **206-218** | **356-356** | **234-240** | **263-263** | **147-151** | **184-188** |
| Sahami (2090Mtp1) | **319-319** | **294-294** | **245-270** | **271-271** | **179-194** | **241-264** | **239-249** | **169-181** | **77-77** | **149-149** | **133-151** | **202-202** | **159-165** | **210-214** | **356-357** | **238-246** | **263-263** | **147-151** | **172-182** |
| Sahebi (0Mtp587) | **317-319** | **290-294** | **234-234** | **249-257** | **179-194** | **241-241** | **243-253** | **165-183** | **79-83** | **149-149** | **135-151** | **172-182** | **139-151** | **206-214** | **360-375** | **240-240** | **256-263** | **151-155** | **186-188** |
| Sahibi (DVIT0509) | **317-317** | **290-294** | **245-259** | **257-271** | **194-194** | **247-249** | **247-253** | **165-173** | **79-79** | **155-173** | **141-145** | **172-182** | **139-163** | **215-215** | **353-353** | **238-240** | **261-263** | **151-155** | **188-194** |
| Sahibi Sorkh (DVIT0510) | **317-317** | **294-298** | **243-257** | **249-271** | **179-179** | **247-249** | **235-247** | **165-165** | **79-83** | **159-177** | **135-145** | **172-182** | **165-177** | **206-215** | **353-361** | **238-240** | **-:-** | **151-155** | **178-186** |
| Sahilii (2078Mtp1) | **315-319** | **290-298** | **243-247** | **255-271** | **179-185** | **247-247** | **239-247** | **165-183** | **77-83** | **173-173** | **135-135** | **186-202** | **165-165** | **206-214** | **343-371** | **246-246** | **263-263** | **151-151** | **178-184** |
| Saïd guliami (0Mtp1010) | **319-319** | **290-290** | **233-257** | **249-271** | **194-194** | **246-247** | **243-249** | **165-165** | **79-79** | **149-159** | **151-155** | **176-202** | **139-177** | **206-214** | **347-353** | **234-234** | **263-267** | **151-155** | **182-190** |
| Salomani (DVIT0597) | **315-315** | **-:-** | **235-243** | **249-255** | **185-193** | **246-250** | **239-249** | **-:-** | **79-79** | **149-173** | **133-143** | **-:-** | **139-165** | **206-210** | **357-357** | **230-230** | **256-263** | **151-155** | **176-190** |

| | | | | | | | | | | | | | | | | | | | |
|---|---|---|---|---|---|---|---|---|---|---|---|---|---|---|---|---|---|---|---|
| Sary Aygyr (Turkmn 6987) | 315-315 | 290-294 | 233-247 | 251-271 | 179-185 | 247-247 | 235-249 | 165-165 | 79-79 | 149-177 | 125-153 | 186-188 | 165-165 | 210-210 | 353-355 | 236-240 | 256-263 | 151-151 | 178-186 |
| Sary Kiriak (0Mtp1031) | 315-319 | 288-290 | 233-257 | 271-271 | 181-189 | 241-255 | 239-259 | 165-193 | 79-79 | 155-159 | 133-145 | 172-174 | 165-165 | 204-215 | 343-353 | 226-228 | 263-263 | 149-151 | 180-186 |
| Sateni tcherny (388Mtp2) | 315-317 | 290-298 | 217-257 | 249-249 | 181-194 | 247-247 | 239-243 | 165-165 | 79-83 | -:- | -:- | 188-202 | 163-177 | 206-214 | 360-360 | 224-234 | 263-267 | 155-155 | 178-184 |
| Schiradzouli violet (1737Mtp1) | 315-317 | 294-306 | 235-235 | 249-271 | 185-189 | 241-254 | 239-249 | 165-173 | 79-83 | 149-155 | 133-145 | 174-178 | 163-165 | 204-215 | 353-363 | 236-240 | 263-263 | 149-151 | 178-194 |
| Schwin (DVIT2604) | 317-328 | 290-290 | 243-245 | 255-271 | 179-179 | 247-247 | 243-249 | 165-165 | 77-83 | 149-167 | 145-151 | 188-190 | 163-165 | 206-210 | 347-353 | 234-236 | 263-263 | 149-151 | 172-196 |
| Selection 2798 (DVIT1152) | -:- | 294-306 | 217-233 | 249-271 | 181-185 | 247-254 | 249-249 | 183-185 | 78-83 | 155-159 | 133-135 | 166-202 | 163-165 | 206-210 | 353-357 | 226-234 | 263-263 | 149-155 | 172-182 |
| Shahani 01 (2001-9-7097-01) | 315-319 | 294-294 | 235-235 | 257-271 | 179-179 | 247-247 | 247-249 | 181-183 | 77-79 | 149-159 | 135-145 | 178-202 | 165-175 | 214-220 | 357-362 | 224-234 | 263-263 | 151-151 | 180-182 |
| Shakar Angur (TYR VI 16-21) | 317-317 | 290-294 | 243-243 | 249-259 | 179-194 | 241-254 | 247-253 | 165-183 | 77-79 | 149-149 | 137-141 | 172-182 | 165-165 | 210-215 | 353-360 | 234-236 | 256-263 | 151-155 | 172-188 |
| Shingargoon (DVIT2603) | 315-315 | 290-306 | 235-247 | 247-255 | 185-185 | 246-246 | 249-249 | 169-183 | 77-83 | 153-159 | 133-145 | 164-172 | 165-165 | 206-210 | -:- | 226-234 | 263-263 | -:- | 172-190 |
| Shirazi (2079Mtp1) | 315-317 | 298-298 | 235-243 | 249-271 | 181-194 | 247-247 | 247-253 | 165-173 | 79-79 | 155-167 | 141-145 | 182-188 | 165-165 | 206-214 | 353-353 | 234-238 | 263-263 | 151-151 | 182-184 |
| Shtur Angur (DVIT2168) | 315-319 | 290-290 | 227-233 | 255-271 | 181-194 | 254-256 | 249-253 | 169-183 | 77-77 | 155-155 | 143-143 | 166-178 | 177-187 | 206-206 | 360-360 | 236-238 | 263-263 | 149-149 | 174-190 |
| Siah (2074Mtp1) | 319-319 | 290-298 | 235-243 | 255-271 | 181-185 | 247-247 | 239-239 | 165-165 | 79-83 | 159-173 | 135-141 | 182-190 | 139-177 | 206-210 | 353-353 | 238-240 | 263-263 | 147-149 | 182-182 |
| Siborskory (2681Mtp1) | 315-319 | 294-294 | 233-257 | 261-263 | 179-181 | 255-255 | 239-239 | 165-181 | 79-83 | 155-159 | 133-143 | 166-207 | 151-165 | 206-206 | 353-377 | 228-238 | 263-263 | 149-149 | 158-194 |
| Sochal (DVIT1126) | 315-317 | 294-298 | 243-245 | 257-271 | 179-185 | 247-247 | 243-249 | 169-193 | 77-79 | 167-177 | 125-151 | 164-202 | 139-165 | -:- | 353-353 | 234-238 | 256-263 | 151-155 | 178-182 |
| Soïaki (2657Mtp1) | 319-319 | 290-290 | 243-243 | 243-257 | 181-185 | 241-247 | 233-243 | 165-165 | 83-83 | 167-177 | 135-141 | 172-182 | 139-151 | 206-214 | 347-353 | 228-240 | 256-263 | 149-151 | 188-194 |
| Soultani (0Mtp1071) | 319-319 | 290-290 | 243-243 | 271-273 | 194-194 | 247-247 | 239-247 | 165-165 | 79-79 | 149-149 | 151-155 | 182-190 | 159-165 | 206-215 | 353-375 | 240-240 | 263-263 | 147-155 | 182-186 |
| Sourkhak biely (2856Mtp1) | 313-315 | 290-298 | 243-245 | 271-271 | 185-194 | 249-254 | 243-243 | 169-193 | 79-79 | 167-167 | 135-141 | 182-202 | 165-177 | 215-218 | 353-356 | 238-240 | 263-267 | 147-149 | 184-188 |

| | | | | | | | | | | | | | | | | | | | |
|---|---|---|---|---|---|---|---|---|---|---|---|---|---|---|---|---|---|---|---|
| Starinky (0Mtp1073) | **315-319** | **290-294** | **235-257** | **249-251** | **179-189** | **241-255** | **239-247** | **165-195** | **77-79** | **159-165** | **143-145** | **168-182** | **151-165** | **206-210** | **351-377** | **228-240** | **263-263** | **149-149** | **188-190** |
| Sultanine noire faux (0Mtp1597) | **315-319** | **290-290** | **217-234** | **249-249** | **181-194** | **247-254** | **243-249** | **173-183** | **79-83** | **149-155** | **125-151** | **190-202** | **163-177** | **206-218** | **356-360** | **226-234** | **256-267** | **147-155** | **182-182** |
| Sumbarskii Bekyi (Turkmn 6304) | **319-328** | **290-294** | **233-233** | **271-271** | **179-185** | **247-247** | **235-249** | **165-193** | **79-83** | **149-177** | **135-153** | **186-188** | **165-167** | **206-206** | **353-360** | **234-234** | **256-263** | **151-151** | **186-190** |
| Tagobi (2659Mtp1) | **319-327** | **290-290** | **243-243** | **257-257** | **179-185** | **241-254** | **245-253** | **165-173** | **77-83** | **155-155** | **125-143** | **172-202** | **165-177** | **206-214** | **353-357** | **228-234** | **256-263** | **151-151** | **172-182** |
| Taifi (DVIT2174) | **319-319** | **290-294** | **243-243** | **249-257** | **179-194** | **241-254** | **243-247** | **165-183** | **77-79** | **149-155** | **137-143** | **172-182** | **165-165** | **210-215** | **353-360** | **228-234** | **256-263** | **151-155** | **172-182** |
| Taka Sago (TYR VI 17-03) | **315-319** | **288-290** | **225-225** | **239-271** | **179-181** | **238-247** | **235-249** | **165-179** | **79-79** | **145-157** | **133-151** | **170-172** | **165-165** | **206-210** | **360-360** | **236-236** | **263-263** | **155-155** | **174-186** |
| Tana-Kuzi (DVIT2928) | **319-319** | **290-298** | **237-243** | **243-249** | **179-194** | **246-254** | **243-253** | **165-165** | **79-83** | **149-155** | **125-151** | **188-188** | **165-165** | **210-215** | **353-353** | **234-240** | **263-263** | **151-155** | **182-186** |
| Tarnau (TYR VI 17-07) | **319-319** | **290-290** | **243-243** | **249-271** | **-:-** | **247-254** | **247-249** | **156-160** | **79-79** | **155-159** | **143-151** | **188-188** | **163-165** | **210-215** | **353-371** | **228-228** | **256-263** | **151-155** | **184-194** |
| Tavkveri (1218Mtp1) | **315-315** | **288-290** | **234-257** | **249-271** | **185-193** | **241-246** | **239-247** | **165-173** | **77-83** | **155-167** | **135-145** | **166-186** | **139-165** | **204-206** | **355-368** | **228-240** | **263-267** | **151-155** | **178-178** |
| Tchatyrbac (664Mtp1) | **315-315** | **294-294** | **257-257** | **249-255** | **185-187** | **247-254** | **249-249** | **173-187** | **83-83** | **155-177** | **137-145** | **182-188** | **165-175** | **206-215** | **355-361** | **236-238** | **263-263** | **149-151** | **178-182** |
| Tchilar (DVIT2688) | **309-319** | **-:-** | **276-280** | **265-271** | **179-185** | **-:-** | **249-253** | **181-183** | **77-79** | **173-173** | **133-135** | **178-190** | **151-165** | **210-214** | **355-368** | **236-236** | **256-267** | **151-151** | **186-190** |
| Tchiliaki belyi (2671Mtp1) | **313-319** | **290-294** | **237-243** | **249-257** | **179-194** | **241-247** | **233-247** | **165-165** | **79-79** | **149-167** | **141-151** | **172-182** | **139-165** | **214-215** | **361-375** | **234-236** | **256-263** | **151-155** | **182-186** |
| Tolstokory (1985Mtp2) | **315-319** | **294-294** | **239-257** | **243-271** | **181-185** | **246-247** | **249-249** | **171-183** | **83-83** | **149-157** | **135-135** | **172-202** | **165-165** | **206-210** | **353-368** | **228-236** | **263-263** | **151-151** | **178-184** |
| Torgoon (DVIT2531) | **-:-** | **290-306** | **-:-** | **255-273** | **185-194** | **241-247** | **249-253** | **169-183** | **-:-** | **153-165** | **135-135** | **164-172** | **165-165** | **206-206** | **355-360** | **226-232** | **263-263** | **149-151** | **174-178** |
| Trapanlarin kara S1 (0000-0-2411-S1) | **315-315** | **290-294** | **235-257** | **251-271** | **181-183** | **254-254** | **249-253** | **169-189** | **77-79** | **157-159** | **147-155** | **174-186** | **165-167** | **206-210** | **372-374** | **236-246** | **263-263** | **149-151** | **174-174** |
| Tscharma (0Mtp1148) | **315-319** | **290-294** | **243-247** | **249-271** | **179-185** | **246-247** | **247-253** | **165-171** | **77-83** | **155-159** | **135-141** | **174-188** | **165-165** | **206-210** | **353-353** | **226-234** | **256-256** | **151-151** | **178-184** |
| Tufachi S1 (1985-0-2415-S1) | **315-323** | **290-294** | **243-243** | **249-271** | **179-179** | **241-247** | **233-247** | **165-169** | **79-79** | **155-157** | **143-151** | **188-202** | **165-165** | **206-210** | **371-371** | **234-246** | **-:-** | **147-151** | **176-190** |

| | | | | | | | | | | | | | | | | | | | |
|---|---|---|---|---|---|---|---|---|---|---|---|---|---|---|---|---|---|---|---|
| Tuia-tiche (2760Mtp1) | **317-319** | **290-298** | **235-243** | **249-257** | **185-194** | **241-247** | **243-249** | **165-193** | **77-79** | **-:-** | **135-151** | **182-190** | **139-165** | **206-214** | **353-360** | **234-240** | **263-263** | **151-155** | **186-186** |
| Tuia-tiche p.e. (654Mtp1) | **315-319** | **290-294** | **235-257** | **249-271** | **181-187** | **247-253** | **249-249** | **165-169** | **79-79** | **157-173** | **143-149** | **186-207** | **165-165** | **206-206** | **357-371** | **228-248** | **263-263** | **151-151** | **172-188** |
| Uzbekistan Muscat (TYR VI 17-11) | **303-319** | **288-290** | **233-245** | **255-271** | **181-189** | **247-247** | **239-257** | **169-183** | **83-83** | **159-163** | **133-151** | **172-172** | **163-165** | **206-210** | **353-353** | **222-232** | **261-263** | **149-151** | **178-180** |
| Uzbekistanian Muscat (DVIT2072) | **319-319** | **294-298** | **233-243** | **255-263** | **179-179** | **247-264** | **251-253** | **183-193** | **77-79** | **171-177** | **133-155** | **172-204** | **165-175** | **210-210** | **375-375** | **232-240** | **263-263** | **149-149** | **178-186** |
| Varuschkin (0Mtp1165) | **319-319** | **290-294** | **247-257** | **251-251** | **185-194** | **241-249** | **245-245** | **183-187** | **83-83** | **159-171** | **133-143** | **186-202** | **165-165** | **206-206** | **353-363** | **236-238** | **263-263** | **149-151** | **178-194** |
| Vassarga bielaia (126Mtp2) | **313-315** | **290-290** | **245-245** | **255-257** | **183-185** | **247-254** | **233-243** | **165-183** | **83-83** | **145-167** | **125-143** | **174-182** | **151-165** | **206-214** | **357-361** | **236-240** | **263-267** | **151-155** | **184-188** |
| Vassarga tchernaia (2510Mtp1) | **315-319** | **290-294** | **233-243** | **249-271** | **179-194** | **247-247** | **249-249** | **165-169** | **77-77** | **155-177** | **125-137** | **164-190** | **139-165** | **206-214** | **353-360** | **234-240** | **256-263** | **151-151** | **182-182** |
| *Vitis Vinifera* #1359 (TYR VI 17-15) | **319-325** | **290-298** | **243-267** | **271-271** | **181-181** | **249-264** | **233-247** | **187-193** | **79-83** | **153-159** | **141-155** | **168-182** | **165-165** | **210-215** | **353-368** | **236-238** | **263-263** | **147-149** | **184-192** |
| Voivoginiova S1 (1983-0-2424-S1) | **319-323** | **290-290** | **257-267** | **251-251** | **181-185** | **254-264** | **249-249** | **183-183** | **83-83** | **155-173** | **135-143** | **168-188** | **165-165** | **206-206** | **353-371** | **226-236** | **263-263** | **157-157** | **178-178** |
| Volgo Don (TYR VI 17-17) | **315-315** | **290-298** | **233-257** | **255-271** | **194-194** | **247-249** | **239-253** | **165-165** | **77-77** | **153-157** | **151-155** | **172-182** | **165-177** | **210-210** | **353-375** | **226-240** | **263-263** | **-:-** | **178-188** |
| Yaghotti No. 1 (DVIT0604) | **315-315** | **290-298** | **243-257** | **249-271** | **185-193** | **254-254** | **249-253** | **171-173** | **83-83** | **167-177** | **143-143** | **182-186** | **165-167** | **215-215** | **353-353** | **224-240** | **263-267** | **151-155** | **182-194** |
| Yaï izium rose (2687Mtp1) | **315-317** | **294-298** | **257-259** | **247-269** | **181-185** | **249-253** | **247-259** | **165-171** | **83-83** | **159-179** | **125-135** | **172-186** | **-:-** | **206-210** | **353-361** | **234-240** | **256-263** | **151-151** | **186-188** |
| Yarghouti (TYR VI 17-19) | **315-317** | **290-298** | **217-243** | **249-271** | **181-185** | **247-254** | **243-253** | **173-183** | **79-83** | **155-177** | **143-145** | **186-190** | **165-165** | **206-215** | **353-360** | **234-240** | **261-267** | **151-155** | **178-194** |
| Yhsouh ali (2077Mtp1) | **315-317** | **290-294** | **235-247** | **251-257** | **179-185** | **241-247** | **249-249** | **165-183** | **77-83** | **147-159** | **135-141** | **178-188** | **139-165** | **214-214** | **353-357** | **226-246** | **256-256** | **151-151** | **172-182** |
| Yumalak lelyi (2679Mtp1) | **299-319** | **290-290** | **233-243** | **243-251** | **185-194** | **241-247** | **243-249** | **165-165** | **77-83** | **145-173** | **135-151** | **182-182** | **165-165** | **206-210** | **353-360** | **228-228** | **256-263** | **149-151** | **172-184** |
| Zerk (DVIT0606) | **315-323** | **294-306** | **243-243** | **255-255** | **179-185** | **247-247** | **247-249** | **187-193** | **83-83** | **-:-** | **143-153** | **182-202** | **139-139** | **206-210** | **353-353** | **226-230** | **256-263** | **149-151** | **172-186** |

| | | | | | | | | | | | | | | | | | | | |
|---|---|---|---|---|---|---|---|---|---|---|---|---|---|---|---|---|---|---|---|
| Zimliansky noir (2666Mtp1) | 315-315 | 290-294 | 235-257 | 251-261 | 187-194 | 241-247 | 239-249 | 187-195 | 77-83 | 159-167 | 145-145 | 172-207 | 165-165 | 206-206 | 351-353 | 228-240 | 256-263 | 149-151 | 182-190 |
| Zimsko Belo (DVIT2664) | 323-323 | 290-294 | 257-257 | 249-249 | 181-185 | 254-254 | 243-247 | 187-187 | 79-79 | 157-167 | 135-143 | 166-188 | 165-177 | 208-210 | 357-357 | 234-246 | 263-263 | 149-151 | 182-188 |
| Arybata (DVIT2442.1) | 313-313 | 290-294 | 233-235 | 249-271 | 185-189 | 241-248 | 249-249 | 165-165 | 79-83 | -:- | 133-151 | 178-186 | 139-139 | 208-210 | 343-360 | 232-234 | 256-267 | 151-151 | 178-178 |
| Arybata (DVIT2442.6) | -:- | 290-294 | 242-251 | 247-249 | 185-185 | 241-241 | 249-249 | 183-183 | 77-83 | 155-155 | 125-133 | 178-186 | 139-163 | 210-210 | 353-368 | 226-240 | 256-256 | 151-151 | 182-182 |
| Ayedere (DVIT2440.1) | 299-317 | 294-294 | 243-245 | 249-249 | 179-189 | 247-254 | 235-235 | 192-192 | 77-77 | 147-167 | 133-135 | 164-190 | 147-151 | 206-214 | 353-353 | 230-234 | 256-263 | 147-151 | 174-174 |
| Ayedere (DVIT2440.9) | -:- | -:- | 243-247 | -:- | 185-185 | -:- | 235-245 | 165-165 | 77-79 | 147-147 | 133-135 | 186-188 | 139-151 | 206-210 | -:- | 234-234 | 256-263 | 149-151 | 186-188 |
| Kara Kaltak (2690Mtp1) | 319-327 | 290-290 | 243-243 | 249-257 | 179-185 | 241-254 | 233-253 | 165-165 | 77-79 | 155-177 | 143-143 | 172-182 | 165-165 | 210-214 | 353-360 | 228-240 | 263-263 | 151-155 | 172-184 |
| Kara Kaytak (DVIT2445.12) | 328-328 | 294-294 | 233-243 | 247-247 | 179-179 | 247-254 | 259-261 | 165-165 | 79-79 | 147-147 | 139-141 | 202-202 | 139-139 | 204-218 | 368-368 | 234-234 | 256-256 | 151-151 | 188-188 |
| Kochtemyr (DVIT2444.19) | 319-319 | 294-294 | 243-276 | 255-255 | -:- | 247-247 | 253-253 | 183-183 | 81-81 | 149-149 | 145-145 | 166-184 | 139-139 | 206-210 | 343-343 | -:- | 256-263 | 151-151 | 174-190 |
| Uzuntakoy (DVIT2447.4) | 313-313 | 294-294 | 245-245 | 249-261 | 179-185 | 247-247 | 245-245 | 185-185 | 79-83 | 167-167 | 125-135 | 166-176 | 151-151 | 210-210 | 353-353 | 234-236 | 263-263 | 151-151 | 186-188 |
| Uzuntakoy (DVIT2447.9) | 313-319 | 294-294 | 233-245 | 261-271 | 179-185 | 247-247 | 245-247 | 185-185 | 79-79 | 167-173 | 133-141 | 176-202 | 139-139 | -:- | 347-353 | 232-236 | 263-263 | 151-151 | 186-188 |
| Yuvankala (DVIT2446.9) | 315-319 | 290-290 | 233-245 | 247-249 | 185-189 | 247-247 | 249-249 | 165-173 | 77-83 | 149-155 | 149-153 | 170-188 | 165-165 | 208-210 | 353-353 | 232-234 | 263-263 | 147-149 | 192-196 |
| Yuvankala (DVIT2446.12) | 313-313 | 290-294 | 243-245 | 249-261 | 179-183 | 247-247 | 245-253 | 185-193 | 79-79 | 167-167 | 125-135 | 178-188 | 151-151 | 206-210 | 353-353 | 234-236 | 263-263 | 151-151 | 172-188 |
| O30-44 (DVIT1798) | -:- | -:- | 235-235 | 253-271 | 179-179 | -:- | 233-255 | 173-185 | 83-83 | 149-173 | 133-133 | 202-202 | 139-139 | 204-208 | 355-355 | 238-246 | 256-263 | 147-147 | 158-158 |
| O30-51 (DVIT1799) | -:- | 290-294 | 235-277 | 253-271 | 179-185 | -:- | 233-255 | 173-185 | 81-83 | 149-173 | 133-133 | 202-202 | 139-139 | 204-208 | 355-355 | 238-246 | 256-263 | 147-147 | 158-178 |
| O30-53 (DVIT1800) | -:- | -:- | 217-251 | 249-253 | 179-179 | 247-247 | 243-255 | 165-185 | 79-81 | 167-181 | 133-137 | 188-202 | 139-175 | 204-206 | 358-358 | 234-246 | 256-267 | 147-151 | 158-182 |
| O33-60 (DVIT1802) | -:- | 294-294 | 235-251 | 245-253 | 179-193 | -:- | 243-253 | 165-173 | 79-79 | 159-181 | 133-137 | 177-177 | 139-179 | 204-210 | 353-353 | 234-234 | 256-267 | 147-151 | 178-186 |

| | | | | | | | | | | | | | | | | | | | |
|---|---|---|---|---|---|---|---|---|---|---|---|---|---|---|---|---|---|---|---|
| O34-16 (DVIT1803) | -:- | 290-294 | 247-277 | 249-249 | 179-193 | -:- | 247-255 | 165-185 | 81-81 | 149-159 | 133-133 | 178-178 | 139-175 | 204-208 | 353-353 | 228-246 | 263-267 | 147-151 | 174-178 |
| O34-29 (DVIT1804) | -:- | 294-294 | 235-277 | 247-249 | 185-193 | 241-247 | 243-247 | 165-183 | 79-83 | 173-181 | 133-133 | 177-177 | 139-175 | 204-218 | 355-360 | 234-246 | 256-263 | 147-147 | 158-186 |
| O34-46 (DVIT1805) | -:- | 288-294 | 234-253 | 253-261 | 183-190 | 241-254 | 239-263 | 171-183 | 77-77 | 149-149 | 141-147 | 177-206 | 139-147 | 206-206 | 353-371 | 228-238 | 256-267 | 151-151 | 176-182 |
| O34-54 (DVIT1806) | -:- | 294-294 | 234-251 | 251-261 | 175-190 | 247-254 | 239-263 | 165-183 | 77-83 | 149-149 | 141-141 | 177-206 | 139-147 | 206-206 | 368-371 | 228-236 | 256-263 | 149-151 | 182-190 |
| O34-55 (DVIT1807) | -:- | 288-290 | 234-253 | 251-261 | 175-183 | -:- | 235-239 | 171-183 | 79-83 | 149-149 | 141-147 | 181-188 | 139-159 | 208-208 | 368-371 | 228-236 | 256-263 | 149-151 | 182-190 |
| O35-07 (DVIT1808) | -:- | -:- | 253-253 | 251-261 | 190-190 | -:- | 235-263 | 171-183 | 77-79 | 149-167 | 141-147 | 181-188 | 139-159 | 206-210 | 368-368 | 228-236 | 256-263 | 149-151 | 178-182 |
| O35-11 (DVIT1809) | -:- | -:- | 253-253 | 253-261 | 183-190 | -:- | 263-263 | -:- | 77-79 | 149-149 | 141-147 | 177-206 | 147-159 | 206-210 | 357-368 | 228-228 | 256-267 | 149-151 | -:- |
| O35-41 (DVIT1811) | -:- | -:- | 235-247 | 247-249 | 189-194 | -:- | 235-247 | 165-183 | 79-83 | 149-149 | 151-151 | 181-188 | 139-139 | 206-210 | 353-368 | 228-234 | 256-263 | 149-149 | 174-190 |
| O35-47 (DVIT1812) | -:- | 290-290 | 235-243 | 247-257 | 189-194 | 248-248 | 247-267 | 169-173 | 79-79 | 149-149 | 135-151 | 182-202 | 147-147 | 206-214 | 353-371 | 228-234 | 263-263 | 147-149 | -:- |
| O35-50 (DVIT1813) | -:- | 290-290 | 243-247 | 247-249 | 189-194 | 241-248 | 235-235 | 165-183 | 79-83 | 149-149 | 147-151 | 181-202 | 139-147 | 206-210 | 353-371 | 234-236 | 263-263 | 147-149 | 174-190 |
| O35-64 (DVIT1816) | -:- | 290-290 | 243-243 | 237-257 | 185-185 | 254-254 | 249-249 | 165-167 | 77-77 | 149-149 | 131-151 | 166-188 | 141-175 | 202-210 | 345-353 | 234-249 | 263-271 | 147-151 | 158-194 |
| *sylvestris* (DVIT3355.4) | -:- | -:- | 243-247 | 247-247 | 189-189 | -:- | 235-263 | 167-183 | 81-83 | 155-177 | 137-143 | 181-186 | 165-177 | 214-214 | 343-343 | 236-238 | 256-263 | 149-151 | 180-194 |
| *sylvestris* (DVIT3355.5) | -:- | -:- | 234-247 | 247-247 | 175-185 | 247-247 | 263-263 | 187-187 | 77-83 | 149-177 | 137-137 | 181-181 | 165-167 | 210-214 | 368-368 | 236-240 | 256-256 | 151-151 | 174-194 |
| *sylvestris* (DVIT3353.31) | -:- | -:- | 234-247 | 243-247 | 185-189 | 247-254 | 239-239 | 165-173 | 79-83 | 151-167 | 133-135 | 181-181 | 151-165 | 204-206 | 343-353 | 226-234 | 256-265 | 151-151 | 174-194 |
| *sylvestris* (DVIT3353.43) | -:- | 288-288 | 234-257 | 243-271 | 183-185 | 248-248 | 235-263 | 165-165 | 83-83 | 151-167 | 135-143 | 178-181 | 163-165 | 210-214 | 353-368 | 228-232 | 263-265 | 149-151 | 180-180 |
| *sylvestris* (DVIT3350.25) | -:- | 290-296 | 225-235 | 247-247 | 183-183 | 241-241 | 235-235 | 165-175 | 77-83 | 155-167 | 151-151 | 166-170 | 163-167 | 208-208 | 360-368 | 236-238 | 263-263 | 149-151 | 180-180 |
| *sylvestris* (DVIT3350.2) | -:- | 290-290 | 235-243 | 247-247 | 179-187 | 238-254 | 247-255 | 165-179 | 79-79 | 145-155 | 125-151 | 166-203 | 139-145 | 208-208 | 346-368 | 236-249 | 254-265 | 147-151 | 174-186 |

| | | | | | | | | | | | | | | | | | | | |
|---|---|---|---|---|---|---|---|---|---|---|---|---|---|---|---|---|---|---|---|
| *sylvestris* (DVIT3348.14) | -:- | -:- | 235-235 | 249-249 | -:- | 241-241 | 249-257 | 171-171 | -:- | 151-151 | -:- | 174-174 | 139-167 | -:- | 353-353 | 234-240 | 263-263 | -:- | -:- |
| *sylvestris* (DVIT3348.17) | -:- | -:- | 257-257 | 261-261 | 189-193 | 241-241 | 245-245 | 165-171 | 79-79 | 151-151 | 137-141 | 206-206 | 139-139 | 214-214 | 353-368 | 234-240 | 263-263 | 151-151 | 180-184 |
| *sylvestris* (DVIT3357.30) | -:- | -:- | 235-257 | 249-261 | 189-189 | -:- | 245-249 | 165-171 | 77-77 | 151-151 | 141-141 | 174-174 | 167-167 | 206-214 | 368-368 | 234-234 | 263-263 | 147-149 | 180-180 |
| *sylvestris* (DVIT3357.4)) | -:- | -:- | -:- | 261-261 | 189-193 | 246-246 | 245-245 | 165-171 | 77-79 | 151-151 | 137-141 | 174-174 | 139-167 | 214-214 | 353-353 | 234-240 | 263-263 | 149-151 | 180-180 |
| *sylvestris* (DVIT3351.23) | -:- | -:- | 233-253 | 257-261 | 181-181 | 241-247 | 239-249 | 165-165 | 77-79 | 151-151 | 137-145 | -:- | 165-175 | 210-214 | 363-368 | 226-236 | 256-263 | 149-151 | 186-186 |
| *sylvestris* (DVIT3351.27) | -:- | 288-294 | 253-275 | 249-261 | 179-181 | -:- | 239-249 | 169-183 | 79-83 | 149-151 | 143-147 | -:- | 163-163 | 210-214 | 363-368 | 228-236 | 263-263 | 149-151 | 186-186 |
| *sylvestris* (DVIT3349.12) | -:- | 294-294 | 225-271 | 247-271 | 179-185 | 247-254 | 239-249 | 175-175 | 79-79 | 155-163 | 123-141 | 172-182 | 163-163 | 204-206 | 360-360 | 240-240 | 263-263 | 151-153 | 174-194 |
| *sylvestris* (DVIT3349.8) | -:- | -:- | 247-271 | 247-247 | 183-183 | -:- | 239-251 | 165-165 | 79-81 | 159-163 | 133-137 | 202-205 | 163-163 | -:- | 360-368 | 240-245 | 263-265 | 149-153 | 182-184 |
| *sylvestris* (DVIT3356.38) | -:- | -:- | 247-247 | 247-255 | 189-189 | -:- | 239-239 | 165-197 | 77-77 | 159-177 | 135-137 | 178-181 | 151-163 | 204-210 | 343-368 | 228-236 | 256-263 | 151-151 | 172-182 |
| *sylvestris* (DVIT3356.33) | -:- | -:- | 234-247 | 243-243 | 189-189 | -:- | 239-239 | 167-167 | 77-83 | 159-167 | 133-135 | 181-202 | 163-163 | 204-214 | 353-368 | 236-236 | 256-263 | 151-151 | 182-194 |
| 545685 (PI# 545685) | -:- | 260-267 | 257-257 | -:- | 191-191 | -:- | -:- | -:- | -:- | 139-145 | 145-149 | 188-202 | 147-147 | -:- | -:- | -:- | -:- | -:- | -:- |
| 597257.03 (GVIT 859) | 297-297 | 310-324 | 247-255 | 239-245 | 185-189 | 243-247 | 239-241 | 167-167 | 77-77 | 139-159 | 133-141 | 148-192 | 161-165 | 216-221 | 328-347 | 246-246 | 261-263 | 147-169 | 172-176 |
| 588421.a (PI# 588421) | 323-326 | 294-302 | 227-237 | 222-249 | 185-196 | 248-262 | 253-253 | 173-179 | 77-81 | 141-155 | 131-159 | 166-214 | 143-157 | 204-214 | 312-312 | 246-258 | 263-271 | 147-149 | 170-184 |
| 588422.a (PI# 588422) | 311-315 | 288-300 | 227-245 | 230-237 | 183-185 | 247-254 | 229-247 | 165-165 | -:- | 147-167 | 135-141 | 164-172 | 153-165 | 204-210 | 312-330 | 236-254 | 261-263 | 147-161 | 162-186 |
| 588451.c (GVIT 813) | -:- | -:- | -:- | 237-237 | -:- | -:- | -:- | 167-167 | 79-79 | -:- | -:- | 182-182 | 171-171 | -:- | -:- | -:- | 267-267 | -:- | -:- |
| 588452.b (GVIT 814) | 309-309 | 302-304 | 245-245 | 222-239 | 183-187 | 247-251 | 243-243 | 167-187 | 81-81 | 169-169 | 139-143 | 180-180 | 153-157 | 206-208 | -:- | 234-248 | 261-261 | 147-153 | 174-178 |
| 588650.a (PI# 588650) | 313-325 | 294-296 | 217-235 | 235-235 | 181-181 | 254-254 | 247-261 | 165-169 | 77-81 | 155-159 | 139-149 | 166-186 | 141-165 | 204-210 | 368-384 | 236-236 | 261-261 | 147-149 | 174-186 |

| | | | | | | | | | | | | | | | | | | | |
|---|---|---|---|---|---|---|---|---|---|---|---|---|---|---|---|---|---|---|---|
| A-166-003 (PI# 597294) | **317-323** | **302-302** | **237-247** | **228-237** | **193-198** | **248-258** | **-:-** | **177-181** | **81-81** | **-:-** | **131-131** | **196-204** | **149-149** | **204-204** | **327-336** | **242-248** | **261-261** | **147-147** | **172-172** |
| *amurensis* (DVIT1158.4) | **301-317** | **298-304** | **243-243** | **243-243** | **183-197** | **252-256** | **241-245** | **167-167** | **81-81** | **153-155** | **129-129** | **166-184** | **167-171** | **204-214** | **329-329** | **234-236** | **-:-** | **147-147** | **170-172** |
| *amurensis* (DVIT1157.12) | **321-321** | **300-302** | **227-243** | **237-237** | **189-193** | **247-260** | **243-243** | **165-165** | **-:-** | **145-145** | **137-145** | **184-184** | **155-167** | **204-204** | **329-337** | **234-234** | **261-261** | **145-147** | **170-170** |
| *amurensis* (DVIT1158.1) | **321-321** | **300-302** | **-:-** | **243-243** | **183-197** | **247-252** | **239-241** | **167-167** | **81-87** | **149-161** | **129-129** | **166-184** | **167-171** | **204-214** | **329-335** | **232-236** | **261-261** | **147-147** | **170-172** |
| *amurensis* (DVIT2006.1) | **311-321** | **300-302** | **239-249** | **237-237** | **183-189** | **247-247** | **243-247** | **165-179** | **-:-** | **149-149** | **125-133** | **172-184** | **157-165** | **206-208** | **-:-** | **234-240** | **263-263** | **147-163** | **166-170** |
| *amurensis* (DVIT1156.2) | **315-321** | **302-316** | **237-249** | **241-245** | **187-191** | **246-251** | **241-245** | **167-167** | **87-87** | **145-159** | **131-131** | **162-172** | **157-165** | **204-204** | **329-329** | **226-234** | **261-261** | **147-147** | **168-172** |
| *amurensis* (DVIT1157.2) | **321-321** | **302-302** | **-:-** | **237-237** | **194-205** | **247-247** | **241-241** | **167-167** | **-:-** | **145-145** | **139-145** | **174-184** | **159-167** | **204-204** | **-:-** | **226-234** | **261-261** | **145-147** | **-:-** |
| *amurensis* (DVIT2005.5) | **323-323** | **300-302** | **229-229** | **233-237** | **183-194** | **251-273** | **243-243** | **165-167** | **83-83** | **147-153** | **129-137** | **188-196** | **155-163** | **204-204** | **329-329** | **232-234** | **261-261** | **145-147** | **172-174** |
| B-166-016 (597298.01) | **315-326** | **294-294** | **235-245** | **230-271** | **185-196** | **247-251** | **243-247** | **165-181** | **77-83** | **147-159** | **143-149** | **180-204** | **153-165** | **204-210** | **312-312** | **232-254** | **258-263** | **147-155** | **178-182** |
| B-166-019 (588715.01) | **315-327** | **294-294** | **235-245** | **230-271** | **185-195** | **247-247** | **243-247** | **171-181** | **81-83** | **147-167** | **143-149** | **176-204** | **165-167** | **210-210** | **340-385** | **232-234** | **263-263** | **147-155** | **182-186** |
| Beaumont (DVIT1432) | **323-329** | **284-308** | **233-239** | **236-236** | **187-215** | **225-225** | **247-249** | **171-185** | **79-79** | **147-151** | **135-135** | **202-202** | **143-143** | **204-204** | **348-360** | **252-252** | **254-254** | **149-161** | **198-198** |
| *betulifolia* (DVIT2596.1) | **297-299** | **-:-** | **221-241** | **239-241** | **183-183** | **-:-** | **239-239** | **167-167** | **77-77** | **155-155** | **131-137** | **154-174** | **151-155** | **-:-** | **323-323** | **246-246** | **261-269** | **159-159** | **168-174** |
| C-166-025 (597296.01) | **311-311** | **-:-** | **233-245** | **231-247** | **185-194** | **247-247** | **241-243** | **-:-** | **81-81** | **157-161** | **129-141** | **162-208** | **151-153** | **216-216** | **324-340** | **238-246** | **263-269** | **147-147** | **178-186** |
| C-166-026 (DVIT2550) | **311-311** | **-:-** | **233-243** | **231-247** | **185-189** | **-:-** | **241-243** | **165-165** | **81-81** | **161-171** | **129-141** | **162-208** | **151-151** | **216-216** | **324-324** | **238-238** | **261-269** | **147-147** | **170-178** |
| C-166-039 (597297.01) | **-:-** | **297-298** | **-:-** | **-:-** | **-:-** | **247-247** | **241-243** | **-:-** | **-:-** | **152-161** | **135-149** | **162-182** | **-:-** | **212-216** | **-:-** | **246-246** | **269-269** | **147-147** | **178-186** |
| C-166-043 (DVIT3192) | **301-311** | **296-297** | **245-245** | **231-247** | **185-189** | **247-247** | **243-245** | **165-165** | **-:-** | **151-151** | **129-135** | **162-168** | **151-153** | **212-216** | **324-324** | **238-246** | **261-263** | **147-147** | **178-178** |
| *coignetiae* (DVIT1159.10) | **317-319** | **300-302** | **233-247** | **237-253** | **183-187** | **247-251** | **243-243** | **167-181** | **89-89** | **147-159** | **133-133** | **186-188** | **147-157** | **212-214** | **345-345** | **234-240** | **267-269** | **146-153** | **166-184** |

| | | | | | | | | | | | | | | | | | | | |
|---|---|---|---|---|---|---|---|---|---|---|---|---|---|---|---|---|---|---|---|
| *coignetiae* (DVIT1159.3) | **317-319** | **282-300** | **233-255** | **237-253** | **181-183** | **247-251** | **243-243** | **179-189** | **89-89** | **149-151** | **133-133** | **180-180** | **147-175** | **204-206** | **341-346** | **234-234** | **261-269** | **146-146** | **178-184** |
| *coignetiae* (DVIT1159.9) | **317-321** | **302-304** | **237-245** | **237-243** | **189-199** | **247-252** | **239-241** | **167-169** | **81-87** | **149-155** | **129-129** | **164-184** | **167-171** | **204-214** | **329-329** | **234-234** | **261-261** | **146-146** | **170-170** |
| *ficifolia* (DVIT2008.5) | **293-326** | **304-312** | **229-241** | **241-241** | **203-203** | **247-247** | **241-241** | **169-181** | **85-85** | **157-157** | **141-143** | **164-208** | **151-167** | **204-204** | **322-322** | **244-246** | **263-267** | **145-147** | **166-166** |
| *ficifolia* (DVIT2008.7) | **293-293** | **308-312** | **227-229** | **241-241** | **194-197** | **247-247** | **241-241** | **169-179** | **85-85** | **153-157** | **141-143** | **182-184** | **167-167** | **204-218** | **322-322** | **244-244** | **263-267** | **147-147** | **166-166** |
| *ficifolia* (DVIT1160.7) | **293-293** | **304-308** | **227-227** | **241-241** | **191-194** | **246-246** | **243-243** | **179-181** | **85-85** | **153-153** | **135-143** | **182-182** | **167-167** | **204-204** | **322-322** | **244-244** | **265-267** | **149-151** | **166-186** |
| *flexuosa* (DVIT1385) | **307-327** | **290-300** | **237-249** | **243-243** | **183-209** | **-:-** | **255-261** | **167-171** | **79-79** | **145-151** | **133-141** | **174-196** | **143-153** | **204-208** | **361-361** | **244-250** | **-:-** | **147-155** | **174-194** |
| J-167-048 (597295.01) | **303-326** | **302-302** | **233-245** | **237-247** | **196-198** | **248-248** | **239-255** | **167-177** | **83-83** | **152-152** | **131-141** | **196-224** | **170-176** | **200-200** | **-:-** | **250-252** | **263-263** | **145-145** | **-:-** |
| *jacquemontii* (DVIT2349.13) | **289-319** | **294-294** | **235-235** | **245-245** | **185-187** | **241-254** | **241-249** | **169-175** | **79-79** | **155-155** | **131-135** | **164-164** | **141-165** | **204-214** | **347-353** | **236-236** | **267-267** | **149-149** | **170-172** |
| *jacquemontii* (DVIT2350.17) | **-:-** | **294-294** | **-:-** | **-:-** | **185-187** | **254-254** | **241-241** | **169-175** | **-:-** | **155-155** | **135-135** | **164-188** | **-:-** | **214-214** | **-:-** | **236-236** | **263-263** | **147-149** | **170-172** |
| *jacquemontii* (DVIT2354.7) | **289-319** | **294-294** | **235-235** | **245-251** | **185-187** | **241-241** | **241-249** | **169-175** | **79-79** | **149-155** | **131-135** | **164-188** | **141-141** | **204-214** | **347-347** | **236-248** | **263-267** | **147-149** | **170-172** |
| *jacquemontii* (DVIT2355.11) | **289-319** | **294-294** | **235-235** | **245-251** | **185-187** | **241-254** | **241-249** | **169-169** | **79-79** | **149-149** | **131-135** | **164-188** | **165-165** | **214-214** | **347-353** | **236-236** | **263-263** | **147-147** | **170-172** |
| Kali Dakh II (DVIT2539) | **289-309** | **290-290** | **233-233** | **251-255** | **185-189** | **241-247** | **249-249** | **165-165** | **77-77** | **155-173** | **137-151** | **158-202** | **165-165** | **204-214** | **347-361** | **226-250** | **267-273** | **149-151** | **172-172** |
| O35-59 (DVIT1815) | **289-289** | **290-290** | **229-229** | **247-247** | **185-187** | **254-254** | **251-255** | **169-175** | **-:-** | **149-149** | **131-141** | **162-166** | **141-157** | **200-204** | **344-344** | **248-250** | **263-271** | **147-147** | **166-166** |
| *piasezkii* (DVIT2027) | **297-297** | **324-238** | **247-255** | **239-245** | **183-185** | **243-247** | **239-241** | **167-167** | **81-81** | **139-159** | **131-133** | **148-192** | **161-165** | **206-228** | **-:-** | **246-248** | **261-269** | **147-147** | **172-176** |
| *piasezkii* (DVIT2032) | **295-299** | **312-318** | **231-241** | **241-241** | **183-185** | **245-247** | **225-239** | **165-171** | **77-81** | **139-151** | **151-153** | **180-182** | **165-167** | **216-220** | **322-328** | **244-246** | **261-271** | **147-171** | **168-168** |
| Thomas (DVIT1772) | **321-321** | **280-280** | **211-227** | **247-247** | **211-215** | **239-239** | **235-237** | **-:-** | **-:-** | **137-149** | **145-153** | **192-202** | **-:-** | **233-233** | **328-345** | **230-238** | **261-263** | **147-147** | **-:-** |
| Trayshed (DVIT1756) | **-:-** | **-:-** | **217-235** | **247-247** | **209-215** | **-:-** | **237-245** | **183-187** | **-:-** | **-:-** | **145-147** | **182-192** | **-:-** | **254-254** | **331-331** | **240-240** | **263-263** | **147-147** | **186-186** |

| | | | | | | | | | | | | | | | | | | | |
|---|---|---|---|---|---|---|---|---|---|---|---|---|---|---|---|---|---|---|---|
| Aurore (S. 5279) (DVIT3009) | **319-319** | **284-290** | **243-257** | **236-236** | **181-185** | **241-254** | **243-257** | **179-179** | **79-83** | **151-159** | **135-145** | **180-186** | **157-157** | **204-206** | **329-333** | **226-250** | **254-254** | **147-151** | **180-190** |
| Baco blanc (Baco 22A) (0000-0-289-04) | **307-321** | **294-296** | **243-257** | **249-271** | **181-187** | **238-247** | **235-239** | **169-179** | **79-83** | **159-161** | **125-133** | **172-172** | **161-163** | **204-206** | **346-360** | **226-226** | **263-263** | **147-149** | **174-184** |
| Chambourcin (JS 26.205) (C 67-00) | **315-319** | **284-290** | **251-257** | **239-249** | **185-189** | **-:-** | **239-251** | **171-171** | **79-83** | **147-159** | **133-145** | **164-186** | **147-161** | **206-214** | **333-360** | **238-244** | **254-254** | **145-147** | **174-196** |
| Couderc 13 (GVIT0342) | **-:-** | **290-290** | **261-281** | **239-255** | **187-187** | **-:-** | **237-239** | **-:-** | **83-83** | **-:-** | **143-155** | **182-192** | **167-167** | **204-208** | **336-360** | **226-260** | **-:-** | **147-149** | **180-186** |
| De Chaunac (Seibel 9549) (DVIT0257) | **319-319** | **284-290** | **241-247** | **255-255** | **181-187** | **247-262** | **251-261** | **165-171** | **79-83** | **147-159** | **133-133** | **164-214** | **157-167** | **202-206** | **347-357** | **252-262** | **254-265** | **145-151** | **174-180** |
| Jaeger 70 (DVIT0196) | **307-315** | **288-304** | **227-237** | **251-251** | **186-189** | **218-254** | **249-255** | **171-181** | **83-83** | **159-161** | **125-151** | **178-178** | **161-163** | **204-214** | **360-360** | **228-268** | **254-263** | **145-149** | **174-198** |
| Joannès Seyve 23416 (6039Mtp1) | **-:-** | **-:-** | **-:-** | **-:-** | **-:-** | **-:-** | **-:-** | **-:-** | **-:-** | **-:-** | **-:-** | **-:-** | **-:-** | **-:-** | **-:-** | **-:-** | **-:-** | **-:-** | **-:-** |
| JS23-416 (DVIT0197) | **319-319** | **290-290** | **227-257** | **255-271** | **185-189** | **241-241** | **235-261** | **165-179** | **77-79** | **147-159** | **133-139** | **164-180** | **147-147** | **204-206** | **-:-** | **226-234** | **254-265** | **145-155** | **178-184** |
| Kuhlmann 188-2 (DVIT0214) | **315-319** | **294-296** | **235-237** | **255-255** | **181-181** | **-:-** | **251-255** | **165-167** | **77-79** | **157-167** | **135-141** | **164-164** | **165-167** | **204-204** | **334-360** | **226-232** | **263-263** | **147-147** | **174-178** |
| Sev Lernatu PRG 2224 (DVIT2685) | **315-325** | **290-294** | **243-243** | **239-271** | **194-194** | **241-241** | **239-247** | **171-171** | **83-83** | **167-177** | **133-155** | **174-206** | **163-165** | **206-215** | **364-368** | **236-240** | **256-263** | **-:-** | **172-178** |
| SV 12.375 Villard blanc (HOP W11-13) | **-:-** | **290-306** | **233-235** | **239-255** | **181-189** | **226-254** | **237-251** | **171-183** | **77-79** | **147-159** | **133-143** | **174-182** | **147-147** | **206-210** | **333-368** | **232-236** | **254-265** | **147-149** | **174-178** |
| Vignoles (DVIT2741) | **319-327** | **290-298** | **237-259** | **249-249** | **185-185** | **241-247** | **239-251** | **165-171** | **79-79** | **147-159** | **125-149** | **182-186** | **161-165** | **206-214** | **329-329** | **226-246** | **254-254** | **149-155** | **174-192** |
