# Supplementary material for: Identification of mildew resistance in wild and cultivated Central Asian grape germplasm
Source: BMC Plant Biol. 2013 Oct 4;13:149. doi: 10.1186/1471-2229-13-149 (PMC3851849; doi:10.1186/1471-2229-13-149)
Supplement: Additional file 4: Table S4 — List of SSR markers and allele size ranges. [file 1471-2229-13-149-S4.pdf]

Supplementary Table S4. List of SSR markers and allele size ranges.

SSR marker	Chromosome	Dye	Alleles size range in base pairs
VVIp60	1	Hex	289 - 338
VVIb01	2	6-Fam	260 - 324
VVMD28	3	6-Fam	211 - 270
VVMD32	4	6-Fam	222 - 273
VMC4c6	5	Ned	142 - 174
VrZAG79	5	6-Fam	234 - 287
VVMD27	5	Ned	175 - 215
VMC2g2	6	6-Fam	115 - 149
VVMD21	6	Hex	218 - 273
VrZAG62	7	Hex	175 - 223
VVMD31	7	6-Fam	179 - 238
VVMD7	7	Ned	225 - 267
VMC1b11	8	Hex	156 - 197
VVIq52	9	6-Fam	75 - 89
VVIv37	10	6-Fam	137 - 181
VVMD25	11	Ned	234 - 275
VVS02	11	6-Fam	123 - 167
VMC4f3.1	12	Hex	148 - 224
VMC8g9	12	Hex	137 - 202
VVIh54	13	6-Fam	129 - 187
VMCNg4e10.1	13	Ned	200 - 291
sc47-18	13	Ned	203 - 249
SC8-0071-014	13	Ned	135 - 205
UDV124	13	6-Fam	174 - 278
VMC3d12	13	6-Fam	183 - 262
VVMD24	14	Ned	200 - 254
VVIv67	15	Hex	312 - 397
VVMD5	16	6-Fam	222 - 268
VVIn73	17	6-Fam	254 - 273
UDV108	18	Hex	200 - 282
VMC7f2	18	Hex	185 - 241
VVIn16	18	6-Fam	145 - 171
VMC2g6	18	6-Fam	125 - 141
VVIp31	19	6-Fam	158 - 204
