# Supplementary material for: Identification of mildew resistance in wild and cultivated Central Asian grape germplasm
Source: BMC Plant Biol. 2013 Oct 4;13:149. doi: 10.1186/1471-2229-13-149 (PMC3851849; doi:10.1186/1471-2229-13-149)
Supplement: Additional file 5: Table S5 — SSR allele data with four markers linked to the Ren1 locus. Alleles associated with powdery mildew resistance are in bold. Two previously identified and 10 newly identified powdery mildew resistant accessions are underlined. Missing data are indicated with a hyphen. [file 1471-2229-13-149-S5.pdf]

Supplementary Table S5. SSR allele data with four markers linked to the *Ren1* locus. Alleles associated with powdery mildew resistance are in bold. Two previously identified and 10 newly identified powdery mildew resistant accessions are underlined. Missing data are indicated with a hyphen.

Variety name and #id	VMCNg4e10.1	sc47-18	SC08-0071-014	UDV124
588421.a (PI# 588421)	239-271	219-221	**143**-147	226-234
588650.a (588650)	266-291	237-237	161-161	200-**216**
A Kalatchel (2076Mtp1)	236-257	231-**249**	159-173	208-218
A'asemi S1 (1979-0-2219-S1)	251-286	229-240	157-169	**216**-218
Ag Kiourdach p.e. (2842Mtp1)	254-263	241-**249**	159-161	188-194
Agaday (TYR VI 10-05)	254-**260**	**249**-**249**	-:-	188-214
Ahmeh Sal apyrène (2982Mtp1)	251-277	236-240	159-199	**216**-220
Alia boka (2858Mtp1)	251-**260**	216-**249**	169-173	198-212
Alloued Zeine (DVIT0337)	251-257	240-**249**	157-169	212-218
amurensis (DVIT1157.12)	**260**-271	222-226	141-164	176-190
amurensis (DVIT2006.1)	**260**-**260**	216-225	147-163	190-191
amurensis (DVIT1156.2)	266-269	221-**249**	141-164	189-192
amurensis (DVIT1157.2)	**260**-271	-:-	141-164	176-190
Aragatzi (2113Mtp1)	**260**-277	236-**249**	169-199	220-220
Arybata (DVIT2442.6)	251-251	242-242	169-169	208-**216**
Asgari 01 (2001-9-8093-01)	236-251	231-240	157-159	**216**-218
Askari (DVIT0343)	257-289	231-231	159-169	**216**-230
B-166-016 (597298.01)	271-289	231-233	161-169	200-**216**
Baharat Early (DVIT2306)	239-289	222-231	169-169	**216**-230
Baidh Ul Haman (DVIT0358)	236-**260**	242-**249**	159-169	212-**216**
Bargoon (DVIT2499)	254-**260**	**249**-**249**	161-169	188-212
Bayad1 (749Mtp1)	-:-	227-231	159-165	**216**-218
Berurargoon (DVIT2501)	251-254	242-**249**	161-165	**216**-220
Black Kishmish (DVIT2055)	**260**-277	236-240	157-199	218-220
Bouaki nor (2507Mtp1)	239-**260**	241-**249**	159-169	198-212
Boyalsing I (DVIT2496)	251-257	216-**249**	159-173	218-230
C-166-039 (597297.01)	**260**-263	-:-	166-178	194-194
C-166-043 (DVIT3192)	263-263	217-217	**143**-177	192-194
Chaani noir (2676Mtp1)	251-254	240-**249**	159-161	188-**216**

Chamo (DVIT2730)	254-254	242-**249**	161-161	190-190
<u>Chirai obak (1186Mtp1)</u>	<u>254-**260**</u>	<u>227-**249**</u>	<u>**143**-175</u>	<u>**216**-228</u>
Cirmisi Sap De Sudak (DVIT0313)	251-277	236-240	159-199	190-**216**
Dais-el-anz (DVIT0569)	236-**260**	231-**249**	159-169	212-218
DK #05 (Turkmn 13362)	251-257	216-**249**	169-173	212-212
DK Melkii Chernyi (Turkmn 13375)	251-257	216-**249**	169-173	198-220
Dschan Im Isium (DVIT0315)	239-251	240-240	159-159	194-**216**
Fayoumi (DVIT2636)	236-251	-:-	159-159	**216**-218
Gaschochi (DVIT2537)	248-254	222-**249**	159-173	218-230
Gechi Kyrlen (Turkmn 3028)	236-271	215-231	159-159	**216**-218
Guzal Kara (TYR VI 13-09)	251-**260**	241-**249**	162-169	212-212
Henab (DVIT0417)	236-251	231-240	157-173	198-**216**
Hisakasy (DVIT0759)	236-248	231-**249**	159-161	188-218
Hosargoon (DVIT2503)	254-**260**	242-**249**	169-169	190-212
Huseine Rozvoj (TYR VI 13-15)	-:-	241-**249**	159-169	198-212
<u>Husseine (DVIT0576)</u>	<u>251-**260**</u>	<u>242-**249**</u>	<u>**143**-167</u>	<u>**216**-220</u>
Irki (1752Mtp1)	251-254	-:-	157-165	**216-216**
JS23-416 (DVIT0197)	227-236	220-231	159-163	**216**-218
Kabuli (DVIT2497)	251-254	207-**249**	159-165	208-218
Kali Dakh II (DVIT2539)	248-254	227-**249**	159-161	188-218
Kara Dzhidzhigi (DVIT2322)	251-**260**	216-240	157-173	198-218
Kara Kaytak (DVIT2445.12)	251-257	**249-249**	169-169	230-232
Kara Palvan (2780Mtp1)	239-271	215-**249**	159-169	198-218
<u>Karadzhandal (DVIT2323)</u>	<u>254-**260**</u>	<u>227-**249**</u>	<u>**143**-175</u>	<u>**216**-220</u>
Kashiri (DVIT2451)	248-254	216-**249**	159-173	198-218
Katta Kurgan (DVIT0774)	251-**260**	242-**249**	165-169	212-222
<u>Khalchili (DVIT0431)</u>	<u>**260**-271</u>	<u>215-**249**</u>	<u>**143**-159</u>	<u>212-218</u>
Khatmi (2190Mtp1)	254-271	215-**249**	159-161	188-198
Khawngi (DVIT2919)	227-254	203-**249**	**143**-161	228-232
Khorestini (DVIT**260**5)	236-**260**	231-**249**	159-169	212-218

Kibraïski (2781Mtp1)	**260**-266	232-240	157-159	190-218
Kichmich rond (1678Mtp5)	257-277	236-**249**	169-199	198-220
Kishmish Sorkh (DVIT0437)	236-271	215-231	159-159	**216**-218
Kisil izium (0Mtp589)	236-**260**	231-**249**	159-169	218-230
<u>Kismish Vatkana (20008-14 B)</u>	<u>236-**260**</u>	<u>231-**249**</u>	<u>**143**-159</u>	<u>**216**-218</u>
Kouldjinski (DVIT2680)	236-239	231-**249**	159-169	198-218
Koz ouzioum (2635Mtp1)	236-254	231-**249**	159-161	-:-
Kule Dary (DVIT1070)	236-254	231-239	159-175	**216**-218
Kush Dzhumurtka (Turkmn 18820)	236-257	231-**249**	159-169	194-218
Lal Sorkh (DVIT0442)	**260**-277	236-**249**	169-199	212-220
<u>Late Vavilov (ARM Q01-16)</u>	<u>236-**260**</u>	<u>231-**249**</u>	<u>**143**-159</u>	<u>**216**-218</u>
Mamidon (Turkmn 3025)	257-271	215-**249**	159-169	212-218
Mamidon Deli (Turkmn 6984)	257-271	215-**249**	159-169	212-218
<u>Matrassa (2642Mtp2)</u>	<u>248-251</u>	<u>216-240</u>	<u>**143**-173</u>	<u>198-**216**</u>
Mehdi 01 (2001-9-8101-01)	251-271	-:-	159-159	**216**-218
Mellei (Turkmn 3030)	236-271	215-231	159-159	**216**-218
Monaca (1742Mtp1)	236-254	231-**249**	159-161	194-218
Mourvedre Famellestadt (DVIT0319)	254-257	215-**249**	161-173	**216**-220
Narma (2648Mtp2)	-:-	231-**249**	159-161	-:-
Neeli (DVIT2514)	**260**-271	237-**249**	163-169	192-218
Nimrang (TYR VI 15-17)	236-239	231-**249**	159-169	200-220
Noir D'automne (DVIT0330)	251-254	216-**249**	161-173	212-214
<u>O34-16 (DVIT1803)</u>	<u>**260**-271</u>	<u>215-**249**</u>	<u>**143**-159</u>	<u>**216**-218</u>
Pakistan Collection 25168 (DVIT2282)	254-**260**	216-**249**	161-169	188-212
Pakistan Collection 25180 (DVIT2269)	254-**260**	-:-	-:-	188-212
Pakistan Collection 25237 (DVIT2272)	257-**260**	**249**-**249**	169-169	212-214
Pakistan Collection 25241 (DVIT2271)	242-**260**	217-229	169-173	212-232
Pakistan Collection 25296	254-257	216-**249**	159-169	214-218

(DVIT2283)				
Pakistan Collection 25311 (DVIT2264)	251-257	**249**-**249**	159-169	212-218
Parargoon (DVIT2502)	251-**260**	216-**249**	169-173	212-218
Persian R27 (DVIT2755)	248-254	**249**-**249**	161-161	202-228
Pervenetz Praskoveisky (2651Mtp2)	245-263	221-**249**	162-169	208-212
Précoce d'Astrakan (0Mtp928)	251-254	216-**249**	-:-	198-198
Rajoulan (0Mtp950)	257-268	240-**249**	-:-	212-220
Razakiia piembiana (2737Mtp1)	251-283	215-240	159-159	214-**216**
Red Ohanez (DVIT0499)	251-257	232-**249**	159-169	212-**216**
Rhazaki (Pa 1882) (TYR VI 16-09)	251-257	232-240	159-159	194-**216**
Rhazaki Anatolico (DVIT0608)	236-254	231-231	159-159	**216**-218
Rhazaki De Crete (DVIT0501)	251-257	232-240	159-159	194-**216**
Rhazaki Mavro (DVIT0555)	266-283	215-232	159-159	**216**-218
Rish BabaHOP L04-19)	254-**260**	227-**249**	169-175	212-220
Rizamat (DVIT2338)	251-**260**	216-**249**	169-173	198-212
Sabza angur (0Mtp1007)	239-**260**	227-231	157-159	218-220
Sary Kiriak (0Mtp1031)	257-257	**249**-**249**	169-169	198-212
Shahani 01 (2001-9-7097-01)	251-271	215-240	159-159	**216**-218
<u>Sochal (DVIT1126)</u>	<u>239-**260**</u>	<u>216-**249**</u>	<u>**143**-173</u>	<u>198-**216**</u>
<u>Soïaki (2657Mtp1)</u>	<u>251-**260**</u>	<u>216-**249**</u>	<u>**143**-173</u>	<u>**216**-218</u>
Soultani (0Mtp1071)	236-**260**	231-**249**	159-169	218-218
Sourkhak biely (2856Mtp1)	**260**-263	240-**249**	157-169	-:-
sylvestris (DVIT3353.43)	251-271	216-242	145-165	190-**216**
sylvestris (DVIT3350.25)	251-254	216-**249**	161-173	198-228
sylvestris (DVIT3350.2)	251-251	216-219	193-193	**216**-**216**
sylvestris (DVIT3351.23)	**260**-277	216-236	**143**-199	198-198
<u>sylvestris (DVIT3351.27)</u>	<u>**260**-263</u>	<u>216-242</u>	<u>**143**-145</u>	<u>194-208</u>
sylvestris (DVIT3349.12)	263-263	203-216	**143**-163	218-231
Tagobi (2659Mtp1)	239-271	231-**249**	159-159	198-218
Taifi (DVIT2174)	233-239	231-**249**	159-169	198-218

Taka Sago (TYR VI 17-03)	242-283	215-**249**	159-159	204-214
Tarnau (TYR VI 17-07)	239-257	**249-249**	169-169	198-198
Tchatyrbac (664Mtp1)	251-251	240-240	159-162	208-**216**
Thomas (DVIT1772)	**260**-274	216-221	-:-	208-262
Tolstokory (1985Mtp2)	254-263	242-**249**	161-173	206-206
Trayshed (DVIT1756)	**260-260**	215-215	-:-	224-278
Tufachi S1 (1985-0-2415-S1)	257-263	241-**249**	159-169	194-212
Tuia-tiche (2760Mtp1)	251-257	242-**249**	167-169	198-220
Uzbekistanian Muscat (DVIT2072)	**260**-289	231-**249**	169-169	212-230
Varuschkin (0Mtp1165)	251-254	216-**249**	161-173	188-198
Vassarga tchernaia (2510Mtp1)	251-**260**	231-**249**	**143**-159	198-**216**
Volgo Don (TYR VI 17-17)	254-**260**	232-**249**	159-169	212-218
Zerk (DVIT0606)	251-271	-:-	159-173	**216**-218
