# Supplementary material for: Identification of mildew resistance in wild and cultivated Central Asian grape germplasm
Source: BMC Plant Biol. 2013 Oct 4;13:149. doi: 10.1186/1471-2229-13-149 (PMC3851849; doi:10.1186/1471-2229-13-149)
Supplement: Additional file 6: Table S6 — List of accessions screened for powdery mildew resistance in a no-spray field nursery trial. Bold and italicized accessions were included as known resistant and susceptible controls. Powdery mildew symptoms were recorded on leaves and canes. Plants in group A were screened in 2009; plants in group B were screened in year 2010; plants in group C were screened in 2012. Group D accessions are maintained in the Vassal collection (INRA, France) – they were evaluated under unsprayed greenhouse evaluations. [file 1471-2229-13-149-S6.pdf]

Supplementary Table S6. List of accessions screened for powdery mildew resistance in a no-spray field nursery trial. Bold accessions were included as known resistant and susceptible controls. Newly identified powdery mildew resistant accessions are underlined. Powdery mildew symptoms were recorded on leaves and canes. Plants in group A were screened in 2009; plants in group B were screened in year 2010; plants in group C were screened in 2012. Group D accessions are maintained in the Vassal collection (INRA, France) – they were evaluated under unsprayed greenhouse evaluations.

			Leaf					Cane				
	**Accession name**	**No. of replicates**	**Min**	**Max**	**Mean**	**Std Dev**	**Std Err**	**Min**	**Max**	**Mean**	**Std Dev**	**Std Err**
**Group A**	05389-01	7	0	1	0.17	0.41	0.17	0	2	1.00	1.10	0.45
	05389-02	6	0	3	1.83	1.17	0.48	0	2	1.00	0.63	0.26
	05391-01	8	0	0	0.00	0.00	0.00	0	2	0.38	0.74	0.26
	06715-105	7	0	1	0.29	0.49	0.18	0	1	0.43	0.53	0.20
	06715-159	9	0	4	1.67	1.50	0.50	0	2	0.89	0.78	0.26
	07358-14	11	1	5	3.09	1.30	0.39	1	5	2.73	1.27	0.38
	07754-10	18	3	5	3.67	0.77	0.18	4	5	4.94	0.24	0.06
	588422.a	8	3	5	4.50	0.76	0.27	3	5	4.63	0.74	0.26
	588452.b	4	0	0	0.00	0.00	0.00	0	3	0.75	1.50	0.75
	**Agaday**	10	4	5	4.80	0.42	0.13	3	5	4.60	0.70	0.22
	**Asgari 01**	10	3	5	4.40	0.70	0.22	3	5	4.20	0.79	0.25
	**Askari**	8	3	5	3.63	0.74	0.26	2	5	3.25	0.89	0.31
	**Aubun**	12	1	4	2.75	0.97	0.28	0	5	2.58	1.38	0.40
	B-166-019	8	0	5	3.00	1.51	0.53	0	5	3.00	1.51	0.53
	**Baharat Early**	6	3	5	4.33	0.82	0.33	4	5	4.33	0.52	0.21

Bargoon	10	2	5	3.70	0.82	0.26	1	5	3.20	1.40	0.44
Barrett 9	11	0	0	0.00	0.00	0.00	0	0	0.00	0.00	0.00
Blanc Du Bois	11	0	1	0.27	0.47	0.14	0	1	0.55	0.52	0.16
Beaumont	8	0	1	0.50	0.53	0.19	0	3	1.00	1.07	0.38
Black Kishmish	10	4	5	4.50	0.53	0.17	4	5	4.20	0.42	0.13
**Carignane**	14	3	5	4.57	0.65	0.17	2	5	4.50	0.94	0.25
Cirmisi Sap De Sudak	6	2	4	3.50	0.84	0.34	1	5	3.00	1.41	0.58
Dais-el-anz	8	3	5	4.13	0.64	0.23	2	5	3.88	0.99	0.35
**DC1-39**	12	0	1	0.08	0.29	0.08	0	0	0.00	0.00	0.00
Dschan Im Isium	10	2	5	3.70	0.95	0.30	1	5	3.60	1.35	0.43
Durif 01	10	0	4	2.70	1.34	0.42	1	4	2.70	0.82	0.26
Fayoumi	10	1	4	3.30	1.06	0.33	2	4	3.50	0.71	0.22
Guzal Kara	10	4	5	4.60	0.52	0.16	2	5	3.70	1.16	0.37
Henab	4	4	5	4.75	0.50	0.25	4	5	4.25	0.50	0.25
Hosargoon	10	2	5	4.00	1.05	0.33	3	5	3.90	0.74	0.23
<u>Husseine</u>	*<u>9</u>*	*<u>0</u>*	*<u>2</u>*	*<u>1.11</u>*	*<u>0.93</u>*	*<u>0.31</u>*	*<u>0</u>*	*<u>3</u>*	*<u>1.33</u>*	*<u>1.00</u>*	*<u>0.33</u>*
J-167-048	6	0	3	0.67	1.21	0.49	0	3	0.67	1.21	0.49
Jane De Smirna	10	0	4	3.20	1.23	0.39	0	3	2.00	1.15	0.37
JS23.416	10	0	1	0.10	0.32	0.10	0	1	0.20	0.42	0.13
Kara Dzhidzhigi	9	4	5	4.89	0.33	0.11	0	5	4.00	1.66	0.55
**Karadzhandal**	12	0	1	0.67	0.49	0.14	0	1	0.42	0.51	0.15
Katta Kurgan	6	4	5	4.67	0.52	0.21	4	5	4.83	0.41	0.17

Khalchili	*10*	*0*	*4*	*1.40*	*1.26*	*0.40*	*0*	*4*	*2.00*	*1.33*	*0.42*
Khorestini	10	2	5	3.80	1.03	0.33	3	5	3.80	0.92	0.29
Khusaine Red	10	2	5	4.30	0.95	0.30	3	5	4.10	0.88	0.28
Kule Dary	8	3	5	4.25	0.71	0.25	1	4	2.38	1.30	0.46
Lal	10	3	5	4.30	0.67	0.21	3	5	3.90	0.57	0.18
Lal Sorkh	6	4	5	4.50	0.55	0.22	4	5	4.17	0.41	0.17
Late Vavilov	*12*	*0*	*3*	*1.08*	*0.79*	*0.23*	*1*	*4*	*2.42*	*0.79*	*0.23*
Mehdi 01	8	2	5	4.50	1.07	0.38	4	5	4.88	0.35	0.13
Neeli	6	2	5	3.67	1.03	0.42	3	4	3.67	0.52	0.21
Noir D'automne	10	2	5	3.80	1.03	0.33	0	5	3.20	1.99	0.63
Parargoon	8	4	5	4.50	0.53	0.19	4	5	4.88	0.35	0.13
Pakistan Collection 25168	8	2	5	4.13	1.13	0.40	3	5	4.25	0.71	0.25
Pakistan Collection 25180	10	3	5	4.00	0.94	0.30	2	5	3.90	0.99	0.31
Pakistan Collection 25227	12	3	5	4.00	0.74	0.21	3	5	3.92	0.67	0.19
Pakistan Collection 25234	12	3	5	4.42	0.67	0.19	1	5	4.25	1.22	0.35
Pakistan Collection 25241	10	2	5	4.40	0.97	0.31	3	5	4.10	0.74	0.23
Pakistan Collection 25296	8	1	5	3.00	1.31	0.46	3	5	4.00	0.76	0.27
Red Ohanez	8	4	5	4.38	0.52	0.18	3	5	4.38	0.74	0.26
Rhazaki Anat.	10	2	5	4.10	1.10	0.35	0	5	2.80	1.69	0.53
Rish Baba	10	3	5	4.30	0.67	0.21	3	5	4.30	0.67	0.21
Rizamat	8	4	5	4.63	0.52	0.18	4	5	4.75	0.46	0.16
Ruby Seedless	7	4	5	4.57	0.53	0.20	2	5	3.71	1.11	0.42

	Shahani 01	10	4	5	4.80	0.42	0.13	2	5	4.40	0.97	0.31
	Sochal	12	1	3	2.42	0.67	0.19	0	3	0.83	0.94	0.27
	Vignoles	10	0	1	0.20	0.42	0.13	0	1	0.20	0.42	0.13
	**Villard blanc**	11	0	2	0.45	0.82	0.25	0	4	2.14	1.27	0.38
	Volgo Don	11	3	5	4.36	0.67	0.20	3	5	4.45	0.82	0.25
	Zerk	10	5	5	5.00	0.00	0.00	4	5	4.80	0.42	0.13
**Group B**	545685	10	0	0	0.00	0.00	0.00	0	0	0.00	0.00	0.00
	588421.a	10	4	5	4.70	0.48	0.15	0	5	1.40	1.78	0.56
	588650.a	10	4	5	4.50	0.53	0.17	0	5	2.20	2.35	0.74
	A-166-003	10	3	5	4.50	0.85	0.27	0	5	2.10	2.28	0.72
	**Agaday**	10	5	5	5.00	0.00	0.00	0	5	2.40	2.17	0.69
	**Asgari 01**	10	4	5	4.90	0.32	0.10	0	5	2.20	2.25	0.71
	**Askari**	10	4	5	4.30	0.48	0.15	0	5	2.70	2.36	0.75
	**Aubun**	6	3	5	4.17	0.75	0.31	0	4	1.83	2.04	0.83
	B-166-016	10	4	5	4.80	0.42	0.13	0	5	2.00	2.26	0.71
	**Baharat Early**	10	3	5	4.10	0.74	0.23	0	4	1.90	1.85	0.59
	Baidh Ul Haman	10	5	5	5.00	0.00	0.00	0	5	2.10	2.23	0.71
	Black Kishmis (DVIT0358)	10	4	5	4.90	0.32	0.10	0	4	1.90	2.02	0.64
	C-166-043	10	0	1	0.10	0.32	0.10	0	2	0.55	0.83	0.26
	**Carignane**	10	3	5	4.70	0.67	0.21	0	5	3.40	1.58	0.50
	**DC1-39**	10	0	1	0.20	0.42	0.13	0	0	0.00	0.00	0.00
	DVIT1158.1 (*V. amurensis*)	10	0	5	3.00	1.63	0.52	0	5	1.70	2.06	0.65

DVIT1158.4 (*V. amurensis*)	2	3	5	4.00	1.41	1.00	0	3	1.50	2.12	1.50
DVIT1159.10 (*V. amurensis*)	10	2	5	4.40	0.97	0.31	0	4	1.00	1.63	0.52
DVIT2006.1 (*V. amurensis*)	3	0	5	3.00	2.65	1.53	0	3	1.00	1.73	1.00
DVIT1159.3 (*V. coignetiae*)	4	1	5	3.25	1.71	0.85	0	3	0.75	1.50	0.75
DVIT1160.7 (*V. ficifolia*)	10	0	5	3.40	1.35	0.43	0	4	1.10	1.79	0.57
DVIT2008.5 (*V. ficifolia*)	10	3	5	4.30	0.67	0.21	0	5	1.10	1.85	0.59
DVIT2008.7 (*V. ficifolia*)	10	3	5	4.30	0.67	0.21	0	5	1.30	1.95	0.62
V. flexuosa	10	0	4	2.90	1.20	0.38	0	4	1.50	1.65	0.52
V. piasezkii (DVIT2032)	10	0	3	1.40	1.26	0.40	0	1	0.10	0.32	0.10
DVIT2350.17 (*V. jacquemontii*)	10	4	5	4.50	0.53	0.17	0	5	2.20	2.15	0.68
DVIT2354.7 (*V. jacquemontii*)	10	4	5	4.70	0.48	0.15	0	4	1.60	1.78	0.56
DVIT2355.11 (*V. jacquemontii*)	10	4	5	4.80	0.42	0.13	0	5	2.20	1.99	0.63
Grignolino	10	4	5	4.70	0.48	0.15	0	5	1.70	1.83	0.58
Himrisnky	8	4	5	4.75	0.46	0.16	0	5	2.38	2.56	0.91
**Karadzhandal**	10	0	3	0.50	0.97	0.31	0	3	0.75	1.03	0.33
Kishmish Sorkh	6	5	5	5.00	0.00	0.00	0	5	2.50	2.74	1.12
Magnolia (*M. rotundifolia*)	4	0	1	0.25	0.50	0.25	0	0	0.00	0.00	0.00
Mataro	10	3	5	4.40	0.70	0.22	0	5	2.40	1.96	0.62
Mourv Famel	10	4	5	4.50	0.53	0.17	0	5	2.50	2.27	0.72
Rhazaki (Pa 1882)	10	1	5	4.10	1.29	0.41	0	5	2.80	2.49	0.79
Rhazaki De Crete	10	4	5	4.90	0.32	0.10	0	5	2.60	2.37	0.75
Rhazaki Mavro	10	4	5	4.80	0.42	0.13	0	5	2.50	2.32	0.73

	Thomas (*M. rotundifolia*)	4	0	0	0.00	0.00	0.00	0	0	0.00	0.00	0.00
	Trayshed (*M. rotundifolia*)	10	0	0	0.00	0.00	0.00	0	0	0.00	0.00	0.00
	Ugni blanc	10	4	5	4.70	0.48	0.15	0	5	3.30	1.95	0.62
	Uzbek Muscat	10	3	5	4.00	0.67	0.21	0	5	2.50	2.27	0.72
	**Villard blanc**	10	0	2	0.80	0.79	0.25	0	4	1.40	1.58	0.50
**Group C**	DVIT3349.12	20	3	5	4.30	0.67	0.21	0	5	2.70	2.26	0.72
	DVIT3351.23	20	4	5	4.40	0.52	0.16	0	5	3	1.89	0.60
	DVIT3351.27	*20*	*0*	*4*	*0.40*	*1.26*	*0.40*	*0*	*2*	*0.70*	*0.82*	*0.26*
	**Karadzhandal**	4	0	0	0.00	0.00	0.00	1	2	1.50	0.71	0.50
	O34-16	*20*	*0*	*2*	*0.70*	*0.95*	*0.30*	*0*	*3*	*1.10*	*1.20*	*0.38*
	Ruby Seedless	4	4	5	4.50	0.71	0.50	4	5	4.50	0.71	0.50
	**Carignane**	4	4	5	4.50	0.71	0.50	5	5	5.00	0.00	0.00
**Group D**	**Disease evaluations for samples from Vassal collection**											
	Kandari noir (1746Mtp I)					1						
	Kara djandjal (2777Mtp1)					0						
	Chirai obak (1186Mtp1)					0 - 1						
	Vassarga tchernaia (2510Mtp1)					0 - 1						
