# Supplementary material for: Identification of mildew resistance in wild and cultivated Central Asian grape germplasm
Source: BMC Plant Biol. 2013 Oct 4;13:149. doi: 10.1186/1471-2229-13-149 (PMC3851849; doi:10.1186/1471-2229-13-149)
Supplement: Additional file 7: Table S7 — Observed probability of identity [P(Id)], probability of exclusion of a single parent and parent pair, and cumulated power of exclusion of a single parent and parent pair calculated from 394 unique accessions using FAMOZ on 34 SSR markers. [file 1471-2229-13-149-S7.pdf]

Supplementary Table S7. Observed probability of identity [P(Id)], probability of exclusion of a single parent and parent pair, and cumulated power of exclusion of a single parent and parent pair calculated from 394 unique accessions using FAMOZ on 34 SSR markers.

SSR marker	Number of identical pairs of genotypes	Observed probability of identity	Single parent observed exclusion (%)	Single parent cumulated exclusions (%)	Parent pair observed exclusion (%)	Parent pair cumulated exclusions (%)
VVIv67	1463	0.188977	72.6	72.6	95.8	95.8
VMC8g9	47	6.07 E -4	69.8	91.7	94.9	99.8
VMC7f2	3	3.90 E -5	69.1	97.4	94.6	100.0
VVMD27	2	2.60 E -5	68.1	99.2	94.3	100.0
UDV124	2	2.60 E -5	65.4	99.7	93.1	100.0
VrZAG62	2	2.60 E -5	65.3	99.9	93.6	100.0
VrZAG79	2	2.60 E -5	64.8	100.0	93.2	100.0
VVMD21	1	1.30 E -5	63.1	100.0	92.1	100.0
VVIn16	1	1.30 E -5	62.9	100.0	92.2	100.0
sc47-18	0	0.000000	59.3	100.0	90.3	100.0
VMC1b11	0	0.000000	58.4	100.0	90.0	100.0
VMCNg4e10.1	0	0.000000	56.0	100.0	88.6	100.0
VVMD5	0	0.000000	55.7	100.0	88.6	100.0
VMC2g6	0	0.000000	55.5	100.0	88.6	100.0
VVIn73	0	0.000000	55.4	100.0	88.7	100.0
VVMD7	0	0.000000	53.4	100.0	87.2	100.0
VVIh54	0	0.000000	50.8	100.0	87.3	100.0
SC08-0071-014	0	0.000000	50.4	100.0	84.8	100.0
VVS02	0	0.000000	48.3	100.0	84.1	100.0
VVIq52	0	0.000000	47.9	100.0	82.9	100.0
VVIv37	0	0.000000	47.6	100.0	84.1	100.0
VVMD32	0	0.000000	47.0	100.0	84.4	100.0

VVMD28	0	0.000000	43.7	100.0	80.4	100.0
VVMD31	0	0.000000	41.1	100.0	78.3	100.0
VVMD25	0	0.000000	39.6	100.0	76.5	100.0
VVIb01	0	0.000000	38.7	100.0	76.3	100.0
VMC4f3.1	0	0.000000	38.7	100.0	76.4	100.0
VMC3d12	0	0.000000	36.9	100.0	74.1	100.0
VMC2g2	0	0.000000	32.5	100.0	69.4	100.0
VVIp60	0	0.000000	30.1	100.0	66.6	100.0
UDV108	0	0.000000	29.5	100.0	65.9	100.0
VVIp31	0	0.000000	28.6	100.0	62.7	100.0
VVMD24	0	0.000000	20.1	100.0	55.9	100.0
VMC4c6	0	0.000000	10.6	100.0	35.3	100.0
