# Supplementary material for: Identification of mildew resistance in wild and cultivated Central Asian grape germplasm
Source: BMC Plant Biol. 2013 Oct 4;13:149. doi: 10.1186/1471-2229-13-149 (PMC3851849; doi:10.1186/1471-2229-13-149)
Supplement: Additional file 8: Table S8 — SSR marker profiles for 13 accessions with 34 markers. Eight additional markers were added to six accessions to verify parent progeny relationships. Missing data are noted with hyphens. [file 1471-2229-13-149-S8.pdf]

Supplementary Table S8. SSR marker profiles for 13 accessions with 34 markers. Eight additional markers were added to six accessions to verify parent progeny relationships. Missing data are noted with hyphens.

Marker name	Thompson seedless	Kismish Vatkana (20008-14 B)	Vassarga tchernaia (2510Mtp1)	Sochal (DVIT1126)	Karadzhandal (DVIT2323)	Late Vavilov (ARM Q01-16)	Khalchili (DVIT0431)	Yarghouti (TYR VI 17-19)	Chirai obak (1186Mtp1)	Husseine (DVIT0576)	O34-16 (DVIT1803)	Matrassa (2642Mtp2)	Soiaki (2657Mtp1)
**VVIp60**	315-317	315-319	315-319	315-317	315-319	315-319	315-319	315-317	315-317	313-315	-:-	315-315	319-319
**VVIb01**	290-298	294-298	290-294	294-298	290-294	290-290	298-298	290-298	294-294	290-298	290-294	288-290	290-290
**VVMD28**	217-243	217-233	233-243	243-245	233-257	233-243	243-245	217-243	235-243	243-245	247-277	239-270	243-243
**VVMD32**	249-249	249-271	249-271	257-271	249-271	249-271	249-271	249-271	249-255	257-271	249-249	243-251	243-257
**VMC4c6**	152-158	152-164	164-164	160-164	162-164	160-164	158-158	158-164	152-152	158-164	152-152	153-164	164-168
**VrZAG79**	247-259	247-259	247-255	247-251	247-251	247-249	247-247	247-251	247-257	247-249	249-251	239-259	249-251
**VVMD27**	181-194	179-194	179-194	179-185	179-194	179-194	181-194	181-185	181-194	179-194	179-193	181-185	181-185
**VMC2g2**	129-129	129-129	129-135	123-129	129-137	129-131	129-129	123-129	-:-	129-137	127-129	135-137	131-137
**VVMD21**	247-254	247-254	247-247	247-247	247-247	247-247	247-247	247-254	241-246	241-249	-:-	241-241	241-247
**VrZAG62**	189-189	189-203	201-203	189-203	189-197	189-197	189-189	189-189	189-205	189-197	205-205	195-203	189-205
**VVMD31**	212-212	212-212	212-212	196-212	196-210	210-212	210-212	210-212	212-214	196-212	216-220	212-212	212-212
**VVMD7**	239-253	239-249	249-249	243-249	243-249	249-253	243-253	243-253	243-243	233-243	247-255	249-262	233-243
**VMC1b11**	165-183	165-183	165-169	169-193	165-169	165-169	165-183	173-183	165-169	165-193	165-185	183-183	165-165
**VVIq52**	79-83	77-83	77-77	77-79	77-79	77-79	77-79	79-83	79-83	77-79	81-81	77-79	83-83

**VVIv37**	155-177	177-177	155-177	167-177	177-177	177-177	155-173	155-177	145-177	173-177	149-159	159-167	167-177
**VVMD25**	238-248	238-238	238-240	240-244	240-244	240-248	238-254	238-262	254-256	244-254	240-240	238-240	254-256
**VVS02**	145-151	137-145	125-137	125-151	125-155	125-151	143-145	143-145	137-143	141-155	133-133	135-145	135-141
**VMC4f3.1**	188-190	164-190	164-190	164-202	182-202	182-202	172-186	186-190	172-182	172-172	178-178	186-202	172-182
**VMC8g9**	164-171	164-171	159-164	164-171	164-171	170-171	173-176	171-173	164-170	164-176	164-176	164-176	159-161
**sc47-18**	231-236	231-249	231-249	216-249	227-249	231-249	215-249	215-236	227-249	242-249	215-249	216-240	216-249
**SC08-0071-014**	159-159	143-159	143-159	143-173	143-175	143-159	143-159	159-199	143-175	143-167	143-159	143-173	143-173
**UDV124**	218-220	216-218	198-216	198-216	216-220	216-218	212-218	218-220	216-228	216-220	216-218	198-216	216-218
**VMC3d12**	201-234	198-234	195-198	195-198	195-198	198-248	202-217	200-202	198-206	195-198	199-202	195-200	198-202
**VMCNg4e10.1**	236-277	236-260	251-260	239-260	254-260	236-260	260-271	271-277	254-260	251-260	260-271	248-251	251-260
**VVIh54**	163-165	139-163	139-165	139-165	139-139	139-165	165-177	165-165	151-165	139-139	139-175	151-165	139-151
**VVMD24**	206-215	206-215	206-214	-:-	210-214	210-215	206-218	206-215	206-206	215-215	204-208	206-210	206-214
**VVIv67**	353-353	353-353	353-360	353-353	353-360	360-376	353-360	353-360	353-366	353-353	353-353	357-368	347-353
**VVMD5**	234-234	234-240	234-240	234-238	234-240	234-240	238-240	234-240	236-240	234-240	228-246	228-240	228-240
**VVIn73**	261-263	261-263	256-263	256-263	263-267	263-263	263-267	261-267	256-263	263-263	263-267	256-265	256-263
**UDV108**	204-218	218-218	218-218	218-242	242-244	244-244	218-242	218-242	244-244	242-242	242-242	236-236	242-242
**VMC2g6**	129-135	129-135	129-135	129-129	129-135	129-135	129-135	129-135	129-129	129-129	129-135	129-129	131-131
**VMC7f2**	197-199	197-199	199-199	199-199	197-199	197-203	197-199	197-199	199-203	199-199	197-197	199-199	-:-
**VVIn16**	151-155	-:-	151-151	151-155	155-155	151-155	149-155	151-155	151-151	149-149	147-151	151-151	149-151

**VVIp31**	178-182	178-182	182-182	178-182	182-186	182-186	190-194	178-194	158-188	178-186	174-178	178-182	188-194
**VMC2c10.1**	163-173	147-173	147-153	147-163	147-173	159-173	-:-	-:-	-:-	-:-	-:-	-:-	-:-
**VMC2e7**	169-173	169-173	161-173	159-161	159-173	159-173	-:-	-:-	-:-	-:-	-:-	-:-	-:-
**VMC4d4**	159-162	159-162	162-162	162-162	162-162	159-162	-:-	-:-	-:-	-:-	-:-	-:-	-:-
**VMC7g5**	164-164	164-164	164-170	164-170	164-164	164-164	-:-	-:-	-:-	-:-	-:-	-:-	-:-
**VMC2h10**	128-132	128-132	112-128	118-128	116-128	116-128	-:-	-:-	-:-	-:-	-:-	-:-	-:-
**VMC5c1**	145-151	151-151	145-151	145-145	145-145	145-145	-:-	-:-	-:-	-:-	-:-	-:-	-:-
**VMC3e12**	153-153	153-153	153-153	127-153	127-153	153-153	-:-	-:-	-:-	-:-	-:-	-:-	-:-
**VMC3b7.2**	93-105	93-105	93-93	93-111	107-121	107-121	-:-	-:-	-:-	-:-	-:-	-:-	-:-

Formatted Table
