# Supplementary material for: Identification of mildew resistance in wild and cultivated Central Asian grape germplasm
Source: BMC Plant Biol. 2013 Oct 4;13:149. doi: 10.1186/1471-2229-13-149 (PMC3851849; doi:10.1186/1471-2229-13-149)
Supplement: Additional file 9: Table S9 — The estimated coefficients of membership proportions values (Q-values) for the three ancestral genetic clusters inferred with STRUCTURE. [file 1471-2229-13-149-S9.pdf]

Supplementary Table S9. The estimated coefficients of membership proportions values (Q-values) for the three ancestral genetic clusters inferred with STRUCTURE.

Accession ID	Species	Accession name	Source country /Collection	Group A (Species)	Group B (O34-16)	Group C (TSL)
DVIT1159.9	*V. coignetiae*		Unknown	0.998	0.001	0.001
DVIT2008.5	*V. ficifolia*		South Korea	0.998	0.001	0.001
GVIT 859	*V. piasezkii*	597257.03	China	0.997	0.002	0.001
DVIT1158.1	*V. amurensis*		China	0.997	0.002	0.001
DVIT2008.7	*V. ficifolia*		South Korea	0.997	0.001	0.001
DVIT2027	*V. piasezkii*		Asia	0.997	0.002	0.002
DVIT1158.4	*V. amurensis*		China	0.996	0.002	0.002
597296.01	*V. romanetii*	C-166-025	China	0.996	0.002	0.002
597294.01	*Vitis* species	A-166-003	China	0.995	0.003	0.002
DVIT1157.12	*V. amurensis*		China	0.995	0.002	0.003
DVIT1157.2	*V. amurensis*		China	0.995	0.003	0.002
DVIT2596.1	*V. betulifolia*		China	0.995	0.003	0.003
DVIT2005.5	*V. amurensis*		China	0.994	0.003	0.003
597295.01	*Vitis* species	J-167-048	China	0.994	0.003	0.003
DVIT2550	*V. romanetii*	C-166-026	China	0.993	0.004	0.003
DVIT1159.3	*V. coignetiae*		Unknown	0.992	0.004	0.005
DVIT1156.2	*V. amurensis*		China	0.99	0.006	0.004
DVIT3192	*V. romanetii*	C-166-043	China	0.99	0.007	0.003

DVIT2032	*V. piasezkii*		Asia	0.99	0.004	0.006
DVIT1160.7	*V. ficifolia*		China	0.987	0.007	0.006
DVIT1159.10	*V. coignetiae*		Unknown	0.985	0.006	0.009
GVIT 814	*V. amurensis*	588452.b	USSR	0.982	0.012	0.006
DVIT2006.1	*V. amurensis*		South Korea	0.958	0.02	0.022
DVIT1385	*V. flexousa*		Unknown	0.919	0.068	0.014
DVIT1815	*V. lanata*	O35-59	Afghanistan	0.896	0.072	0.033
PI# 588421	*V. yenshanensis*	588421.a	China	0.77	0.037	0.193
PI# 588422	*V. yenshanensis*	588422.a	China	0.759	0.19	0.051
597298.01	*Vitis* species	B-166-016	China	0.565	0.382	0.053
DVIT0434	*V. vinifera*	Khir Ghulaman	Afghanistan	0.401	0.293	0.307
DVIT3356.38	*V. sylvestris*		Armenia	0.002	0.996	0.003
DVIT1805	*V. sylvestris*	O34-46	Iran	0.001	0.996	0.003
DVIT3353.31	*V. sylvestris*		Armenia	0.001	0.996	0.002
DVIT3348.17	*V. sylvestris*		Georgia	0.001	0.996	0.002
DVIT3357.4	*V. sylvestris*		Georgia	0.001	0.996	0.002
DVIT3353.43	*V. sylvestris*		Armenia	0.002	0.995	0.003
DVIT3357.30	*V. sylvestris*		Georgia	0.002	0.995	0.002
2509Mtp1	*V. vinifera*	Boulany	Russia	0.002	0.995	0.004
DVIT1806	*V. sylvestris*	O34-54	Iran	0.001	0.995	0.004
1183Mtp1	*V. vinifera*	Alü tierskü	Russia	0.001	0.995	0.004
DVIT0773	*V. vinifera*	Kara Lakana	USSR	0.001	0.995	0.004

2642Mtp2	*V. vinifera*	Matrassa	Azerbaijan	0.001	0.995	0.004
DVIT0673	*V. vinifera*	Buaki	Russia	0.003	0.994	0.003
DVIT3355.5	*V. sylvestris*		Armenia	0.002	0.994	0.004
1985Mtp2	*V. vinifera*	Tolstokory	Russia	0.001	0.994	0.005
DVIT1809	*V. sylvestris*	O35-11	Iran	0.003	0.993	0.004
DVIT1807	*V. sylvestris*	O34-55	Iran	0.002	0.993	0.005
DVIT0307	*V. vinifera*	Al Borla	USSR	0.002	0.993	0.005
DVIT0329	*V. vinifera*	Kovalewka	USSR	0.001	0.993	0.006
DVIT3355.4	*V. sylvestris*		Armenia	0.004	0.992	0.004
DVIT0312	*V. vinifera*	Blanc De Crimei	USSR	0.002	0.992	0.006
0Mtp780	*V. vinifera*	Nassau	Russia	0.002	0.992	0.006
TYR VI 10-03	*V. vinifera*	Afuz-Ali Urmasti	Unknown	0.001	0.992	0.007
857Mtp1	*V. vinifera*	De Hengril	Russia	0.001	0.992	0.007
DVIT1808	*V. sylvestris*	O35-07	Iran	0.002	0.991	0.007
2737Mtp1	*V. vinifera*	Razakiia piembiana	Russia	0.001	0.991	0.008
DVIT3356.33	*V. sylvestris*		Armenia	0.005	0.99	0.005
DVIT2442.6	*V. sylvestris*	Arybata	Turkmenistan	0.003	0.99	0.007
TYR VI 13-17	*V. vinifera*	Jane De Smirna	Unknown	0.002	0.99	0.008
1737Mtp1	*V. vinifera*	Schiradzouli violet	Iran	0.002	0.99	0.008
0Mtp1073	*V. vinifera*	Starinky	Russia	0.001	0.99	0.009
0Mtp1165	*V. vinifera*	Varuschkin	Russia	0.001	0.99	0.008
DVIT3351.27	*V. sylvestris*		Armenia	0.002	0.989	0.008

Turkmn 21551	*V. vinifera*	Karga Dili	Turkmenistan	0.002	0.989	0.009
TYR VI 16-09	*V. vinifera*	Rhazaki (Pa 1882)	Unknown	0.002	0.989	0.009
0Mtp318	*V. vinifera*	Doppelaugen	Azerbaijan	0.001	0.989	0.01
0Mtp565	*V. vinifera*	Kanfet isium	Russia	0.001	0.989	0.009
DVIT2641	*V. vinifera*	Leanoy	USSR	0.001	0.989	0.01
1218Mtp1	*V. vinifera*	Tavkveri	Azerbaijan	0.001	0.989	0.01
TYR VI 15-13	*V. vinifera*	Mzivani	Unknown	0.009	0.988	0.003
DVIT3349.12	*V. sylvestris*		Georgia	0.008	0.988	0.004
DVIT0320	*V. vinifera*	Murma Isium	USSR	0.002	0.988	0.009
DVIT0310	*V. vinifera*	Barmak Isium	Turkey	0.001	0.988	0.011
DVIT3350.25	*V. sylvestris*		Georgia	0.009	0.987	0.004
DVIT0311	*V. vinifera*	Bias Kukuzeti	USSR	0.001	0.986	0.013
DVIT2306	*V. vinifera*	Baharat Early	India	0.008	0.984	0.008
DVIT2603	*V. vinifera*	Shingargoon	Pakistan	0.002	0.984	0.014
DVIT0305	*V. vinifera*	Abla Aganin Isium	USSR	0.001	0.984	0.015
0Mtp225	*V. vinifera*	Chassany	Azerbaijan	0.001	0.984	0.014
DVIT2293	*V. vinifera*	Pakistan Collection 25265	Pakistan	0.005	0.983	0.012
2678Mtp2	*V. vinifera*	Chirvan chakki	Azerbaijan	0.001	0.983	0.016
Turkmn 13381	*V. vinifera*	DK #17	Turkmenistan	0.002	0.982	0.016
DVIT0413	*V. vinifera*	Gros Colman	USSR	0.001	0.982	0.017
DVIT2451	*V. vinifera*	Kashiri	Pakistan	0.001	0.982	0.016
DVIT1841	*V. vinifera*	Beli Potok	Yugoslavia	0.012	0.981	0.007

DVIT0343	*V. vinifera*	Askari	Afghanistan	0.004	0.981	0.015
DVIT2496	*V. vinifera*	Boyalsing I	Pakistan	0.003	0.981	0.016
2951Mtp1	*V. vinifera*	Kaytagi	Russia	0.001	0.981	0.018
2190Mtp1	*V. vinifera*	Khatmi	Russia	0.001	0.981	0.017
2842Mtp1	*V. vinifera*	Ag Kiourdach p.e.	Azerbaijan	0.001	0.98	0.019
DVIT0314	*V. vinifera*	Demir Kara	USSR	0.001	0.98	0.019
DVIT1813	*V. sylvestris*	O35-50	Afghanistan	0.002	0.979	0.02
DVIT0328	*V. vinifera*	Himrisnky	USSR	0.005	0.978	0.017
DVIT2440.9	*V. sylvestris*	Ayedere	Turkmenistan	0.002	0.978	0.02
Turkmn 13374	*V. vinifera*	DK Melkii Krasnyi	Turkmenistan	0.001	0.978	0.021
DVIT0313	*V. vinifera*	Cirmisi Sap De Sudak	USSR	0.002	0.977	0.022
2666Mtp1	*V. vinifera*	Zimliansky noir	Russia	0.001	0.977	0.022
Turkmn 13359	*V. vinifera*	DK #02	Turkmenistan	0.001	0.976	0.022
DVIT2539	*V. jacquemontii*	Kali Dakh II	Pakistan	0.016	0.974	0.01
DVIT2447.9	*V. sylvestris*	Uzuntakoy	Turkmenistan	0.002	0.974	0.025
Turkmn 13361	*V. vinifera*	DK #04	Turkmenistan	0.001	0.974	0.025
DVIT2501	*V. vinifera*	Berurargoon	Pakistan	0.002	0.972	0.026
DVIT2510	*V. vinifera*	Fatai	Pakistan	0.002	0.972	0.026
Turkmn 13379	*V. vinifera*	DK #21 Chernyi	Turkmenistan	0.002	0.97	0.027
Turkmn 6272	*V. vinifera*	DK #9	Turkmenistan	0.001	0.97	0.028
DVIT0499	*V. vinifera*	Red Ohanez	Russia	0.001	0.97	0.029
2648Mtp2	*V. vinifera*	Narma	Russia	0.002	0.969	0.029

DVIT2730	*V. vinifera*	Chamo	Pakistan	0.002	0.968	0.031
DVIT2532	*V. vinifera*	Churgoon	Pakistan	0.002	0.968	0.03
DVIT2688	*V. vinifera*	Tchilar	Russia	0.002	0.968	0.03
DVIT3349.8	*V. sylvestris*	sylvestris	Georgia	0.029	0.966	0.005
DVIT0783	*V. vinifera*	Koptcha	Russia	0.008	0.965	0.027
DVIT0501	*V. vinifera*	Rhazaki De Crete	Greece	0.002	0.965	0.033
DVIT2498	*V. vinifera*	Millishun	Pakistan	0.002	0.964	0.033
DVIT0319	*V. vinifera*	Mourvedre Famellestadt	USSR	0.001	0.962	0.037
Turkmn 16392	*V. vinifera*	Chol Uzyum	Turkmenistan	0.003	0.959	0.038
DVIT0759	*V. vinifera*	Hisakasy	Russia	0.001	0.959	0.04
Turkmn 13365	*V. vinifera*	DK #08	Turkmenistan	0.006	0.958	0.035
DVIT2664	*V. vinifera*	Zimsko Belo	Yugoslavia	0.001	0.958	0.041
DVIT0597	*V. vinifera*	Salomani	Iraq	0.001	0.956	0.043
DVIT2537	*V. vinifera*	Gaschochi	Pakistan	0.003	0.955	0.041
DVIT1042	*V. vinifera*	Mermark	Iraq	0.003	0.955	0.042
Turkmn 13358	*V. vinifera*	DK #01	Turkmenistan	0.002	0.953	0.044
2271Mtp2	*V. vinifera*	Baïan chirei	Azerbaijan	0.001	0.953	0.046
1983-0-2424-S1	*V. vinifera*	Voivoginiova S1	USSR	0.001	0.953	0.046
DVIT2445.12	*V. sylvestris*	Kara Kaytak	Turkmenistan	0.007	0.951	0.043
0Mtp1795	*V. vinifera*	Mesisti rose	Russia	0.002	0.951	0.047
0000-0-4432-S1	*V. vinifera*	Fetyaska S1	Russia	0.002	0.95	0.048
DVIT2447.4	*V. sylvestris*	Uzuntakoy	Turkmenistan	0.006	0.949	0.045

DVIT2495	*V. vinifera*	Kini Yatch	Pakistan	0.01	0.946	0.044
DVIT0315	*V. vinifera*	Dschan Im Isium	USSR	0.001	0.945	0.053
TYR VI 11-13	*V. vinifera*	Caus X Pearl Csaba	Yugoslavia	0.005	0.942	0.053
2651Mtp2	*V. vinifera*	Pervenetz Praskoveisky	Russia	0.003	0.942	0.055
DVIT2685	*V. vinifera*	Sev Lernatu PRG 2224	Russia	0.001	0.942	0.056
664Mtp1	*V. vinifera*	Tchatyrbac	Uzbekistan	0.001	0.942	0.057
DVIT2531	*V. vinifera*	Torgoon	Pakistan	0.002	0.941	0.057
DVIT0634	*V. vinifera*	Alburla	USSR	0.003	0.94	0.056
DVIT0338	*V. vinifera*	Alulu	Iraq	0.002	0.937	0.061
DVIT0308	*V. vinifera*	Asma	USSR	0.001	0.936	0.063
0Mtp1031	*V. vinifera*	Sary Kiriak	Azerbaijan	0.001	0.936	0.063
654Mtp1	*V. vinifera*	Tuia-tiche p.e.	Russia	0.002	0.934	0.065
0Mtp928	*V. vinifera*	Précoce d'Astrakan	Iran	0.002	0.933	0.064
2635Mtp1	*V. vinifera*	Koz ouzioum	Russia	0.001	0.93	0.069
DVIT2919	*V. vinifera*	Khawngi	India	0.059	0.927	0.014
DVIT2440.1	*V. sylvestris*	Ayedere	Turkmenistan	0.006	0.927	0.067
DVIT2442.1	*V. sylvestris*	Arybata	Turkmenistan	0.002	0.927	0.071
DVIT0330	*V. vinifera*	Noir D'automne	USSR	0.001	0.926	0.072
DVIT3351.23	*V. sylvestris*		Armenia	0.002	0.921	0.077
DVIT1802	*V. sylvestris*	O33-60	Iran	0.015	0.91	0.074
DVIT1811	*V. sylvestris*	O35-41	Iran	0.001	0.91	0.089
2597Mtp1	*V. vinifera*	Peikani	Iran	0.001	0.909	0.09

TYR VI 17-11	*V. vinifera*	Uzbekistan Muscat	Unknown	0.011	0.908	0.081
DVIT1798	*V. sylvestris*	O30-44	Iran	0.008	0.908	0.084
2505Mtp1	*V. vinifera*	Assylkara	Russia	0.002	0.907	0.09
0Mtp750	*V. vinifera*	Mouchketny	Russia	0.02	0.905	0.074
DVIT1812	*V. sylvestris*	O35-47	Iran	0.003	0.904	0.093
Turkmn 13375	*V. vinifera*	DK Melkii Chernyi	Turkmenistan	0.012	0.902	0.086
DVIT1800	*V. sylvestris*	O30-53	Afghanistan	0.024	0.901	0.076
DVIT1152	*V. vinifera*	Selection 2798 (seedless)	Yugoslavia	0.002	0.901	0.098
DVIT2263	*V. vinifera*	Pakistan Collection 25275	Pakistan	0.097	0.896	0.007
DVIT1804	*V. sylvestris*	O34-29	Iran	0.009	0.895	0.096
DVIT0555	*V. vinifera*	Rhazaki Mavro	Unknown	0.001	0.891	0.108
0000-0-2411-S1	*V. vinifera*	Trapanlarin kara S1	USSR	0.004	0.874	0.122
DVIT2636	*V. vinifera*	Fayoumi	Egypt	0.032	0.866	0.102
DVIT1799	*V. sylvestris*	O30-51	Afghanistan	0.012	0.866	0.123
DVIT2755	*V. vinifera*	Persian R27	Unknown	0.121	0.865	0.014
TYR VI 10-05	*V. vinifera*	Agaday	Unknown	0.001	0.859	0.139
2088Mtp1	*V. vinifera*	Askari noir	Iran	0.002	0.847	0.151
DVIT1803	*V. sylvestris*	O34-16	Iran	0.031	0.845	0.123
2640Mtp1	*V. vinifera*	Lkeni noir	Azerbaijan	0.001	0.841	0.158
DVIT2446.12	*V. sylvestris*	Yuvankala	Turkmenistan	0.002	0.839	0.159
1679Mtp2	*V. vinifera*	Kechmisch aly violet	Iran	0.003	0.837	0.16
1981-0-2233-S1	*V. vinifera*	Bez el Anza S1	Egypt	0.002	0.83	0.168

Turkmn 13377	*V. vinifera*	DK #39	Turkmenistan	0.001	0.825	0.174
DVIT2536	*V. vinifera*	Dardari	Pakistan	0.002	0.824	0.174
DVIT2283	*V. vinifera*	Pakistan Collection 25296	Pakistan	0.002	0.819	0.179
DVIT2507	*V. vinifera*	Neelilay	Pakistan	0.001	0.816	0.182
2676Mtp1	*V. vinifera*	Chaani noir	Azerbaijan	0.001	0.811	0.188
DVIT2349.13	*V. jacquemontii*		Pakistan	0.254	0.736	0.011
DVIT2168	*V. vinifera*	Shtur Angur	USSR	0.002	0.733	0.265
TYR VI 17-03	*V. vinifera*	Taka Sago	Japan	0.173	0.72	0.107
DVIT2355.11	*V. jacquemontii*		Pakistan	0.265	0.714	0.021
DVIT2497	*V. vinifera*	Kabuli	Pakistan	0.005	0.705	0.29
DVIT2296	*V. vinifera*	Pakistan Collection 25290	Pakistan	0.014	0.691	0.295
Turkmn 30748	*V. vinifera*	Orion	Turkmenistan	0.001	0.687	0.311
DVIT3350.2	*V. sylvestris*		Georgia	0.307	0.684	0.009
DVIT2503	*V. vinifera*	Hosargoon	Pakistan	0.002	0.658	0.34
DVIT2444.19	*V. sylvestris*	Kochtemyr	Turkmenistan	0.013	0.65	0.337
2687Mtp1	*V. vinifera*	Yaï izium rose	Russia	0.002	0.578	0.42
DVIT2354.7	*V. jacquemontii*		Pakistan	0.419	0.572	0.009
0Mtp950	*V. vinifera*	Rajoulan	Russia	0.002	0.52	0.478
588650	*V. yenshanensis*	588650.a	China	0.477	0.517	0.006
1750Mtp1	*V. vinifera*	Raziki	Yemen	0.003	0.511	0.485
DVIT2446.9	*V. sylvestris*	Yuvankala	Turkmenistan	0.004	0.492	0.504
588715.01	*Vitis species*	B-166-019	China	0.45	0.463	0.087

2113Mtp1	*V. vinifera*	Aragatzi	Armenia	0.001	0.002	0.997
0Mtp429	*V. vinifera*	Gora Chirine faux	Iran	0.001	0.002	0.997
DVIT0428	*V. vinifera*	Kandahar	Unknown	0.001	0.002	0.997
DVIT2323	*V. vinifera*	Karadzhandal	USSR	0.001	0.002	0.997
DVIT0774	*V. vinifera*	Katta Kurgan	USSR	0.001	0.002	0.997
DVIT2605	*V. vinifera*	Khorestini	Pakistan	0.001	0.002	0.997
ARM Q01-16	*V. vinifera*	Late Vavilov	Turkmenistan	0.001	0.002	0.997
DVIT2338	*V. vinifera*	Rizamat	Uzbekistan	0.001	0.002	0.997
TYR VI 16-21	*V. vinifera*	Shakar Angur	USSR	0.001	0.002	0.997
0Mtp1071	*V. vinifera*	Soultani	Uzbekistan	0.001	0.002	0.997
	V. vinifera	Thompson Seedless		0.001	0.002	0.997
DVIT0462	*V. vinifera*	Monukka	Afghanistan	0.001	0.001	0.997
DVIT2322	*V. vinifera*	Kara Dzhidzhigi	Uzbekistan	0.001	0.004	0.996
2858Mtp1	*V. vinifera*	Alia boka	Uzbekistan	0.001	0.003	0.996
2001-9-8093-01	*V. vinifera*	Asgari 01	Iran	0.001	0.003	0.996
DVIT2084	*V. vinifera*	Khalili	Afghanistan	0.001	0.003	0.996
TYR VI 14-19	*V. vinifera*	Kishmishi	Unknown	0.001	0.003	0.996
Turkmn 19697	*V. vinifera*	Kismish Turkmenskii	Turkmenistan	0.001	0.003	0.996
DVIT2680	*V. vinifera*	Kouldjinski	Russia	0.001	0.003	0.996
DVIT0442	*V. vinifera*	Lal Sorkh	Afghanistan	0.001	0.003	0.996
2783Mtp1	*V. vinifera*	Rouchaki	USSR	0.001	0.003	0.996
0Mtp587	*V. vinifera*	Sahebi	Afghanistan	0.001	0.003	0.996

DVIT0510	*V. vinifera*	Sahibi Sorkh	Afghanistan	0.001	0.003	0.996
388Mtp2	*V. vinifera*	Sateni tcherny	Armenia	0.001	0.003	0.996
2671Mtp1	*V. vinifera*	Tchiliaki belyi	Tajikistan	0.001	0.003	0.996
2760Mtp1	*V. vinifera*	Tuia-tiche	Uzbekistan	0.001	0.003	0.996
TYR VI 17-17	*V. vinifera*	Volgo Don	Uzbekistan	0.001	0.003	0.996
DVIT2928	*V. vinifera*	Tana-Kuzi	Unknown	0.002	0.002	0.996
2677Mtp2	*V. vinifera*	Chakar angour de Tachkent	Uzbekistan	0.001	0.002	0.996
DVIT0402	*V. vinifera*	Fahri	Afghanistan	0.001	0.002	0.996
HOP L04-19	*V. vinifera*	Rish Baba	Unknown	0.001	0.002	0.996
0Mtp1597	*V. vinifera*	Sultanine noire faux	Uzbekistan	0.001	0.002	0.996
DVIT2055	*V. vinifera*	Black Kishmish	Russia	0.002	0.004	0.995
Turkmn 6218	*V. vinifera*	Ekdona Turkmenskaya	Turkmenistan	0.001	0.004	0.995
2780Mtp1	*V. vinifera*	Kara Palvan	Uzbekistan	0.001	0.004	0.995
DVIT0432	*V. vinifera*	Khaldar	Afghanistan	0.001	0.004	0.995
DVIT0435	*V. vinifera*	Kishmishi	Afghanistan	0.001	0.004	0.995
0Mtp589	*V. vinifera*	Kisil izium	Russia	0.001	0.004	0.995
Turkmn 3025	*V. vinifera*	Mamidon	Turkmenistan	0.001	0.004	0.995
TYR VI 15-17	*V. vinifera*	Nimrang	Russia	0.001	0.004	0.995
0Mtp1007	*V. vinifera*	Sabza angur	Tajikistan	0.001	0.004	0.995
DVIT0509	*V. vinifera*	Sahibi	Afghanistan	0.001	0.004	0.995
0Mtp1010	*V. vinifera*	Saïd guliami	Uzbekistan	0.001	0.004	0.995
2079Mtp1	*V. vinifera*	Shirazi	Iran	0.001	0.004	0.995

DVIT2174	*V. vinifera*	Taifi	USSR	0.001	0.004	0.995
DVIT2071	*V. vinifera*	Kishmish Of Vir	USSR	0.002	0.003	0.995
2507Mtp1	*V. vinifera*	Bouaki nor	Uzbekistan	0.001	0.003	0.995
DVIT0576	*V. vinifera*	Husseine	Afghanistan	0.001	0.003	0.995
Turkmn 29892	*V. vinifera*	Keshmesh Heshrau	Turkmenistan	0.001	0.003	0.995
DVIT0431	*V. vinifera*	Khalchili	Afghanistan	0.001	0.003	0.995
TYR VI 17-19	*V. vinifera*	Yarghouti	Unknown	0.001	0.003	0.995
2856Mtp1	*V. vinifera*	Sourkhak biely	Uzbekistan	0.002	0.005	0.994
2083Mtp1	*V. vinifera*	Askudi	Iran	0.001	0.005	0.994
2857Mtp1	*V. vinifera*	Chtour angour	Uzbekistan	0.001	0.005	0.994
Turkmn 13388	*V. vinifera*	DK #10	Turkmenistan	0.001	0.005	0.994
DVIT2500	*V. vinifera*	Gungargoon	Pakistan	0.001	0.005	0.994
TYR VI 13-15	*V. vinifera*	Huseine Rozvoj	Unknown	0.001	0.005	0.994
20008-14 B	*V. vinifera*	Kismish Vatkana	Uzbekistan	0.001	0.005	0.994
0Mtp1475	*V. vinifera*	Liali bidona	Azerbaijan	0.001	0.005	0.994
DVIT1126	*V. vinifera*	Sochal	USSR	0.001	0.005	0.994
1748Mtp1	*V. vinifera*	Abjouch	Afghanistan	0.002	0.004	0.994
DVIT0437	*V. vinifera*	Kishmish Sorkh	Afghanistan	0.002	0.004	0.994
2788Mtp1	*V. vinifera*	Pinger putao	China	0.002	0.004	0.994
Turkmn 3028	*V. vinifera*	Gechi Kyrlen	Turkmenistan	0.001	0.004	0.994
1742Mtp1	*V. vinifera*	Monaca	Afghanistan	0.001	0.004	0.994
DVIT0335	*V. vinifera*	Kalamak	Afghanistan	0.003	0.003	0.994

Turkmn 13364	*V. vinifera*	DK #07	Turkmenistan	0.001	0.006	0.993
Turkmn 6982	*V. vinifera*	Kara Terbash	Turkmenistan	0.001	0.006	0.993
Turkmn 6984	*V. vinifera*	Mamidon Deli	Turkmenistan	0.001	0.006	0.993
2952Mtp1	*V. vinifera*	Otcha bala	Uzbekistan	0.001	0.006	0.993
0Mtp703	*V. vinifera*	Matrassa	Russia	0.002	0.005	0.993
DVIT0416	*V. vinifera*	Hassaine	Unknown	0.002	0.004	0.993
Turkmn 19652	*V. vinifera*	Ali Shaitan	Turkmenistan	0.001	0.007	0.992
0Mtp428	*V. vinifera*	Golodan	Afghanistan	0.001	0.007	0.992
2663Mtp2	*V. vinifera*	Khalili tcherni	Iran	0.001	0.007	0.992
2736Mtp1	*V. vinifera*	Nimrang rouge	Uzbekistan	0.001	0.007	0.992
Turkmn 6304	*V. vinifera*	Sumbarskii Bekyi	Turkmenistan	0.001	0.007	0.992
Turkmn 3026	*V. vinifera*	Irtyk Yaprak	Turkmenistan	0.002	0.006	0.992
2691Mtp1	*V. vinifera*	Parkent	Uzbekistan	0.001	0.006	0.992
DVIT2917	*V. vinifera*	Kali Sag	Unknown	0.004	0.004	0.992
2690Mtp1	*V. vinifera*	Kara Kaltak	Uzbekistan	0.001	0.008	0.991
Turkmn 18820	*V. vinifera*	Kush Dzhumurtka	Turkmenistan	0.001	0.008	0.991
0Mtp640	*V. vinifera*	Liali Yakdona	Kazakhstan	0.001	0.008	0.991
DVIT2040	*V. vinifera*	Ab Jusht	Afghanistan	0.002	0.007	0.991
2649Mtp1	*V. vinifera*	Noulizok	Uzbekistan	0.001	0.007	0.991
0Mtp27	*V. vinifera*	Anab-e-Shabi	India	0.001	0.009	0.99
Turkmn 6977	*V. vinifera*	DK Belyi	Turkmenistan	0.001	0.009	0.99
TYR VI 17-07	*V. vinifera*	Tarnau	USSR	0.001	0.009	0.99

2781Mtp1	*V. vinifera*	Kibraïski	Uzbekistan	0.002	0.008	0.99
0Mtp610	*V. vinifera*	Korza erevani	Armenia	0.002	0.008	0.99
DVIT0430	*V. vinifera*	Halili beliij	Afghanistan	0.001	0.01	0.989
DVIT1070	*V. vinifera*	Kule Dary	Unknown	0.001	0.01	0.989
DVIT0569	*V. vinifera*	Dais-el-anz	Iraq	0.001	0.009	0.989
DVIT2081	*V. vinifera*	Kandhari	India	0.003	0.008	0.989
TYR VI 10-09	*V. vinifera*	Anab-E-Shaki	Unknown	0.001	0.011	0.988
Turkmn 3033	*V. vinifera*	Gurgon	Turkmenistan	0.002	0.01	0.988
1985-0-2415-S1	*V. vinifera*	Tufachi S1	Israel	0.002	0.01	0.988
2076Mtp1	*V. vinifera*	A Kalatchel	Iran	0.001	0.01	0.988
DVIT1982	*V. vinifera*	Dabouki	Israel	0.002	0.011	0.987
0Mtp414	*V. vinifera*	Kizil sapak	Turkmenistan	0.002	0.011	0.987
2086Mtp1	*V. vinifera*	Blanc d'Iran (Charif)	Iran	0.001	0.011	0.987
DVIT0337	*V. vinifera*	Alloued Zeine	Lebanon	0.003	0.011	0.986
2659Mtp1	*V. vinifera*	Tagobi	Tajikistan	0.001	0.014	0.985
2657Mtp1	*V. vinifera*	Soïaki	Uzbekistan	0.002	0.013	0.985
DVIT2171	*V. vinifera*	B 15-19	Unknown	0.002	0.014	0.984
Turkmn 13387	*V. vinifera*	DK #11	Turkmenistan	0.001	0.014	0.984
Turkmn 6987	*V. vinifera*	Sary Aygyr	Turkmenistan	0.001	0.017	0.982
Turkmn 3036	*V. vinifera*	Kara Uzyum Nuhurskii	Turkmenistan	0.001	0.018	0.981
DVIT0685	*V. vinifera*	Charas	USSR	0.001	0.017	0.981
Turkmn 19806	*V. vinifera*	Hiv Uzyum	Turkmenistan	0.005	0.014	0.981

DVIT2533	*V. vinifera*	Nosargoon	Pakistan	0.002	0.019	0.98
Turkmn 13386	*V. vinifera*	DK #12	Turkmenistan	0.001	0.019	0.98
DVIT2272	*V. vinifera*	Pakistan Collection 25237	Pakistan	0.001	0.02	0.979
2845Mtp1	*V. vinifera*	Itchkimar biely faux (Collection Kichinev)	Uzbekistan	0.004	0.017	0.979
Turkmn 3030	*V. vinifera*	Mellei	Turkmenistan	0.001	0.021	0.978
2982Mtp1	*V. vinifera*	Ahmeh Sal apyrène	Iran	0.002	0.02	0.978
Turkmn 13360	*V. vinifera*	DK #03	Turkmenistan	0.002	0.02	0.978
Turkmn 13389	*V. vinifera*	DK #2	Turkmenistan	0.002	0.02	0.978
DVIT0606	*V. vinifera*	Zerk	Iraq	0.004	0.018	0.978
Turkmn 545	*V. vinifera*	Kizil Sapak	Turkmenistan	0.001	0.022	0.977
DVIT0604	*V. vinifera*	Yaghotti No. 1	Iran	0.001	0.023	0.976
1979-0-2219-S1	*V. vinifera*	A'asemi S1	Yemen	0.003	0.02	0.976
2654Mtp1	*V. vinifera*	Ranny Vira	Russia	0.009	0.015	0.976
2074Mtp1	*V. vinifera*	Siah	Iran	0.002	0.024	0.975
746Mtp1	*V. vinifera*	Kisil sapak	Russia	0.001	0.025	0.974
Turkmn 30743	*V. vinifera*	DK N15 (#15)	Turkmenistan	0.003	0.024	0.973
DVIT0384	*V. vinifera*	Coudsi	Unknown	0.002	0.026	0.971
DVIT0388	*V. vinifera*	Dabouki	Israel	0.003	0.027	0.97
DVIT2452	*V. vinifera*	Kwar II	Pakistan	0.018	0.012	0.97
DVIT2072	*V. vinifera*	Uzbekistanian Muscat	USSR	0.006	0.025	0.969
DVIT0358	*V. vinifera*	Baidh Ul Haman	Unknown	0.002	0.031	0.966

2679Mtp1	*V. vinifera*	Yumalak lelyi	Uzbekistan	0.012	0.022	0.966
2897Mtp1	*V. vinifera*	Ak ouzioum tapapskii	Russia	0.002	0.035	0.963
1186Mtp1	*V. vinifera*	Chirai obak	Tajikistan	0.002	0.036	0.962
0Mtp828	*V. vinifera*	Long Yan	China	0.015	0.023	0.961
2854Mtp1	*V. vinifera*	Oktiabrskii	Uzbekistan	0.002	0.038	0.96
2075Mtp1	*V. vinifera*	Ozaan Daii	Iran	0.003	0.038	0.959
DVIT2604	*V. vinifera*	Schwin	Pakistan	0.002	0.041	0.957
1678Mtp5	*V. vinifera*	Kichmich rond	Turkey	0.001	0.043	0.956
DVIT0563	*V. vinifera*	Ajmi	Iraq	0.008	0.037	0.955
DVIT0612	*V. vinifera*	Ak Schekerek	Turkmenistan	0.001	0.048	0.951
DVIT0417	*V. vinifera*	Henab	Turkey	0.002	0.049	0.949
Turkmn 6971	*V. vinifera*	Halili Nobat Niyaz	Turkmenistan	0.002	0.051	0.948
156Mtp1	*V. vinifera*	Baxtiori	Uzbekistan	0.003	0.048	0.948
2090Mtp1	*V. vinifera*	Sahami	Iran	0.002	0.051	0.947
TYR VI 13-09	*V. vinifera*	Guzal Kara	Uzbekistan	0.003	0.054	0.944
Turkmn 19735	*V. vinifera*	Porsi Shekerek	Turkmenistan	0.001	0.056	0.943
DVIT2509	*V. vinifera*	Dalnato I	Pakistan	0.001	0.057	0.942
0Mtp1148	*V. vinifera*	Tscharma	Uzbekistan	0.002	0.073	0.925
DVIT0613	*V. vinifera*	Chan Isium	USSR	0.001	0.076	0.922
635Mtp1	*V. vinifera*	Hunisa	Iran	0.003	0.079	0.918
DVIT2514	*V. vinifera*	Neeli	Pakistan	0.08	0.005	0.915
2510Mtp1	*V. vinifera*	Vassarga tchernaia	Uzbekistan	0.002	0.087	0.911

DVIT2506	*V. vinifera*	Budrilay	Pakistan	0.002	0.097	0.901
2001-9-7097-01	*V. vinifera*	Shahani 01	Iran	0.002	0.109	0.89
Turkmn 21604	*V. vinifera*	Mamidon Kizil	Turkmenistan	0.001	0.109	0.89
DVIT2502	*V. vinifera*	Parargoon	Pakistan	0.002	0.113	0.886
2001-9-8101-01	*V. vinifera*	Mehdi 01	Iran	0.002	0.128	0.871
DVIT2499	*V. vinifera*	Bargoon	Pakistan	0.002	0.127	0.871
DVIT2271	*V. vinifera*	Pakistan Collection 25241	Pakistan	0.005	0.124	0.87
Turkmn 551	*V. vinifera*	Kara Uzyum Ashhabadskii	Turkmenistan	0.002	0.132	0.866
DVIT0316	*V. vinifera*	Fachren Weis	USSR	0.002	0.135	0.863
DVIT2085	*V. vinifera*	Aswad	Yemen	0.003	0.167	0.83
DVIT2505	*V. vinifera*	Boyalsing II	Pakistan	0.002	0.176	0.822
2664Mtp1	*V. vinifera*	Khindogny	Iran	0.002	0.199	0.799
2077Mtp1	*V. vinifera*	Yhsouh ali	Iran	0.002	0.204	0.794
2675Mtp1	*V. vinifera*	Chaani biely	Azerbaijan	0.002	0.208	0.79
Turkmn 6981	*V. vinifera*	Kash Uzyum	Turkmenistan	0.002	0.216	0.782
DVIT2264	*V. vinifera*	Pakistan Collection 25311	Pakistan	0.004	0.217	0.779
DVIT2511	*V. vinifera*	Namonia	Pakistan	0.098	0.125	0.776
DVIT0608	*V. vinifera*	Rhazaki Anatolico	Greece	0.001	0.254	0.745
DVIT0371	*V. vinifera*	Chaouch	Turkey	0.005	0.263	0.732
DVIT2512	*V. vinifera*	Buraburi	Pakistan	0.002	0.272	0.727
2078Mtp1	*V. vinifera*	Sahilii	Iran	0.001	0.296	0.703
1749Mtp1	*V. vinifera*	Bayad	Yemen	0.006	0.311	0.682

DVIT2045	*V. vinifera*	Nunaka Sia	Afghanistan	0.002	0.333	0.666
DVIT2534	*V. vinifera*	Kala Kostan	Pakistan	0.31	0.028	0.662
Turkmn 13362	*V. vinifera*	DK #05	Turkmenistan	0.001	0.36	0.638
1752Mtp1	*V. vinifera*	Irki	Yemen	0.002	0.368	0.63
2001-9-8100-01	*V. vinifera*	Kondori 01	Iran	0.002	0.377	0.621
DVIT2282	*V. vinifera*	Pakistan Collection 25168	Pakistan	0.003	0.383	0.614
0Mtp1449	*V. vinifera*	Malahy	Iran	0.003	0.383	0.613
TYR VI 17-15	*V. vinifera*	Vitis Vinifera #1359	Uzbekistan	0.002	0.388	0.61
DVIT1816	*V. sylvestris*	O35-64	Iran	0.318	0.079	0.603
DVIT2683	*V. vinifera*	Norakert PRG 2224	Russia	0.033	0.408	0.558
0Mtp471	*V. vinifera*	Haita safid	Afghanistan	0.002	0.451	0.547
