# Supplementary figures and images for: Identification of mildew resistance in wild and cultivated Central Asian grape germplasm
Source: BMC Plant Biol. 2013 Oct 4;13:149. doi: 10.1186/1471-2229-13-149 (PMC3851849; doi:10.1186/1471-2229-13-149)

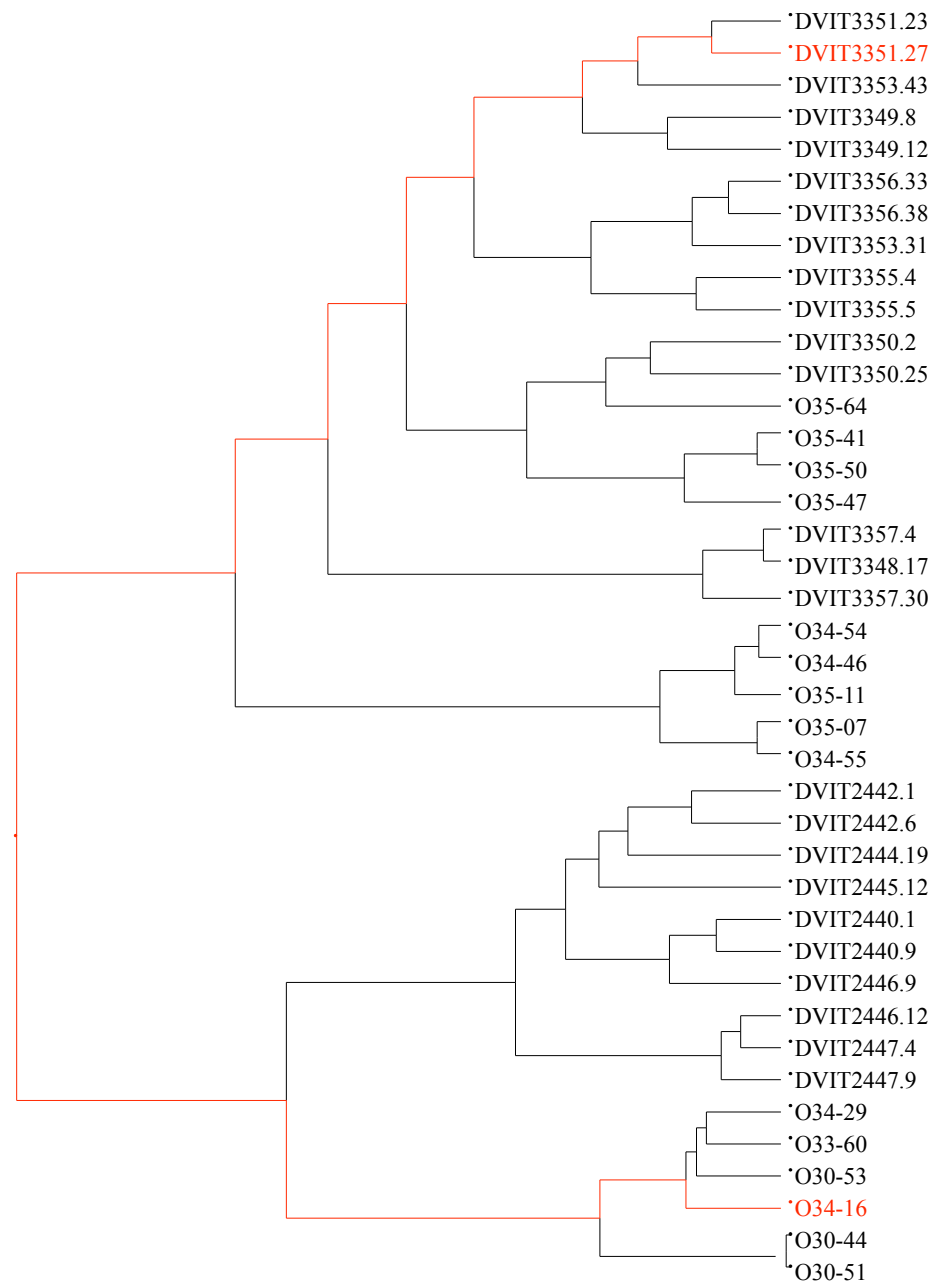

0 0.2

Supplement: Additional file 10: Figure S1 — Dendrogram of 40 V. vinifera subsp. sylvestris accessions based on hierarchal cluster analysis (Ward method). Two accessions in red font are V. vinifera subsp. sylvestris that were resistant to powdery mildew in field trials. [file 1471-2229-13-149-S10.pdf]
