# Supplementary material for: Identification of mildew resistance in wild and cultivated Central Asian grape germplasm
Source: BMC Plant Biol. 2013 Oct 4;13:149. doi: 10.1186/1471-2229-13-149 (PMC3851849; doi:10.1186/1471-2229-13-149)
Supplement: Additional file 11: Table S10 — Determination of flower phenotype and genotype. APT3 gene marker distinguished females (F) from males (M) or hermaphrodites (H). SSR marker VVIb23 is tightly linked to the flower sex locus and the unique allele 282 is linked to hermaphrodism. With the combination of these two markers, the sex phenotype of a grapevine could be determined without prior knowledge. All bold and italicized flower phenotypes for samples from the Vassal collection were verified with field data. [file 1471-2229-13-149-S11.pdf]

Supplementary Table S10. Determination of flower phenotype and genotype. APT3 gene marker distinguished females (F) from males (M) or hermaphrodites (H). SSR marker VVIb23 is tightly linked to the flower sex locus and the unique allele 282 is linked to hermaphrodism. With the combination of these two markers, the sex phenotype of a grapevine could be determined without prior knowledge. All bold and italicized flower phenotypes for samples from the Vassal collection were verified with field data.

Accession ID	Species	Accession name	Source country	Flower phenotype	Flower genotype	APT3a	APT3b	APT3c	APT3d	VVIb23a	VVIb23b
PI# 588421	*V. yenshanensis*	588421.a	China	H	HF	266	397	466		272	282
PI# 588422	"	588422.a	China	M	MF or MH	**336**	466			277	288
588650	"	588650.a	China	Undetermined		MD				MD	
597294.01	*Vitis* species	A-166-003	China	M	MF or MH	256	266	388	397	272	272
GVIT 814	*V. amurensis*	588452.b	USSR	M	MF or MH	266	397	462		284	294
DVIT1158.1	"		China	M	MF or MH	**336**	466			284	291
DVIT1158.4	"		China	M	MF or MH	**336**	466			284	291
DVIT1157.12	"		China	M	MF or MH	**336**	466			284	288
DVIT1157.2	"		China	F	FF	**336**	**336**			288	290
DVIT2005.5	"		China	M	MF or MH	**336**	466			288	288
DVIT1156.2	"		China	M	MF or MH	**336**	466			284	290
DVIT2006.1	"		South Korea	M	MF or MH	266	397	466		284	294
597298.01	*Vitis* species	B-166-016	China	H	HF	**336**	466			277	282
588715.01	"	B-166-019	China	Undetermined		MD				MD	
DVIT2596.1	*V. betulifolia*		China	H	HF	**336**	466			282	290
597296.01	*V. romanetii*	C-166-025	China	M?	MF or MH	**336**	466			282	282
DVIT2550	"	C-166-026	China	F	FF	266	266			280	280
DVIT3192	"	C-166-043	China	M	MF or MH	266	**336**	397	466	280	286
DVIT1159.9	*V. coignetiae*		Unknown	F	FF	266	**336**			284	291
DVIT1159.3	"		Unknown	M?	MF or MH	266	462			282	286
DVIT1159.10	"		Unknown	F	FF	266	**336**	397		288	294
DVIT2008.5	*V. ficifolia*		South Korea	M	MF or MH	266	**336**	397	466	272	278
DVIT2008.7	"		South Korea	M	MF or MH	266	397	466		278	294
DVIT1160.7	"		China	M	MF or MH	266	397	466		278	294

DVIT1385	*V. flexousa*			Unknown	M	MF or MH				276	276
597295.01	*Vitis* species	J-167-048		China	M	MF or MH	266	397	466	272	288
DVIT2349.13	*V. jacquemontii*			Pakistan	H	HF	466	466		282	282
DVIT2355.11	"			Pakistan	H	HF	466	466		282	282
DVIT2354.7	"			Pakistan	H	HF	266	466		282	282
TYR VI 13-17	"	Jane De Smirna		Unknown	H	HF	266	397	466	282	299
DVIT2539	"	Kali Dakh II		Pakistan	H	HF	266	397		282	299
DVIT1815	*V. lanata*	O35-59		Afghanistan	M	MF or MH	266	466		284	310
DVIT2027	*V. piasezkii*			Asia	M	MF or MH	**<u>336</u>**	466		280	286
DVIT2032	"			Asia	M	MF or MH	466	484		280	290
GVIT 859	"		597257.03	China	M	MF or MH	**<u>336</u>**	466	484	288	288
2076Mtp1	*V. vinifera*	A Kalatchel		Iran	***H***	HF	266	466		282	288
1979-0-2219-S1	"	A'asemi S1		Yemen	H	HF	266	397	466	282	288
DVIT2040	"	Ab Jusht		Afghanistan	H	HF	266	397	466	282	303
1748Mtp1	"	Abjouch		Afghanistan	***F***	FF	266	397			
DVIT0305	"	Abla Aganin Isium		USSR	Undetermined		266	397			
TYR VI 10-03	"	Afuz-Ali Urmasti		Unknown	H	HF	266	466		282	288
2842Mtp1	"	Ag Kiourdach p.e.		Azerbaijan	***H***	HF	266	397	466	282	288
TYR VI 10-05	"	Agaday		Unknown	H	HF	266	397	466	282	299
2982Mtp1	"	Ahmeh Sal apyrène		Iran	***H***	HF	266	397	466	282	299
DVIT0563	"	Ajmi		Iraq	H	HF	266	397	466	282	288
2897Mtp1	"	Ak ouzioum tapapskii		Russia	***H***	HF	266	397	466	282	288
DVIT0612	"	Ak Schekerek		Turkmenistan	H	HF	266	397	466	282	288
DVIT0307	"	Al Borla		USSR	H	HF	266	397	466	282	307
DVIT0634	"	Alburla		USSR	H	HF	266	397	466	282	307
Turkmn 19652	"	Ali Shaitan		Turkmenistan	F	FF	266	397		288	299
2858Mtp1	"	Alia boka		Uzbekistan	***F***	FF	266	397		288	288
DVIT0337	"	Alloued Zeine		Lebanon	H	HF	266	397	466	282	282

1183Mtp1	″	Alü tierskü	Russia	***H***	HF	266	397	466	282	307
DVIT0338	″	Alulu	Iraq	F	FF	266	397		302	307
0Mtp27	″	Anab-e-Shabi	India	***H***	HF	266	397	466	282	288
TYR VI 10-09	″	Anab-E-Shaki	Unknown	H	HF	266	397	466	282	288
2113Mtp1	″	Aragatzi	Armenia	***F***	FF	266	266		299	299
2001-9-8093-01	″	Asgari 01	Iran	H	HF	266	397	466	282	288
DVIT0343	″	Askari	Afghanistan	H	HF	466	466		282	282
2088Mtp1	″	Askari noir	Iran	***H***	HF	266	466		282	299
2083Mtp1	″	Askudi	Iran	***H***	HF	266	466			
DVIT0308	″	Asma	USSR	H	HF	266	397	466	282	307
2505Mtp1	″	Assylkara	Russia	**H**	HF	266	397	466	282	288
DVIT2085	″	Aswad	Yemen	H	HF	266	397	466	282	307
DVIT2171	″	B 15-19	Unknown	H	HF	266	397	466	282	307
DVIT2306	″	Baharat Early	India	H	HF	266	397	466	282	288
2271Mtp2	″	Baïan chirei	Azerbaijan	**H**	HF	466	466		282	282
DVIT0358	″	Baidh Ul Haman	Unknown	H	HF	266	397	466	282	288
DVIT2499	″	Bargoon	Pakistan	F	FF	397	397		288	299
DVIT0310	″	Barmak Isium	Turkey	Undetermined		266	397	466		
156Mtp1	″	Baxtiori	Uzbekistan	Undetermined		266	466			
1749Mtp1	″	Bayad	Yemen	***H***	HF	266	466		282	288
DVIT1841	″	Beli Potok	Yugoslavia	F	FF	266	397		286	286
DVIT2501	″	Berurargoon	Pakistan	F	FF	266	397		288	288
1981-0-2233-S1	″	Bez el Anza S1	Egypt	H	HF	466	466		282	282
DVIT0311	″	Bias Kukuzeti	USSR	H	HF	266	397	466	282	307
DVIT2055	″	Black Kishmish	Russia	H	HF	266	397	466	282	288
2086Mtp1	″	Blanc d'Iran (Charif)	Iran	***H***	HF	266	397	466	282	288
DVIT0312	″	Blanc De Crimei	USSR	H	HF	266	397	466	282	307
2507Mtp1	″	Bouaki nor	Uzbekistan	***H***	HF	266	397	466	282	299

2509Mtp1	″	Boulany	Russia	***H***	HF	266	397	466	282	307
DVIT2496	″	Boyalsing I	Pakistan	H	HF	266	397	466	282	299
DVIT2505	″	Boyalsing II	Pakistan	H	HF	266	397	466	282	299
DVIT0673	″	Buaki	Russia	H	HF	266	397	466	282	288
DVIT2506	″	Budrilay	Pakistan	F	FF	266	397		288	299
DVIT2512	″	Buraburi	Pakistan	H	HF	266	397	466	282	299
TYR VI 11-13	″	Caus X Pearl Csaba	Yugoslavia	H	HF	266	397	466	282	288
2675Mtp1	″	Chaani biely	Azerbaijan	***F***	FF	266	266		299	299
2676Mtp1	″	Chaani noir	Azerbaijan	***H***	HF	266	397	466	282	299
2677Mtp2	″	Chakar angour de Tachkent	Uzbekistan	Undetermined		266	266			
DVIT2730	″	Chamo	Pakistan	Undetermined		266	397	466		
DVIT0613	″	Chan Isium	USSR	F	FF	266	397		299	303
DVIT0371	″	Chaouch	Turkey	F	FF	266	397		288	307
DVIT0685	″	Charas	USSR	F	FF	266	397		307	307
0Mtp225	″	Chassany	Azerbaijan	***F***	FF	266	397		299	307
2678Mtp2	″	Chirvan chakki	Azerbaijan	***H***	HF	266	397	466	282	299
Turkmn 16392	″	Chol Uzyum	Turkmenistan	H	HF	266	397	466	282	299
2857Mtp1	″	Chtour angour	Uzbekistan	***H***	HF	266	466		282	288
DVIT2532	″	Churgoon	Pakistan	H	HF	266	397	466	282	288
DVIT0313	″	Cirmisi Sap De Sudak	USSR	F	FF	266	397		299	307
DVIT0384	″	Coudsi	Unknown	H	HF	266	397	466	282	288
DVIT1982	″	Dabouki	Israel	H	HF	266	397	466	282	288
DVIT0388	″	Dabouki	Israel	H	HF	266	397	466	282	288
DVIT0569	″	Dais-el-anz	Iraq	H	HF	266	397	466	282	299
DVIT2509	″	Dalnato I	Pakistan	F	FF	266	397		288	288
DVIT2536	″	Dardari	Pakistan	F	FF	266	397		288	288
857Mtp1	″	De Hengril	Russia	***H***	HF	266	397	466	282	299
DVIT0314	″	Demir Kara	USSR	H	HF	266	397	466	282	288

Turkmn 13358	″	DK #01	Turkmenistan	H	HF	**336**	466			282	282
Turkmn 13359	″	DK #02	Turkmenistan	H	HF	266	397			282	299
Turkmn 13360	″	DK #03	Turkmenistan	F	FF	266	397			288	303
Turkmn 13361	″	DK #04	Turkmenistan	H	HF	266	397	466		282	288
Turkmn 13362	″	DK #05	Turkmenistan	F	FF	266	397			288	288
Turkmn 13364	″	DK #07	Turkmenistan	M?	MF or MH	266	**336**	397	466	299	303
Turkmn 13365	″	DK #08	Turkmenistan	M?	MF or MH	266	**336**	397	466	299	303
Turkmn 13388	″	DK #10	Turkmenistan	Undetermined		266	266				
Turkmn 13387	″	DK #11	Turkmenistan	H	HF	266	**336**	466		282	288
Turkmn 13386	″	DK #12	Turkmenistan	H	HF	266	397	466		282	288
Turkmn 13381	″	DK #17	Turkmenistan	H	HF	266	**336**	397	466	282	299
Turkmn 13389	″	DK #2	Turkmenistan	F	FF	266	397			299	303
Turkmn 13379	″	DK #21 Chernyi	Turkmenistan	H	HF	266	**336**	397	466	282	299
Turkmn 13377	″	DK #39	Turkmenistan	H	HF	266	397	466		282	288
Turkmn 6272	″	DK #9	Turkmenistan	F	FF	266	397			288	299
Turkmn 6977	″	DK Belyi	Turkmenistan	H	HF	266	397	466		282	288
Turkmn 13375	″	DK Melkii Chernyi	Turkmenistan	H	HF	266	397	466		282	299
Turkmn 13374	″	DK Melkii Krasnyi	Turkmenistan	M?	MF or MH	266	**336**	397	466	288	294
Turkmn 30743	″	DK N15 (#15)	Turkmenistan	F	FF	266	266			288	299
0Mtp318	″	Doppelaugen	Azerbaijan	***F***	FF	266	397			288	299
DVIT0315	″	Dschan Im Isium	USSR	H	HF	266	397	466		282	307
Turkmn 6218	″	Ekdona Turkmenskaya	Turkmenistan	H	HF	266	397	466		282	299
DVIT0316	″	Fachren Weis	USSR	H	HF	266	397	466		282	288
DVIT0402	″	Fahri	Afghanistan	H	HF	266	397	466		282	299
DVIT2510	″	Fatai	Pakistan	H	HF	**336**	466			282	282
DVIT2636	″	Fayoumi	Egypt	H	HF	266	397	466		282	288
0000-0-4432-S1	″	Fetyaska S1	Russia	H	HF	266	397	466		282	307
DVIT2537	″	Gaschochi	Pakistan	H	HF	266	397	466		282	288

Turkmn 3028	"	Gechi Kyrlen	Turkmenistan	H	HF	266	397	466		282	303
0Mtp428	"	Golodan	Afghanistan	***H***	HF	266	397	466		282	288
0Mtp429	"	Gora Chirine faux	Iran	***H***	HF	466	466			282	282
DVIT0413	"	Gros Colman	USSR	H	HF	266	397	466		282	299
DVIT2500	"	Gungargoon	Pakistan	H	HF	266	397	466		282	299
Turkmn 3033	"	Gurgon	Turkmenistan	H	HF	266	397	466		282	288
TYR VI 13-09	"	Guzal Kara	Uzbekistan	H	HF	266	397	466		282	288
0Mtp471	"	Haita safid	Afghanistan	***H***	HF	266	466				
DVIT0430	"	Halili beliij	Afghanistan	H	HF	266	397	466		282	303
Turkmn 6971	"	Halili Nobat Niyaz	Turkmenistan	F	FF	266	397			299	303
DVIT0416	"	Hassaine	Unknown	H	HF	266	397	466		282	288
DVIT0417	"	Henab	Turkey	H	HF	266	397	466		282	288
DVIT0328	"	Himrisnky	USSR	H	HF	266	397	466		282	299
DVIT0759	"	Hisakasy	Russia	H	HF	266	397	466		282	307
Turkmn 19806	"	Hiv Uzyum	Turkmenistan	H	HF	266	397	466		282	288
DVIT2503	"	Hosargoon	Pakistan	H	HF	266	397	466		282	288
635Mtp1	"	Hunisa	Iran	***F***	FF	266	266				
TYR VI 13-15	"	Huseine Rozvoj	Unknown	H	HF	266	397	466		282	307
1752Mtp1	"	Irki	Yemen	***H***	HF	266	466			282	299
Turkmn 3026	"	Irtyk Yaprak	Turkmenistan	F	FF	266	397			299	303
2845Mtp1	"	Itchkimar biely faux	Uzbekistan	***F***	FF	266	**336**	397		288	299
DVIT2497	"	Kabuli	Pakistan	H	HF	266	466			282	288
DVIT2534	"	Kala Kostan	Pakistan	M?	MF or MH	266	**336**	397	466	278	299
DVIT0335	"	Kalamak	Afghanistan	H	HF	266	397	466		282	299
DVIT2917	"	Kali Sag	Unknown	H	HF	266	397	466		282	299
DVIT0428	"	Kandahar	Unknown	H	HF	266	397	466		282	299
DVIT2081	"	Kandhari	India	H	HF	266	397	466		282	299
0Mtp565	"	Kanfet isium	Russia	***H***	HF	266	397	466		282	299

DVIT2322	″	Kara Dzhidzhigi	Uzbekistan	H	HF	266	397	466		282	288
2690Mtp1	″	Kara Kaltak	Uzbekistan	***H***	HF	266	397	466		282	288
DVIT0773	″	Kara Lakana	USSR	H	HF	266	397	466		282	288
2780Mtp1	″	Kara Palvan	Uzbekistan	Undetermined		266	266				
Turkmn 6982	″	Kara Terbash	Turkmenistan	H	HF	266	397	466		282	288
Turkmn 551	″	Kara Uzyum Ashhabadskii	Turkmenistan	H	HF	266	397	466		282	288
Turkmn 3036	″	Kara Uzyum Nuhurskii	Turkmenistan	M?	MF or MH	266	**336**	397	466	299	303
Turkmn 21551	″	Karga Dili	Turkmenistan	H	HF	266	397	466		282	299
Turkmn 6981	″	Kash Uzyum	Turkmenistan	F	FF	266	397			288	299
DVIT2451	″	Kashiri	Pakistan	H	HF	266	397	466		282	288
DVIT0774	″	Katta Kurgan	USSR	F	FF	266	266			288	299
2951Mtp1	″	Kaytagi	Russia	***F***	FF	266	397			288	307
1679Mtp2	″	Kechmisch aly violet	Iran	***H***	HF	266	466			282	299
Turkmn 29892	″	Keshmesh Heshrau	Turkmenistan	H	HF	266	397	466		282	299
DVIT0432	″	Khaldar	Afghanistan	H	HF	266	397	466		282	299
DVIT2084	″	Khalili	Afghanistan	F	FF	266	397			288	299
2663Mtp2	″	Khalili tcherni	Iran	***H***	HF	266	**336**	397	466	282	307
2190Mtp1	″	Khatmi	Russia	***H***	HF	266	397	466		282	299
DVIT2919	″	Khawngi	India	H	HF	**336**	466			278	282
2664Mtp1	″	Khindogny	Iran	***H***	HF	266	466			282	299
DVIT0434	″	Khir Ghulaman	Afghanistan	H	HF	266	397	466		282	299
DVIT2605	″	Khorestini	Pakistan	F	FF	266	266			288	288
2781Mtp1	″	Kibraïski	Uzbekistan	***H***	HF	266	466			282	307
1678Mtp5	″	Kichmich rond	Turkey	***H***	HF	266	466			282	307
DVIT2495	″	Kini Yatch	Pakistan	H	HF	266	397			282	299
DVIT2071	″	Kishmish Of Vir	USSR	H	HF	266	397	466		282	299
DVIT0437	″	Kishmish Sorkh	Afghanistan	H	HF	266	397	466		282	299
TYR VI 14-19	″	Kishmishi	Unknown	F	FF	266	397			288	299

DVIT0435	″	Kishmishi	Afghanistan	H	HF	266	466		282	299
0Mtp589	″	Kisil izium	Russia	***H***	HF	266	466		282	303
746Mtp1	″	Kisil sapak	Russia	***H***	HF	266	397	466	282	299
Turkmn 19697	″	Kismish Turkmenskii	Turkmenistan	H	HF	266	397	466	282	303
0Mtp414	″	Kizil sapak	Turkmenistan	***H***	HF	266	466		282	303
Turkmn 545	″	Kizil Sapak	Turkmenistan	H	HF	266	397	466	282	303
2001-9-8100-01	″	Kondori 01	Iran	H	HF	266	397	466	282	299
DVIT0783	″	Koptcha	Russia	H	HF	**336**	466		282	307
0Mtp610	″	Korza erevani	Armenia	***H***	HF	266	397	466	282	307
DVIT2680	″	Kouldjinski	Russia	H	HF	266	397	466	282	288
DVIT0329	″	Kovalewka	USSR	H	HF	266	397	466	282	288
2635Mtp1	″	Koz ouzioum	Russia	***F***	FF	266	397		299	307
DVIT1070	″	Kule Dary	Unknown	H	HF	266	397	466	282	288
Turkmn 18820	″	Kush Dzhumurtka	Turkmenistan	H	HF	266	397	466	282	303
DVIT2452	″	Kwar II	Pakistan	H	HF	266	397	466	282	288
DVIT0442	″	Lal Sorkh	Afghanistan	F	FF	266	397		288	299
DVIT2641	″	Leanoy	USSR	H	HF	266	397	466	282	299
0Mtp1475	″	Liali bidona	Azerbaijan	***F***	FF	266	397		299	299
0Mtp640	″	Liali Yakdona	Kazakhstan	***H***	HF	266	397	466	282	299
2640Mtp1	″	Lkeni noir	Azerbaijan	***H***	HF	266	397	466	282	299
0Mtp828	″	Long Yan	China	***H***	HF	266	397	466	282	299
0Mtp1449	″	Malahy	Iran	***F***	FF	266	266		286	299
Turkmn 3025	″	Mamidon	Turkmenistan	H	HF	266	397	466	282	303
Turkmn 6984	″	Mamidon Deli	Turkmenistan	H	HF	266	397	466	282	288
Turkmn 21604	″	Mamidon Kizil	Turkmenistan	H	HF	266	397	466	282	288
0Mtp703	″	Matrassa	Russia	***H***	HF	266	397	466	282	299
2001-9-8101-01	″	Mehdi 01	Iran	H	HF	266	466		282	299
Turkmn 3030	″	Mellei	Turkmenistan	H	HF	266	397	466	282	303

DVIT1042	″	Mermark	Iraq	H	HF	266	397	466		282	288
0Mtp1795	″	Mesisti rose	Russia	***H***	HF	266	397	466		282	299
DVIT2498	″	Millishun	Pakistan	H	HF	266	**336**	397	466	282	282
1742Mtp1	″	Monaca	Afghanistan	***F***	FF	266	266			299	299
DVIT0462	″	Monukka	Afghanistan	H	HF	266	397	466		282	299
0Mtp750	″	Mouchketny	Russia	***H***	HF	266	466			282	307
DVIT0319	″	Mourvedre Famellestadt	USSR	H	HF	266	397	466		282	282
DVIT0320	″	Murma Isium	USSR	H	HF	266	397	466		282	288
TYR VI 15-13	″	Mzivani	Unknown	H	HF	266	397	466		282	307
DVIT2511	″	Namonia	Pakistan	H	HF	266	397	466		282	288
2648Mtp2	″	Narma	Russia	***H***	HF	266	397	466		282	299
0Mtp780	″	Nassau	Russia	***H***	HF	266	466			282	288
DVIT2514	″	Neeli	Pakistan	***F?***	FF	266	266			282	288
DVIT2507	″	Neelilay	Pakistan	F	FF	266	397			288	299
TYR VI 15-17	″	Nimrang	Russia	F	FF	266	397			288	299
2736Mtp1	″	Nimrang rouge	Uzbekistan	***H***	HF	266	397	466		282	299
DVIT0330	″	Noir D'automne	USSR	H	HF	266	397	466		282	307
DVIT2683	″	Norakert PRG 2224	Russia	H	HF	**336**	466			282	303
DVIT2533	″	Nosargoon	Pakistan	F	FF	266	397			288	299
2649Mtp1	″	Noulizok	Uzbekistan	***F***	FF	266	266			288	288
DVIT2045	″	Nunaka Sia	Afghanistan	H	HF	266	397	466		282	282
2854Mtp1	″	Oktiabrskii	Uzbekistan	***H***	HF	266	397	466		282	299
Turkmn 30748	″	Orion	Turkmenistan	H	HF	**336**	466			282	294
2952Mtp1	″	Otcha bala	Uzbekistan	***H***	HF	266	397	466		282	307
2075Mtp1	″	Ozaan Daii	Iran	***H***	HF	466	466			282	282
DVIT2282	″	Pakistan Collection 25168	Pakistan	F	FF	266	397			288	299
DVIT2272	″	Pakistan Collection 25237	Pakistan	F	FF	266	266			288	288
DVIT2271	″	Pakistan Collection 25241	Pakistan	H	HF	266	397	466		282	288

DVIT2293	″	Pakistan Collection 25265	Pakistan	H	HF	266	**336**	397	466	282	282
DVIT2263	″	Pakistan Collection 25275	Pakistan	H	HF	266	397	466		282	288
DVIT2296	″	Pakistan Collection 25290	Pakistan	H	HF	266	**336**	397	466	282	288
DVIT2283	″	Pakistan Collection 25296	Pakistan	H	HF	266	397	466		282	288
DVIT2264	″	Pakistan Collection 25311	Pakistan	F	FF	266	397			288	288
DVIT2502	″	Parargoon	Pakistan	F	FF	266	**336**	397		288	307
2691Mtp1	″	Parkent	Uzbekistan	***H***	HF	266	466			282	288
2597Mtp1	″	Peikani	Iran	***H***	HF	266	397	466		282	307
DVIT2755	″	Persian R27	Unknown	H	HF	**336**	466			278	282
2651Mtp2	″	Pervenetz Praskoveisky	Russia	***H***	HF	266	397	466		282	307
2788Mtp1	″	Pinger putao	China	***H***	HF	266	466			282	299
Turkmn 19735	″	Porsi Shekerek	Turkmenistan	F	FF	266	397			288	299
0Mtp928	″	Précoce d'Astrakan	Iran	***F***	FF	266	397			288	307
0Mtp950	″	Rajoulan	Russia	***H***	HF	266	466			282	307
2654Mtp1	″	Ranny Vira	Russia	***H***	HF	266	397	466		282	307
2737Mtp1	″	Razakiia piembiana	Russia	***H***	HF	266	397	466		282	307
1750Mtp1	″	Raziki	Yemen	***H***	HF	266	397	466		282	288
DVIT0499	″	Red Ohanez	Russia	H	HF	266	397	466		282	299
TYR VI 16-09	″	Rhazaki (Pa 1882)	Unknown	H	HF	266	397	466		282	288
DVIT0608	″	Rhazaki Anatolico	Greece	H	HF	266	466			282	307
DVIT0501	″	Rhazaki De Crete	Greece	H	HF	266	397	466		282	288
DVIT0555	″	Rhazaki Mavro	Unknown	H	HF	266	397	466		282	288
HOP L04-19	″	Rish Baba	Unknown	H	HF	266	397	466		282	299
DVIT2338	″	Rizamat	Uzbekistan	H	HF	266	397	466		282	299
2783Mtp1	″	Rouchaki	USSR	***H***	HF	466	466			282	282
0Mtp1007	″	Sabza angur	Tajikistan	***H***	HF	266	397	466			
2090Mtp1	″	Sahami	Iran	***H***	HF	466	466			282	282
0Mtp587	″	Sahebi	Afghanistan	***H***	HF	266	397	466		282	299

DVIT0509	″	Sahibi	Afghanistan	F	FF	266	397		299	299
DVIT0510	″	Sahibi Sorkh	Afghanistan	H	HF	266	397	466	282	288
2078Mtp1	″	Sahilii	Iran	***H***	HF	266	466			
0Mtp1010	″	Saïd guliami	Uzbekistan	***F***	FF	266	**336**		299	307
DVIT0597	″	Salomani	Iraq	H	HF	266	266		282	288
Turkmn 6987	″	Sary Aygyr	Turkmenistan	Undetermined		266	397	466		
0Mtp1031	″	Sary Kiriak	Azerbaijan	***H***	HF	266	397	466	282	288
388Mtp2	″	Sateni tcherny	Armenia	***H***	HF	466	466		282	282
1737Mtp1	″	Schiradzouli violet	Iran	***H***	HF	466	466		282	282
DVIT2604	″	Schwin	Pakistan	F	FF	266	397		299	307
DVIT1152	″	Selection 2798 (seedless)	Yugoslavia	H	HF	266	466		282	288
DVIT2685	″	Sev Lernatu PRG 2224	Russia	Undetermined		MD			MD	
2001-9-7097-01	″	Shahani 01	Iran	H	HF	**336**	466		282	282
TYR VI 16-21	″	Shakar Angur	USSR	H	HF	266	397	466	282	299
DVIT2603	″	Shingargoon	Pakistan	H	HF	266	397	466	282	299
2079Mtp1	″	Shirazi	Iran	***H***	HF	466	466		282	282
DVIT2168	″	Shtur Angur	USSR	H	HF	266	466		282	282
2074Mtp1	″	Siah	Iran	***H***	HF	266	466			
0Mtp1071	″	Soultani	Uzbekistan	***H***	HF	266	466			
2856Mtp1	″	Sourkhak biely	Uzbekistan	***F***	FF	266	266		288	299
0Mtp1073	″	Starinky	Russia	***H***	HF	266	397	466	282	288
0Mtp1597	″	Sultanine noire faux	Uzbekistan	***H***	HF	266	397	466	282	299
Turkmn 6304	″	Sumbarskii Bekyi	Turkmenistan	H	HF	266	397	466	282	288
2659Mtp1	″	Tagobi	Tajikistan	***F***	FF	266	266		288	307
DVIT2174	″	Taifi	USSR	H	HF	266	397	466	282	299
TYR VI 17-03	″	Taka Sago	Japan	H	HF	266	397	466	280	282
DVIT2928	″	Tana-Kuzi	Unknown	H	HF	266	397	466	282	299
TYR VI 17-07	″	Tarnau	USSR	H	HF	266	397	466	282	299

1218Mtp1	″	Tavkveri	Azerbaijan	***F***	FF	266	397			286	288
664Mtp1	″	Tchatyrbac	Uzbekistan	***H***	HF	266	266			282	299
DVIT2688	″	Tchilar	Russia	H	HF	466	466			282	282
2671Mtp1	″	Tchiliaki belyi	Tajikistan	***H***	HF	266	466			282	288
	″	Thompson Seedless		H	HF	266	397	466		282	299
1985Mtp2	″	Tolstokory	Russia	***F***	FF	266	397			299	299
DVIT2531	″	Torgoon	Pakistan	H	HF	266	397	466		282	288
0000-0-2411-S1	″	Trapanlarin kara S1	USSR	H	HF	266	397			282	288
0Mtp1148	″	Tscharma	Uzbekistan	***H***	HF	266	466			282	288
1985-0-2415-S1	″	Tufachi S1	Israel	H	HF	266	466			282	288
2760Mtp1	″	Tuia-tiche	Uzbekistan	***H***	HF	266	397	466		282	299
654Mtp1	″	Tuia-tiche p.e.	Russia	***F***	FF	266	397			288	299
TYR VI 17-11	″	Uzbekistan Muscat	Unknown	F	FF	266	397			286	299
DVIT2072	″	Uzbekistanian Muscat	USSR	H	HF	266	466			282	288
0Mtp1165	″	Varuschkin	Russia	***H***	HF	266	397	466		282	286
TYR VI 17-15	″	Vitis Vinifera #1359	Uzbekistan	H	HF	266	397	466		282	307
1983-0-2424-S1	″	Voivoginiova S1	USSR	H	HF	266	397	466		282	299
TYR VI 17-17	″	Volgo Don	Uzbekistan	H	HF	266	397	466		282	288
DVIT0604	″	Yaghotti No. 1	Iran	H	HF	266	397	466		282	299
2687Mtp1	″	Yaï izium rose	Russia	***H***	HF	266	397	466		282	307
TYR VI 17-19	″	Yarghouti	Unknown	H	HF	266	397	466		282	288
2077Mtp1	″	Yhsouh ali	Iran	***F?***	FF	266	**336**	397		282	299
2679Mtp1	″	Yumalak lelyi	Uzbekistan	***H***	HF	266	466				
DVIT0606	″	Zerk	Iraq	H	HF	266	397	466		282	288
2666Mtp1	″	Zimliansky noir	Russia	***H***	HF	266	397	466		282	288
DVIT2664	″	Zimsko Belo	Yugoslavia	H	HF	266	397	466		282	307
