# Supplementary material for: Identification of mildew resistance in wild and cultivated Central Asian grape germplasm
Source: BMC Plant Biol. 2013 Oct 4;13:149. doi: 10.1186/1471-2229-13-149 (PMC3851849; doi:10.1186/1471-2229-13-149)
Supplement: Additional file 12: Figure S2 — Seeds of ten accessions from the Olmo-series V. vinifera subsp. sylvestris; the last two samples have seeds with a fragile cap on small beaks. [file 1471-2229-13-149-S12.pdf]

1) O33-60 (DVIT1802)

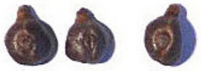

White fruit, hermaphrodite, collected from Shirvan, Iran.

2) O34-16 (DVIT1803)

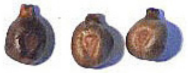

Black fruit, female, collected from Shirvan, Iran.

3) O34-26 (DVIT1805)

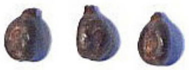

Black fruit, **male** based on the allelic pattern with APT3 and VVIb23 markers, carried wild 336 (bp) allele, collected from unknown location in Iran.

4) O34-29 (DVIT1804)

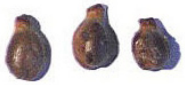

Black fruit, hermaphrodite, collected from Shirvan, Iran.

5) O34-55 (DVIT1807)

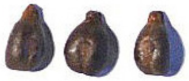

Black fruit, **male** based on the allelic pattern with APT3 and VVIb23 markers, carried wild 336 (bp) allele , collected from unknown location in Iran.

6) O35-07 (DVIT1808)

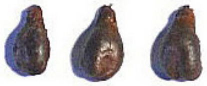

Black fruit, female**,** carried wild 336 (bp) allele , collected from unknown location in Iran.

7) O35-41 (DVIT1811)

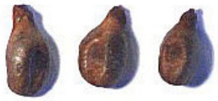

White fruit, female, collected from unknown location in Iran.

8) O35-47 (DVIT1812)

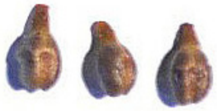

Black fruit, **male** based on the allelic pattern with APT3 and VVIb23 markers, collected from unknown location in Iran.

9) O35-50 (DVIT1813)

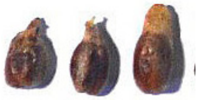

White fruit, hermaphrodite, collected from Iran.

10) O35-64 (DVIT1816)

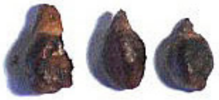

White fruit, hermaphrodite, collected from Iran.
